# Analysis of Conformational Variation in Macromolecular Structural Models

**DOI:** 10.1371/journal.pone.0039993

**Published:** 2012-07-09

**Authors:** Sandeep Kumar Srivastava, Savitha Gayathri, Babu A. Manjasetty, Balasubramanian Gopal

**Affiliations:** 1 Molecular Biophysics Unit, Indian Institute of Science, Bangalore, India; 2 European Molecular Biology Laboratory, Grenoble Outstation and Unit of Virus Host-Cell Interactions (UVHCI), Grenoble, France; Semmelweis University, Hungary

## Abstract

Experimental conditions or the presence of interacting components can lead to variations in the structural models of macromolecules. However, the role of these factors in conformational selection is often omitted by *in silico* methods to extract dynamic information from protein structural models. Structures of small peptides, considered building blocks for larger macromolecular structural models, can substantially differ in the context of a larger protein. This limitation is more evident in the case of modeling large multi-subunit macromolecular complexes using structures of the individual protein components. Here we report an analysis of variations in structural models of proteins with high sequence similarity. These models were analyzed for sequence features of the protein, the role of scaffolding segments including interacting proteins or affinity tags and the chemical components in the experimental conditions. Conformational features in these structural models could be rationalized by conformational selection events, perhaps induced by experimental conditions. This analysis was performed on a non-redundant dataset of protein structures from different SCOP classes. The sequence-conformation correlations that we note here suggest additional features that could be incorporated by *in silico* methods to extract dynamic information from protein structural models.

## Introduction

The substantial improvement in the methodology of protein structure determination is reflected by an exponential increase in the number of structures deposited in the Protein Data Bank (PDB) [1]. Functional annotation and mechanistic interpretations of several of these structural models, however, remains a significant hurdle. Information on protein dynamics and conformational variations is an important input for mechanistic interpretation. While this information is experimentally captured by Nuclear Magnetic Resonance (NMR) spectroscopy methods, structural models determined by X-Ray crystallography have to be further subjected to intensive computational methods for dynamic information. *In silico* strategies to obtain dynamic information are both time-consuming and have an inherent limitation as they do not explicitly incorporate experimental errors and artifacts induced by experimental conditions. While experimental errors can, in principle, be incorporated in computational simulations, these require access to unprocessed experimental data that is not currently freely available to analyze. Experimental conditions, on the other hand, are available either with the structural coordinates or in manuscripts that describe macromolecular structures in more detail. An examination of protein structural models along with experimental conditions could potentially aid in de-convoluting conformational selection induced during the structure determination process.

It is increasingly apparent that a single structural model of a protein is likely to be incomplete in its information content- given that it provides a single representation of several flexible segments and alternative conformations. It is thus imperative to de-convolute the dynamics and alternate conformations from a structural model to obtain a more functionally relevant model of a biological molecule. *In silico* strategies, such as Molecular Dynamics (MD) simulations, from-CONstraints-to-COORDinates (CONCOORD) analysis or more often, normal modes analysis are employed to extrapolate dynamic motions of a protein from a single experimentally determined structural model. These techniques, however, do not explicitly incorporate features such as experimental conditions or the propensity of a protein stretch to adopt conformations other than that modeled by the experimenter. The large number of structures present in the protein data bank suggests that a systematic analysis of these parameters could form a potentially useful source of information to interpret protein structures solved at high resolution. A reliable de-convolution of dynamic information that accounts for experimental artifacts could also aid in structure-based functional annotation. Indeed, a protocol that incorporates dynamic information from small protein domains to predict structural variations in large macromolecular complexes could provide valuable mechanistic information. An essential requirement towards these goals is an estimate of the influence of experimental parameters in the selection of alternate conformations that were modeled in X-Ray crystal structures or were retained in an NMR derived structural ensemble. In this study, we examine differences between structural models that share high sequence similarity to obtain an estimate of context-dependent remodeling or conformational selection. The dataset for this analysis comprised structural models derived by X-Ray and NMR methods encompassing five Structural Classification of Proteins (SCOP) classes. Multi-protein complexes and structures of peptides determined independently and as a part of large proteins were included in this analysis. Structural variations within this data-set were examined for intrinsic (sequence-based) features as well as external (experimental) parameters. This analysis highlights structural differences and provides a dataset to test *in silico* methods to extract dynamic properties of proteins while explicitly incorporating the influence of experimental parameters on structural models.

## Results

A mechanistic interpretation of the function and regulation of a protein crucially depends on information on the dynamic motions and alternate conformations that could be adopted by its structure. An estimate of the extent of conformational variation in structural models of proteins that share high sequence similarity can provide vital inputs to incorporate alternate conformations for a given molecular model. This data, however, requires additional information to distinguish between inherent flexibility vis-à-vis structural variations that can be explained by experimental conditions. Experimental context in this case includes factors that influence conformation by virtue of interactions between polypeptide fragments, concentration dependent and osmolyte-induced effects as well as ligand interactions. A representative dataset of protein structural models was collated to examine the effect of experimental conditions on conformational selection.

### Dataset of Proteins for Comparative Analysis

**Figure 1:**
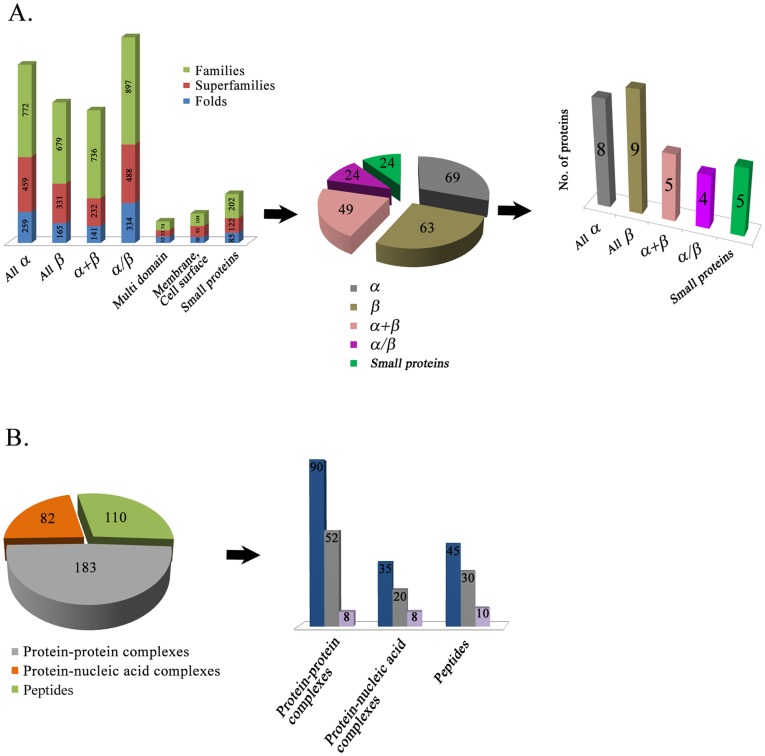
Summary of the dataset of molecular models examined for structural variations and conformational selection by experimental methods. (**A**) The initial dataset of proteins was compiled for a representative sampling of folds and families. After selecting protein-structural pairs based on experimental and sequence criteria, the dataset for analysis included 31 different protein pairs across five different structural classes. (**B**) Bar diagrams represent the protein-protein, protein-nucleic acid complexes and peptides used in this study. Dark blue bars in all the classes represent the initial selection from a set of 183 protein-protein complexes, 82 protein-nucleic acid complexes and 110 peptide structures. The final composition of this dataset (shown here in gray and light blue bars) is based on the sequence and structural criteria described in the methods section of this manuscript.

The dataset for this analysis includes high resolution crystal structures, NMR structural ensembles, protein structures that were determined in both the free-state (apo) as well as complexes with ligands or as a component of a large macromolecular complex. A pictorial description of this dataset is shown in Figure 1. This dataset incorporates all SCOP classes of proteins except membrane proteins. As there were no suitable NMR entries for multi-domain proteins and very few structures in the category of membrane and cell surface proteins, these classes were not included in this study. Protein structures were retrieved from the PDB based on folds, super-families and families which yielded a total of 1086 folds, 1777 super-families and 3464 families [2]. Further pruning based on sequence and structural criteria resulted in 233 structures spread across 5 classes of proteins viz., α, β, α+β, α/β and small proteins. A sub-set of 31 protein pairs that shared high sequence similarity but showed prominent differences in conformation were chosen for detailed analysis (Table 1, Table S1). Information on disordered proteins was obtained from the DISPROT database [3]. From this dataset of 183 protein-protein and 82 protein-nucleic acid complexes, 90 protein complexes and 35 protein-nucleic acid complexes were selected for further analysis. We found 52 protein-protein complexes and 20 protein-nucleic acid complexes that showed substantial variation in their structures between the free form, as a part of larger complexes or in some cases between different multi-protein complexes. Although peptides are not a true SCOP class, these were also included (110 structures) to examine the influence of context on structure. 45 amongst these peptide structures had an equivalent stretch (sequence identity >80%) in a larger protein (Figure 1B). The final dataset of protein complexes and peptide structures that show conformational variation are listed in Tables 2 and 3.

**Table 1:** Comparison between X-ray and NMR structures in different classes of proteins.

α-class of proteins				
*X-ray/NMR PDB*	*Identity (%)*	*Variant Region*	*Structural manifestation in crystal and solution structure*	*PSIPRED analysis*
1K96/1A03	90	42∶51 - LTIGSKLQDA	α-helix in crystal structure; Unstructured in solution	α-helix
1GU2/1E8E	100	28∶36 - ITDGKIFFN	α-helix in crystal structure; Unstructured in solution	β-strand +Coil
		48∶54 - ACASCHT	α-helix in crystal structure; Hydrogen bonded turn in solution	β-strand +Coil
		61∶70- GKNIVTGKEI	β-strand and turn in crystal structure; β- bridge in solution	β-strand +Coil
1NZN/1PC2	78.1	5∶13 - EAVLNELVSVED	α-helix in crystal structure; Unstructured in solution	α-helix
1I27/1NHA	86.6	478∶483- QTKKTGL	α-helix in crystal structure; Unstructured in solution	α-helix
1OMR/1JSA	100	97∶109- TNQKLEWAFSLY	3_10_-helix and α-helix in crystal structure; Unstructured in solution	α-helix
1HH5/1F22	100	17∶29 - HKAHAEKLGCDAC	α-helix and 3_10_-helix in crystal structure; α-helix and coil in solution	Coil
		61∶66 - KCGGCH	α-helix in crystal structure; Unstructured in solution	Coil + β-strand
1H0A/1INZ	88.9	3∶15 - TSSLRRQMKNIVH	α-helix in crystal structure; Unstructured in solution	α-helix
5P2P/1SFV	91.9	18∶22 - PLMDF	α-helix in crystal structure; Unstructured in solution	Coil
		113∶115- KEH	3_10_-helix and β-bridge in crystal structure; β-bridge in solution	ND
		120∶123- TKKY	α-helix in crystal structure; Unstructured in solution	ND
**β-class of proteins**				
1OPA/1B4M	100	27∶35 - FATRKIAVR	α-helix in crystal structure; Unstructured in solution	α -helix
		5∶14 - NGTWEMESNE	β-strand in crystal structure; Small β-strand and unstructuredin solution	β-strand
		72∶75 - EHTK	β-strand and turn in crystal structure; unstructured in solution	ND
1XCA/1BLR	99.3	28∶38 - LRKIAVAAASK	α-helix in crystal structure; Unstructured in solution	α -helix
		127∶137- DVVCTRVYVRE	β-strand in crystal structure; Small β-strand and turn in solution	β-strand
		60∶66 - TTEINFK	β-strand in crystal structure, unstructured in solution	β-strand
2GIM/1FA4	99.1	2∶7 - ETYTVKL	β-strand in crystal structure; Unstructured in solution	β-strand
		52∶60 - SADLAKSLS	α-helix in crystal structure; Unstructured in solution	α-helix
		83∶89 - GEYTFYC	β-strand in crystal structure; Unstructured in solution	β-strand
		90∶96 - EPHRGAG	α-helix in crystal structure; Unstructured in solution	Coil
1SPD/1RK7	96.1	41∶48 - GLHGFHVH	β-strand in crystal structure; Unstructured in solution	β-strand
		85∶89 - NVTA	β-strand in crystal structure; Unstructured in solution	ND
		97∶99 - VSI	β-strand in crystal structure; β-strand in solution	ND
		116∶120- TLVVH	β-strand in crystal structure; Unstructured in solution	ND
		54∶60 - TAGCTSA	Turn in crystal structure; Unstructured in solution	Coil
		132∶137- EESTKT	α-helix in crystal structure; Hydrogen bonded in solution	Coil
1J2A/1CLH	99.4	38∶45 - SGFYNNTT	Hydrogen bonded turn and β-sheet; Unstructured in solution	Coil
		48∶57 - RVIPGFMIQG	Anti-parallel β-sheet in crystal structure; Short anti-parallelβ-sheet in solution	Anti-parallel β-sheet
		77∶80 - ADNG	3_10_-helix in crystal structure; Unstructured in solution	ND
1IAZ/1KD6	99.2	8∶15 - VIDGSALS	β-strand and 3_10_-helix in crystal; Hydrogen bonded turnin solution	β-strand + Coil
		129∶138 - DQRMYEELYY	α-helix in crystal structure; Short α-helix followed by unstructuredregion in solution	α-helix
1WHO/1BMW	100	4∶8 - VTFTV	β-strand in crystal structure; Unstructured in solution	ND
		16∶23 - HLAVLVKY	β-strand in crystal structure; Isolated β-bridge mostly unstructuredin solution	β-strand
		28∶34 - MAEVELR	β-strand in crystal structure; Small β-strand mostly unstructuredin solution	β-strand
		51∶55 - VWTFD	β-strand in crystal structure; Unstructured, isolated β-strand,bridge	ND
		64∶70 - FNFRFLT	β-strand in crystal structure; Unstructured, isolated β-bridge	β-strand
		75∶82 - KNVFDDVV	β-strand in crystal structure; Unstructured, isolated β-strand,bridge in solution	β-strand+ Coil
**α + β class of proteins**				
2SAK/1SSN	89	38∶48 - ELLSPHYVEFP	β-strand in X-ray structure; Unstructured in solution	Coil+ β-strand
		76∶81 - FRVVEL	β-strand in crystal structure; Unstructured in solution	β-strand
1QVE/1HPW	96.1	31∶55 - AQLSEAMTLASGLKTKVSDIFSQDG	Two helices connected through a turn in crystal structure;Single helix in solution	α-helix-coil-α-helix
		78∶88 - VAKVTTGGTA	β-strand in crystal structure; Unstructured in solution	β-strand
1C44/1QND	97.6	90∶95 - PQSAFF	α-helix-coil-β-strand in crystal structure; Hydrogen bondedturn in solution	α-helix-coil-β-strand
		99∶102- LKIT	β-strand in crystal structure; Unstructured in solution	ND
		105∶112- MGLAMKLQ	α-helix in crystal structure; Hydrogen bonded turn in solution	α-helix
3IL8/1IKM	100	19∶28 - PKFIKELRVI	3_10_-helix followed by β-strand in crystal structure; β-bridge andβ-strand in solution	Coil + β-strand
		66∶72 - LKRAENS	α-helix in crystal structure; Unstructured in solution	α-helix
1TN3/1RJH	86.1	58∶68 - MKCFLAFTQTK	β-strand in crystal structure; Unstructured in solution	β-strand + Coil
**α/β class of proteins**				
1RRF/1U81	90.6	18∶24 - MRILMVG	β-strand in crystal structure; Unstructured, isolated β-strand,bridge in solution	α-helix + β-strand
		43∶48 - VTTIPT	β-strand in crystal structure; Unstructured, isolated β-bridgein solution	β-strand
		76∶92- PLWRFQNTQGLIFVV	3_10_-helix, unstructured followed by β-strand in crystal structure;α-helical followed by β-strand in solution	α-helix-coil- β-strand
		99∶113- RVNEAREELMRMLAE	α-helix in crystal structure; Unstructured, short helix, turnin solution	α-helix
1EZ9/1EZO	99.7	145∶147- SAL	β-strand in crystal structure; unstructured in solution	ND
		222∶227- TAMTIN	β-strand in crystal structure; unstructured in solution	ND
		258∶266- FVGVLSAGI	β-strand in crystal structure; unstructured in solution	β-strand
		305∶311- KSYEEEL	α-helix in crystal structure; turn and short helix in solution	α-helix
5P21/1CRP	99.4	37∶46 - EDSYRKQVVI 49∶58 - ETCLLDILDT	β-strand-turn- β-strand in crystal structure; Shortened β-strandin solution	β-strand-coil- β-strand
**Small proteins**				
1PSP/1PCP	100	5∶10 - ACRCSR	α-helix in crystal structure; turn in solution	β-strand
		13∶15- PKN	3_10_-helix in crystal structure; unstructured in solution	ND
		55∶59 - SEECV	3_10_-helix in crystal structure; turn in solution	ND
		61∶64 - QVSA	3_10_-helix in crystal structure; turn in solution	ND
1NTN/1W6B	97.3	50∶52 - ESY	Turn in crystal structure; unstructured in solution	ND
		62∶68 - NCNPHPK	Mix of turn in crystal structure; 3_10_-helix in solution	Coil
1BRF/1RWD	94.3	2∶13 - KWVCKICGYIYD	β-strand-turn- β-strand in crystal structure; isolated β-bridgein solution	β-strand
		43∶51 - APKSEFEKL	Mix of β-bridge, 3_10_-helix and β-strand in crystal structure; unstructuredin solution	α-helix
9PTI/1OA5	100	3∶6 - DFCL	3_10_-helix in crystal structure; unstructured in solution	ND
1RDG/1E8J	100	18∶24 - GDPDSGI	Mix of β-bridge, 3_10_-helix and β-bridge in crystal structure; unstructuredin solution	Coil
		30∶33 - FEDL	3_10_-helix in crystal structure; unstructured in solution	ND
		44∶49 - ASKDAF	Mix of β-bridge, 3_10_-helix in crystal structure; unstructuredin solution	ND

ND - Not Determined.

**Table 2:** Structural variations in protein complexes.

*S. No.*	*PDB ID*	*Region of structural variation*	*Structural manifestations* *in the variant region*	*PSIPRED* *Prediction* *for variant* *region*	*Disopred Prediction* *(Residue numbers)*
1	3HRY/3K33	50–73: AALDAEFASLFDTLDSTNKELVNR	α-helix in complex, turns and coil inindividual protein structure	α-helix	72–73
2	3FII/1SFC	27–57: TSNRRLQQTQAQVDEVVDIMRVNVDKVLERD	Largely unstructured in one complexand α-helix in another complex	α-helix	28–36
3	1N7S/1XTG	167–204: MGNEIDTQNRQIDRIMEKADSNKTRIDEANQRATKMLG	α-helix in one complex and largelyunstructured in the other	α-helix	167–169, 171–173, 198, 204
4	3C98/3HD7	189–248: KQALSEIETRHSEIIKLENSIRELHDMFMDMAMLVESQGEMIDRIEYNVEHAVDYVERAV	α-helix in one complex, unstructuredwith distorted helix in another complex	α-helix	189, 248
5	2GRX/1IHR	164–182: PARAQALRIEGQVKVKFDV	α-helix, β-strand in complex,β-strand in individual protein	α-helix, β-strand	164–168
		221–235: GSGIVVNILFKINGT	Coil and β-strand in complex,β-strand in individual protein	β-strand	221, 236
6	2JKR/2BP5	316–325: LAQKIEVRIP	β-strand in one complex, unstructuredin the other	β-strand	316–319
		419–434: IKWVRYIGRSGIYETR	β-strand in one complex, unstructuredin the other	β-strand	431–434
7	1CDJ/1G9M	54–69: RADSRRSLWDQG	α-helix in complex, turn in individualprotein	α-helix	58–59, 62–64
		12–18: VELTCTA	β-strand in individual protein, coilin complex	β-strand	12,18
8	3B2V/1IVO	19–32: FEDHFLSLQRMFNN	α-helix in one complex and unstructuredin other	α-helix	19,32
		92–97: YALAVL	β-strand in one complex and no electrondensity in other	α-helix	–
9	1BGW/2RGR	630–682: LQGNDKDYIDLAFSKKKADDRKEWLRQYEPGTVLDPTLKEIPISDFINKELI	α-helix in complex. Unstructured inindividual protein structure	α-helix	634
10	1K4S/1A36	633–710: QRAPPKTFEKSMMNLQTKIDAKKEQLADARRDLKSAKADAKVMKDAKTKKVVESKKKAVQRLEEQLMKLEVQATDREE	α-helix in one complex and unstructuredin the other	α-helix	633–636,639–640,669–687,700–701,703–710
11	1SER/1SRY	36–86: EVQELKKRLQEVQTERNQVAKRVPKAPPEEKEALIARGKALGEEAKRLEEA	Unstructured in complex and α-helix inindividual structure	Α-helix	48, 50–69
12	1HLO/1R05	12–27: ADKRAHHNALERKRRD	α-helix in the complex (crystal) andunstructured in the individual protein(NMR)	α-helix	27-Dec
13	1B70/1EIY	6–85: LAAIQNARDLEELKALKARYLGKKGLLTQEMKGLSALPLEERRKRGQELNAIKAALEAALEAREKALEEAALKEALERER	α-helix in one complex and unstructuredin the other	α-helix	–
14	1JYE/1EFA	5–13: TLYDVAEYA	α-helix in complex, unstructured inindividual protein	α-helix	11
		16–25: SYQTVSRVVN	α-helix in complex, unstructured inindividual protein	α-helix and β-strand	16–17
		32–46: AKTREKVEAAMAELN	α-helix in complex, unstructured inindividual protein	α-helix	32–37
15	1D1U/1D0E	67–84: SQEARLGIKPHIQRLLDQ	α-helix in one complex and unstructuredin other	α-helix	69–71, 83–84
		99–114: LPVKKPGTNDYRPVQD	α-helix in one complex, β-strand and coilin other	Coil	99–114
		237–273: QQGTRALLQTLGNLGYRASAKKAQICQ	α-helix and coil in one complex, β-strandin the other	α-helix	238–241, 264
16	1VS5/2QOU	19–30: SIVVAIERFVKH	β-strand in one complex, mostlyunstructured in other	β-strand and α-helix	-
		34–46: GKFIKRTTKLHVH	β-strand in one complex, mostlyunstructured in other	β-strand	34–35, 45–46
		57–67: VVEIRECRPLS	β-strand in one complex, mostlyunstructured in other	β-strand	–

**Table 3:** Conformational variation in peptide structures.

*Sl. No*	*Peptide PDB*	*Peptide sequence*	*Structural manifestation*	*PSIPRED result*	*Protein PDB*	*Identity(%)*	*Structural manifestation*
1	1QWP	GSNKGAIIGLM	80% α-helix	63% β- strand	3MOQ(A)	100	63% β-strand
					1HZ3(A)	100	Coil
					2BEG(A)	100	72% β-strand
					1Z0Q(A)	100	45% α-helix
					1IYT(A)	100	α-helix
					2WK3(C)	100	–
					2G47(C)	100	–
					1AML(A)	100	45% α-helix
					1BA4(A)	100	α-helix
					2OTK(C)	100	72% β-strand
2	1CFG	TRYLRIHPQSWVHQIALRMEVL	30% α-helix		3HNB(M)	100	80% β-strand
					3HNY(M)	100	80% β-strand
					3HOB(M)	100	80% β-strand
					1D7P(M)	100	80% β-strand
					3CDZ(B)	100	80% β-strand
					2R7E(B)	100	30% β-strand
3	1P5A	AVGIGALFLGFLGAAGSTMGARSX	25% α-helix	58% α-helix	2ARI(A)	100	α-helix
					3ABI(A)	80	β-strand + α-helix
4	1OMQ	RQIKIWFQNRRMKWKK	70% α-helix	90% β-strand	1HOM(A)	100	80% α-helix
					1AHD(P)	100	α-helix
5	1DVW	TLAVPGMTCAACPITVKK	Coil	66% β-strand	1AFI(A)	100	22% β-strand, 27%α-helix
					1AFJ(A)	100	22% β-strand, 27%α-helix
					2HQI(A)	100	22% β-strand, 27%α-helix
6	1IBN	GLFGAIAGFIENGWEGMIDG	90% α-helix	55% α-helix	3EYM(B)	100	Coil
					1MQL(B)	100	Coil
					1MQN(B)	100	Coil
					5HMG(B)	100	Coil
					3EYK(B)	90	Coil
7	1HZ3	YEVHHQKLVFFAEDVGSNKGAIIGLM	Coil	80% β-strand	2BEG(A)	100	80% β- strand
					1Z0Q(A)	100	80% α-helix
					1IYT(A)	100	a-helix
					2WK3(C)	100	10% β-strand
					3IFN(P)	100	–
					1AML(A)	100	50% α-helix
					1BA4(A)	100	80% α-helix
					2OTK(C)	100	80% β-strand
					1AMB(A)	100	90% α-helix
8	1XV7	FQWQRNIRKVRX	Coil		1LFH(A)	91	α-helix
					1B0L(A)	91	α-helix
9	2BP4	DAEFRHDSGYEVHHQK	70% α-helix	50% β-strand	2WK3	100	Coil
					1BA6	100	Coil
					1IYT	100	50% α-helix
10	2RMV	GNDYEDRYYRENMARYPNQVYYRPVC	Coil	50% α-helix, 15% β-strand	3O79	96	73% α-helix
					1XYW	96	73% α-helix
					2KU4	96	45% α-helix

### Variations between Solution and Crystal Structures

**Figure 2:**
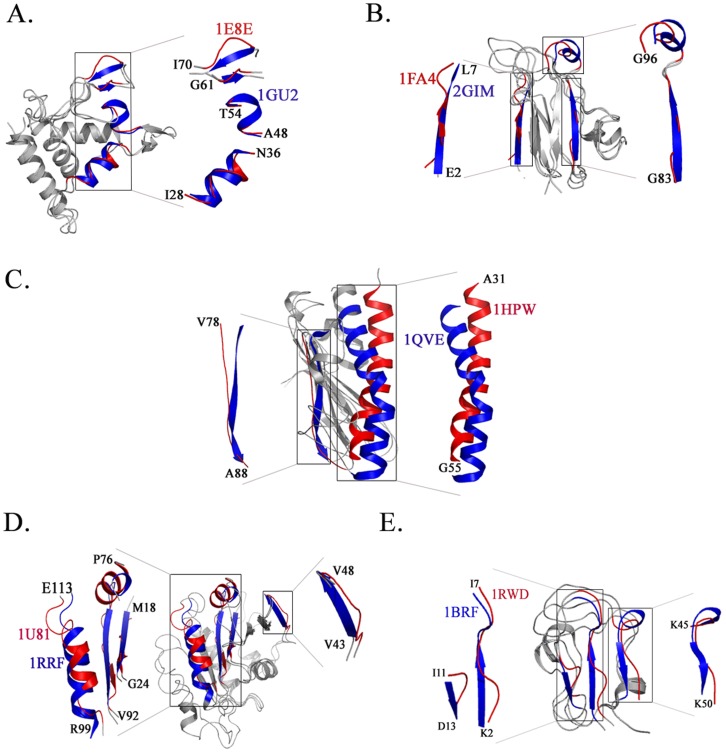
Representative examples of conformational variations. (**A**) All α class (**B**) All β class (**C**) α+β class (**D**) α/β class (**E**) Small proteins. A comprehensive list of these parameters is compiled in Table 1.

A comparison between crystal and NMR structures provides experimental evidence for conformational variation and sampling. In the all α family, most differences, although not all, between the solution and crystal structures could be rationalized to ligand binding. For example, the S100 protein has been structurally characterized in the Ca^2+^- free form (PDB: 1K9P), the Ca^2+^-bound form (PDB: 1K96) [4] and in solution (PDB: 1A03) [5]. In the X-ray structure, the stretch proximal to the ligand binding site adopts a helical conformation in the crystal structure whereas it is unstructured in the NMR structure despite the presence of a bound Ca^2+^ cofactor. Another example of conformational change induced by ligand binding are the crystal (PDB: 1GU2) and solution structures (PDB: 1E8E) of the oxidized form of Cytochrome C that reveal structural differences closer to the heme binding pocket [6], [7]. These include a stretch I28–N36 (ITDGKIFFN) that adopts a helical conformation in the crystal structure while it is unstructured in solution. The segments A48–T54 (ACASCHT) and G61–I70 (GKNIVTGKEI) adopt α-helical and β-sheet conformation in the crystal structure as opposed to hydrogen bonded turns in solution. These structural variations are highlighted in Figure 2A.

Plastocyanins are a good example of structural differences in the β-class of proteins. The X-ray (2GIM) [8] and solution structures (1FA4) [9] of *Anabaena variabilis* plastocyanin differ in their secondary structural content (Figure 2B). β-strands are less structured in solution compared to crystal structures where they form extended β strands. Also, residues S52–S60 (SADLAKSLS) and E90–G96 (EPHRGAG) in the crystal structure from *A. variabilis* plastocyanin and the corresponding region in the *Phormidium laminosum* homologue (PDB: 2Q5B) are α-helical in the crystal structure while they remain unstructured in solution.

Three pilin crystal structures (α + β family in SCOP) exemplify variations in this structural class. The structural descriptions include *N. gonorrhoeae* strain MS11 pilin [10], the truncated toxin-coregulated pilin from *V. cholerae* [11] the *P. aeruginosa* strain K pilin [12] and the ΔK122–4 pilin examined by NMR [13]. The ΔK122–4 crystal structure (PDB: 1QVE) exhibits a characteristic type IVa pilin fold, with the N-terminal α-helix (α1–C) packed onto a four-stranded antiparallel β-sheet. Although the relative positions of the core secondary structure elements are well-conserved among the crystal structures, they differ considerably between the crystal and NMR structure of ΔK122–4 pilin (PDB: 1HPW). Superposition of these structures shows that in the solution structure of ΔK122–4, the N-terminal α-helix A31–G55 (AQLSEAMTLASGLKTKVSDIFSQDG) is shifted by one turn and thus deflected away from the β-sheet [12]. The C-terminal residues V78–A88 (VAKVTTGGTA) form a β-strand in the crystal structure whereas they are unstructured in solution (Figure 2C).

ADP-ribosylation factors (ARF-1) belong to the α/β family of proteins. Structural comparison in this case was made using four structural models viz., the GDP bound structure of human ARF-1 (1HUR), rat ARF-1 (1RRF) and human ARF-1 (1U81) [14]. A comparison between the crystal and solution structures reveals several changes. The region P76–N84 (PLWRHYFQN) is helical in solution NMR (1U81) but unstructured in the crystal structure. Other differences include regions M18–M22 (MRILM), V43–V53 (VTTIPTIGFNV) and T85–V92 (TQGLIFVV) which are β-strands in the crystal structures of these ARFs but are unstructured or adopt turns/bridges in solution. Similarly, R99–E113 (RVNEAREELMRMLAE) is a well defined α-helical stretch present in the crystal structure while in solution this stretch is a mix of a hydrogen bonded turn (R99–E102), a short helix (E102–L107) followed by another hydrogen bonded turn (M108–E113; Figure 2D). Another prominent example is that of Rubredoxin where the major difference between the X-Ray (PDB: 1BRF) [15] and NMR structure (PDB: 1RWD) is the absence of β-strands in solution (Figure 2E).

### Structural Variation Due to Conformational Restraints in a Larger Macromolecular Complex

**Figure 3:**
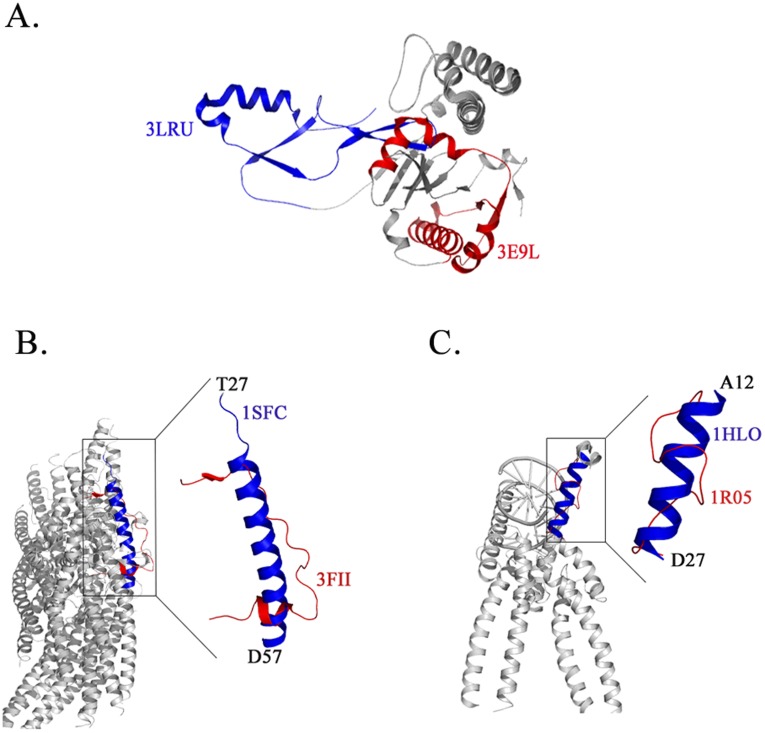
Conformational variations induced by interactions with proteins and nucleic acids. Structural differences in (**A**) human splicing protein Prp-8 (Full length and N-terminal deletion) variants. These structures illustrate sequence length-dependent structural changes. (**B**) & (**C**) depict structural changes in protein-protein and protein-nucleic acid complexes.

An experimental construct that allows a recombinant protein to be purified in large amounts to homogeneity is a critical step towards structure determination. Important variables in this step include the length of the recombinant protein along with the choice of an affinity or solubilization tag. A particularly dramatic case of a change in the fold of a protein due to a change in the sequence-length is that of human PRP-8 D4 structure that has a different fold from that determined for a shorter D4 construct (Figure 3A). In the case of multi-protein complexes, co-expression and co-purification of interacting proteins often provides a viable route towards structural characterization. Protein-protein interactions often involve conformational changes that make the complex more stable and tractable for crystallization. These conformational changes can also be context-dependent. An example of this feature is Synaptobrevin, a part of the vesicle-associated membrane protein (VAMP) family that forms a component of the neuronal SNARE (soluble *N*-ethylmaleimide-sensitive factor attachment receptor) complex. The isolated solution structure of synaptobrevin is largely unfolded but is a well-defined helix in the SNARE complex [16]. The structure of synaptobrevin (residues 27–57) in complex with Neurotoxin type F from *Clostridium botulinum* (3FII) [17] shows a largely disordered segment with a small β-strand at the N terminus and a small α-helix at the C terminal end while the same segment is a helix in the neuronal synaptic fusion complex (PDB: 1SFC) [18]. A superposition of the two structures is shown in Figure 3B. A search for similar stretches in the PDB yielded several protein-complexes in which this sequence-stretch is an ordered α-helix. For example, synaptobrevin in the complexin-SNARE complex (PDB: 1KIL) [19] shows a well defined α-helix similar to other SNARE complexes (PDB: 1N7S, 3HD7, 3IPD) [20]. Recombinant proteins of different sizes (based on different expression constructs) also influence secondary structural composition. For example, in the case of the catalytic domains of Protein Tyrosine Phosphatases (PTP), addition of an additional stretch of ca 45 residues substantially influences the solubility and propensity to crystallize. This stretch either adopts an α helical conformation or is involved in dimerization [21]. Context-dependent conformational changes are more common in protein-nucleic acid complexes (Figure 3C). Indeed, successful structure determination of protein-nucleic acid complexes is often only possible in the presence of the interacting components (Table 2).

### Peptide Structures Exemplify Conformational Selection

**Figure 4:**
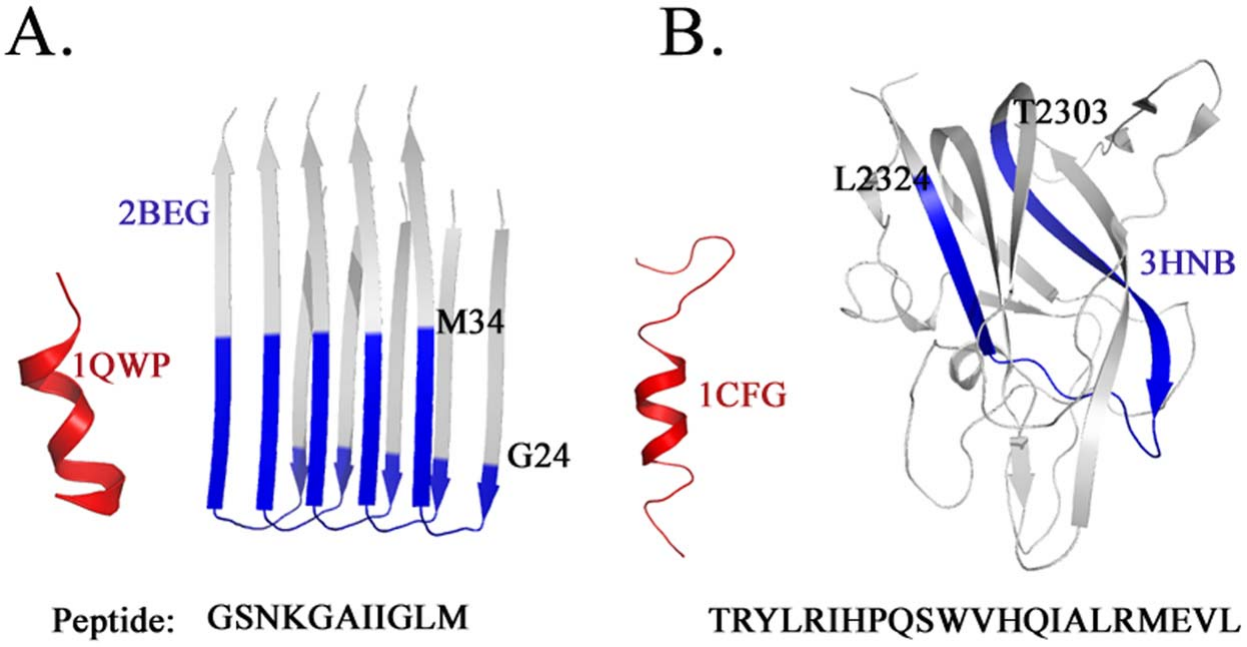
Structural variability in peptide sequences. (**A**) Context dependent conformational changes of a peptide from the amyloid β A4 protein (PDB: 1QWP) and (**B**) C2 domain of Factor VIII (PDB: 1CFG).

Structural differences in peptide structures have been extensively examined in the case of the amyloid peptides and chameleon sequences [22], [23]. For instance, the NMR structure of an eleven residue peptide from the amyloid β A4 protein (PDB: 1QWP) adopts a α-helical conformation. The same sequence, however, variously adopts β-strand conformations (PDB: 3MOQ, 2BEG, 2OTK) [24], [25] α-helical segments (PDB: 1Z0Q, 1IYT, 1BA4, 1AML) [26] or coiled-coil conformations (PDB: 1HZ3) as a part of a larger protein sequence (Figure 4A; Figure S1). Another representative example is the NMR structure of a peptide from the C2 domain of Factor VIII (PDB: 1CFG) [27] which is α-helical in isolation. The same sequence in the context of the entire C2 domain of Factor VIII (PDB: 3HNB, 3HNY, 3HOB, 1D7P, 3CDZ, 1IQD) [28], [29], [30] adopts a β-strand conformation (Figure 4B). It is relevant to note in this context that the secondary structure prediction (using PSIPRED) [31] for this peptide revealed a 22% β-strand and 63% α-helical structure.

### Limitations of Temperature Factor and CONCOORD Simulations to Examine Conformational Variation

**Figure 5:**
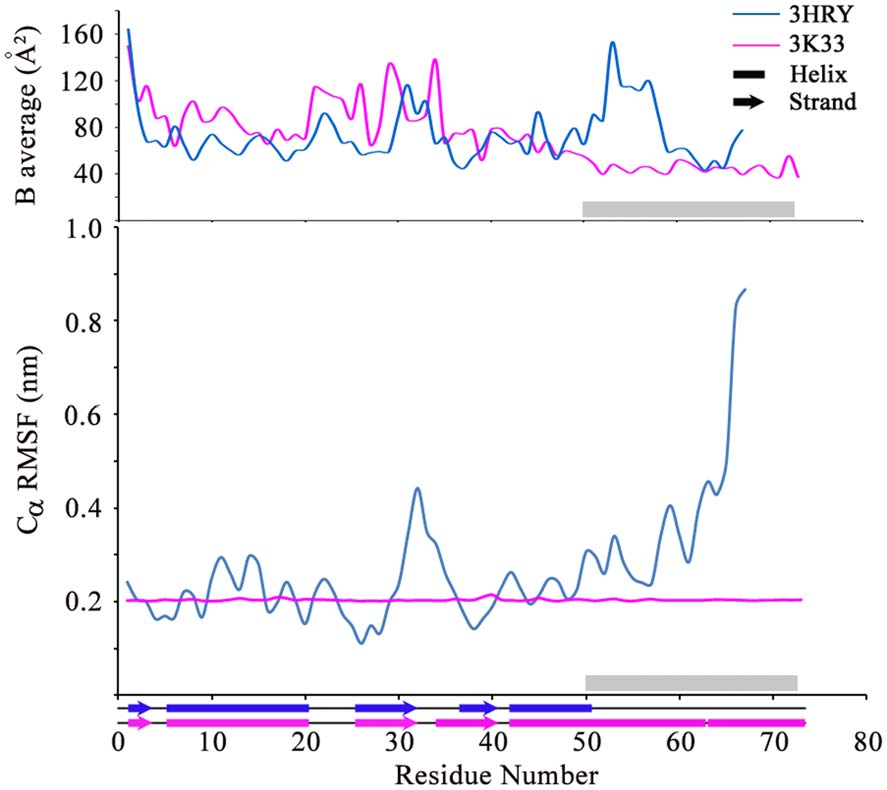
*In silico* methods to extract dynamic information. CONCOORD and temperature factor analysis of Prevent host death protein (Phd: 3HRY) that shows a disordered-to-ordered conformational transition upon forming a complex with the Death on curing protein (Phd-Doc complex: 3K33). The grey bar represents the region in the Phd protein that undergoes structural change upon forming the Phd-Doc complex.

High B-factors, classical indicators for conformational variation or flexibility, are often ambiguous due to experimental limitations. A case for this observation is Synaptobrevin, a protein involved in two different complexes, one with Botulinum Neurotoxin (PDB: 3FII) and the other with SNARE complex proteins (PDB: 1SFC). In this case, the unstructured component (PDB: 3FII) showed slightly lower B-factor values as compared to the structured component (PDB: 1SFC). We stress here, however, that a vast majority of segments that show conformational variability in this dataset can be clearly flagged by virtue of high B factors in those stretches when compared with the rest of the protein. In these cases, alternate conformations are also easily identifiable by *in silico* methods. For example, in the Prevent-host-death (Phd) protein, the region 50–73 forms an α-helix when involved in a complex with the Death-on-curing (Doc) protein (PDB: 3K33) while it remains unstructured in isolation (3HRY). The temperature factors show a marked increase for 3HRY while in 3K33, where the protein is structured, the region has a B-factor that is below the average value for the protein. Consistent with this experimental data, this stretch in 3HRY shows high RMS fluctuation in a CONCOORD analysis that correlates well with changes in secondary structure conformations. The Dictionary of Secondary Structure Predictions (DSSP) output for the stretch in 3HRY shows a largely turn-dominated profile interspersed with 3_10_-helices, bends and alpha helices at several points of time in the simulation (Figure 5).

### Comparison Between the Secondary Structure Propensity and Conformational Variations

The secondary structure propensity is highlighted in several cases of conformational differences between solution and crystal structure. For example, in the crystal structure (PDB: 1NZN) of the cytosolic domain of human mitochondrial fission protein Fis1, the region E5–S13 (EAVLNELVSVED) is α-helical whereas it is unstructured in solution (PDB: 1PC2). The PSIPRED prediction for this stretch is a α-helix. These results from the comparative analysis dataset of X-ray and NMR pairs are summarized in Table 1. A comprehensive list of root-mean-square-deviations (RMSD) for this dataset is compiled in Table S2. This aspect of conformational selection is also seen in the case of multi-protein complexes. In the synaptosomal associated protein complexed with Botulinum Neurotoxin BONT/A (PDB: 1XTG), the region M167–G204 is unstructured. In the truncated neuronal SNARE complex (PDB: 1N7S), however, the stretch is helical, consistent with the secondary structure prediction. A summary of these observations, along with the output obtained from the DISOPRED [32] predictions is compiled in Table 2.

### Effect of Experimental Conditions on Conformational Differences

The composition of a crystallization condition can influence the secondary structural composition of a protein and hence facilitate conformational selection. This analysis is compiled in Tables S3 and S4. The compilation in Table S3 suggests that polyethylene glycols (PEG; in the molecular range of 200–4000) are involved in the crystallization of ca 80% of the proteins in this dataset while a minority (ca 10%) of them have salts like ammonium sulphate. PEGs serve to aggregate protein molecules, often inducing secondary structural features, thus increasing the chance of crystallization [33]. This observation perhaps rationalizes the finding that in the dataset of structural pairs (X-ray and NMR; Table S3), most of the crystal structures showed additional secondary structural elements than the corresponding solution structures. While an ideal comparison would have involved a pair of structural models (X-Ray/NMR) where the structure determination was performed under identical conditions, these are difficult to achieve due to divergent experimental requirements of mono-disperse solution behavior of a protein sample for NMR versus conditions that promote systematic aggregation to form crystals. Conformational selection, in the case of multi-protein complexes is also facilitated by crystallization agents. For example, the crystallization condition of the Prevent host death protein (3HRY) where the stretch 50–73 is unstructured contains Ethylene glycol and PEG 8000 as precipitants. Ethylene glycol is known to decrease α-helicity and its interaction with proteins is enhanced in the presence of high molecular weight PEG [34]. Hydrophobic interactions are known to increase with high salt concentrations [35]. These interactions could have facilitated the folding of the stretch (L630–E710) in DNA Topoisomerase 2 (PDB: 2RGR) as the salt concentrations are much higher than the corresponding concentration in the structure without bound DNA (PDB: 1BGW). Perhaps coincidentally, an observation on the denaturation of β sheets at low pH [36] also correlates with the structure of the T-cell surface glycoprotein CD4 (PDB: 1CDJ, 1G9M) which shows well-defined β-strands when compared to its structure in complex with two other proteins where it is unstructured. Representative cases of conformational changes induced by crystal packing effects are illustrated in Figure S1. It is, however, difficult to correlate crystallization conditions or the high protein concentration in an NMR experiment with the packing in a protein structure. This analysis is summarized in Table S5.

The packing fraction varies in the range of 0.66 to 0.84 [37]. The average packing density of proteins is about 0.75. Comparative studies of packing density and cavity analysis of similar NMR and crystal structures for all classes of proteins was performed using Voronoia [38]. The grid level for all the input PDBs were adjusted to 0.2 for calculating the parameters. This analysis, however, did not yield new information, apart from confirming that NMR structures tend to have a slightly higher packing density when compared to crystal structures.

## Discussion

**Figure 6:**
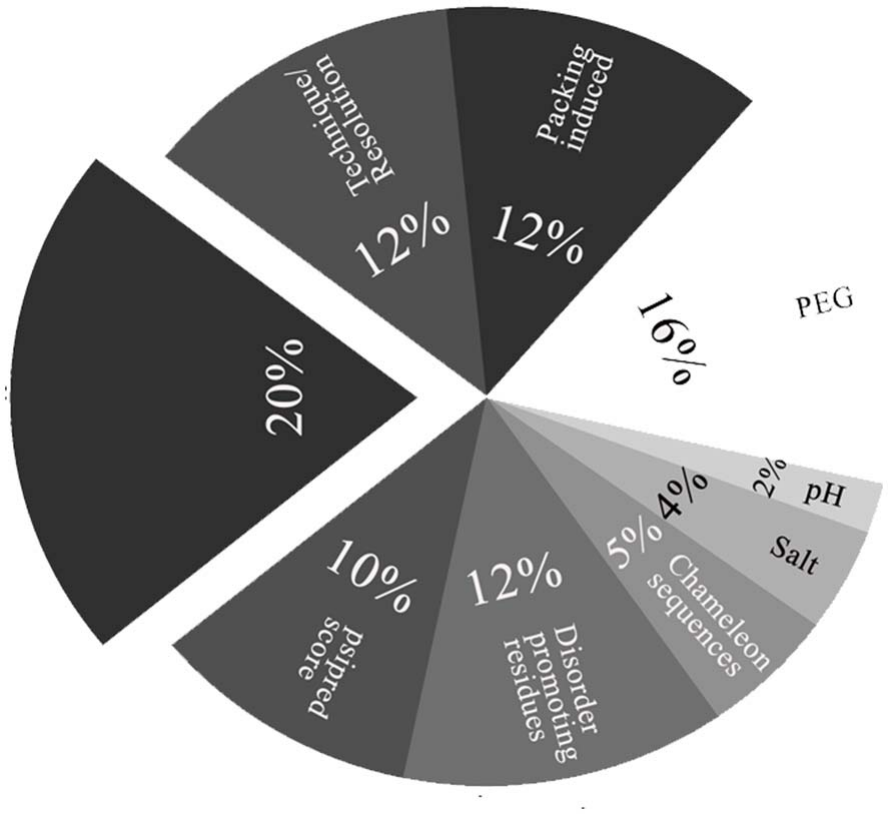
Summary of the potential cause of variations in protein structural models. This data is based on information presented in Tables 1–3. The abbreviations used here are- psipred score: differences between predicted and observed secondary structure; Disorder promoting residues, Chameleon sequences: Classification based on aminoacid composition; Salt, pH, PEG: Effects of ionic strength, pH, high concentration of polyethylene glycol; Packing induced, Technique/Resolution: Differences between solution and crystal structural models.

Conformational changes in proteins often provide the first step to rationalize a functional role or to build a mechanistic hypothesis for a biological observation. Deducing conformational variations is thus an important step in functional annotation. This information is also crucial for structural models that form the basis for *in silico* modeling of homologous proteins or as fragments that are utilized for *de novo* structural prediction. An understated feature of currently available structural models is that they implicitly incorporate experimental conditions, limitations inherent to the method for structure determination and data as well as by the length of the recombinant protein construct. These limitations, in an extreme case, provide alternate structural models for an identical protein sequence. This was noted, most recently, in the case of the human PRP-8 D4 structure that has a different fold than that determined for a shorter D4 construct (Figure 3A) [39]. In this study, we examined representative structural models in the PDB for evidence of conformational selection or context-dependent modeling [40], [41]. The dataset for this analysis was spread across different structural families and multi-component (protein-protein and protein-nucleic acid) complexes. This diverse set of protein structures was evaluated for sequence features (secondary structure propensity, disorder) that could suggest alternate conformations. In particular, aspects such as a skewed distribution of highly fluctuating residues (G, A, S, P, D) over weakly fluctuating residues (I, L, M, Y, F, W, H) in irregular structural elements (loops), chameleon sequences and intrinsically disordered proteins [42], [43] were examined. The next step involved an examination of context dependent structural variations that could be ascribed to experimental conditions, packing, or induction of secondary structure by binding to cognate partners. The result of this analysis is compiled in Figure 6 and Figure S1. This analysis suggests that methods to de-convolute dynamic information are better served by incorporating both sequence features (for example, disorder propensity, ambivalent secondary structures and chameleonic sequences) and experimental conditions that nucleate or aid conformational selection.

Static structural models, such as those obtained from single crystal X-Ray diffraction methods, incorporate dynamic information at multiple layers. B-factors and ligand induced displacements provide an insight into potential conformational changes and conformational sampling. The so-called consensus structures that involve different levels of structural overlap in multiple crystal structures have been proposed as a route to obtain dynamic information that is otherwise not evident from single crystal structural models. An alternative approach involves diffuse scattering that originates from fluctuations in the average electron density and appears as a background on an X-ray film. This analysis, however, requires ultra high resolution structures as the higher order scattering makes a significant contribution at high resolutions. Furthermore, these studies also require robust scaling between the vibrational density of states to make a comparison between experimental and theoretical temperature factors. The data-set utilized in this manuscript was compiled with the aim of having protein structural models determined using different experimental methods. This data-set does not contain crystal structures of the resolution required to analyze diffuse scattering. In an effort to examine if potential conformational variants could be deduced from a given crystal structure, we performed an analysis using CONCOORD [44]. A significant number of outliers, however, suggest that both normal modes and CONCOORD analysis, the preferred route to examine structural variations in the absence of detailed MD simulations, are inadequate (Figure 5). Do conformational differences actually depict characteristics similar to those of the so-called chameleon sequences? The sequence analyses presented in Table 3 broadly support that perspective. The sequence composition also suggests more scope for residue fluctuations [45] supporting the view that structural models represent conformational selection influenced by experimental conditions.

Put together, this analysis suggests that experimental conditions substantially influence conformational selection. The experimentally determined structural model, that is the template for *in silico* methods to derive dynamic information, can thus bias interpretations on conformational variation and dynamics. This study presents a case for a more comprehensive inclusion of physico-chemical parameters associated with experimental conditions in the interpretation of protein structural data. This analysis also emphasizes the need to incorporate information on chameleon sequences in protein structural models while inferring dynamic properties of proteins.

## Methods

### Dataset of Structures Used in this Analysis

A compilation of protein structures was initially based on the SCOP (1.73 version) database. Upon the identification of candidate structural models, an advanced search in PDB was performed to obtain the corresponding protein structure determined either in solution by NMR or as a part of a larger macromolecular complex. The following criteria were used to obtain the dataset for this analysis- i. Resolution cut-off for the X-ray crystal structures was set at 3.00 Å (3.9 Å in complexes) and ii. Only structures with a minimum overall sequence identity of 30% in a pair-wise alignment were selected. For this purpose, the EMBOSS Align program was used. PyMOL was used for the superposition of the structure pairs. The dataset of protein structural pairs had a total of 31 pairs of structures, belonging to five SCOP classes. The dataset for disordered proteins was collated from DISPROT [3]. The homologues for the disordered proteins for which PDB files were available were compiled from the PDB. The dataset for peptide structures were obtained from the PRF database within the DBGET integrated database retrieval system. In this search, the peptide length was limited to 10–40 amino acids. 110 peptide structures that contained only naturally-occurring amino acids were chosen for the study. Based on the availability of comparable sequences within large protein structures, a dataset of 45 peptide structures were compiled.

### RMSD Calculation, Temperature Factor and Normal Mode Analysis

The root mean square deviation (RMSD) was calculated between one X-ray crystallographic structure and the average structure from the NMR ensemble using LSQMAN [46]. The average of that RMSD was taken for further analysis as the deviation between the two representative proteins. The ensemble average for the NMR structure was calculated using MOLMOL [47]. The B-factor analysis was also performed on all the X-ray structures in the database presented in this work. Packing densities and cavities of the protein molecules for each structure in the dataset were calculated using *Voronoia* [38]. In this method, packing density is defined by the equation: PD  =  V_vdw_/(V_vdw_+ V_se_) where V_vdw_ is the assigned atomic volume inside the atoms’ Van der Waals radius and V_se_ is the remaining solvent excluded volume. Only monomers of each structure were used for calculating the packing parameters while an averaged structure was used for calculating values in the case of solution NMR. A grid level of 0.2 was assigned for calculating the packing densities and cavity in each structure. Water molecules were removed from the coordinate files and only monomer structures were considered for calculations.

### Analysis of Conformational Dynamics

Along with the crystal structures, we also used CONCOORD (from CONstraints to COORDinates) tool [44] to predict and analyze the likely motion(s) of the segments/motifs in proteins in our dataset. All the simulations were performed for 1000 ps using the default parameters to generate 1000 conformations. The trajectory analysis of the region of differences during the course of simulations was performed using the RMSF (root mean square fluctuation) plots of the residues during the simulation period. Changes in secondary structure were analyzed using DSSP [48].

### Sequence Analysis of the Regions of Conformational Change

The peptide segments that show conformational differences between X-Ray and NMR structures as well as protein complexes were used as a template to search for similar sequences using BLAST (Basic Local Alignment Search Tool) [49]. Cut-off values for sequence identity were set at 80% with the template segment. The secondary structure propensities of the protein sequences in this dataset were determined using PSIPRED [31]. In case of disordered proteins, sequence analysis were performed both using PSIPRED and DISOPRED [32].

## Supporting Information

Figure S1 **Representative examples of structural variations in protein models that can be rationalized by oligomerization or crystal packing (A)** Human mitochondrial Fis1∶1NZN/1PC2 **(B)** Interleukin 8∶3IL8/1IKM **(C)** Pancreatic spasmolytic peptide: 1PSP/1PCP **(D)** Sterol carrier protein-2∶1C44/1QND (E) The allergen PHL P2∶1WHO/1BMW.
(PDF)

Table S1 **Comparison between X-ray and NMR structures in different classes of proteins.**
(DOC)

Table S2 **Comparison of root mean square deviations (r.m.s.d.) between the NMR ensemble and crystal structures.**
(XLS)

Table S3 **Analysis of experimental conditions for X-ray and NMR pairs.**
(XLS)

Table S4 **Analysis of experimental conditions in multi-protein complexes.**
(XLS**)**

Table S5 **Packing analysis of X-ray and NMR ensembles.**
(XLS)
