# Supplementary material for: Analysis of Conformational Variation in Macromolecular Structural Models
Source: PLoS One. 2012 Jul 9;7(7):e39993. doi: 10.1371/journal.pone.0039993 (PMC3392262; doi:10.1371/journal.pone.0039993)

Supplementary Figure 1. Representative examples of structural variations in protein models that can be rationalized by oligomerization or crystal packing

A. Human mitochondrial Fis1: 1NZN/1PC2

Region of variation 5:13 (sequence- EAVLNELVSVED)

Human mitochondrial Fis1 (1NZN) is a dimer in the crystal structure (Dohm *et al*., Proteins, 2004). Fis1 is composed of six helices (helices α2, α4 and α6 are arranged to form an amphiphilic, concave surface). The crystal packing of Fis1 reveals one arrangement by which the concave surface mediates binding. In the crystal, a dimer of Fis1 was observed in which the concave surface of a Fis1 molecule makes contacts with helix α1 of a symmetry related molecule. Fis1 is a monomer in solution- helix α1 is flexible in this case (Suzuki *et al*, J Mol Biol, 2003).

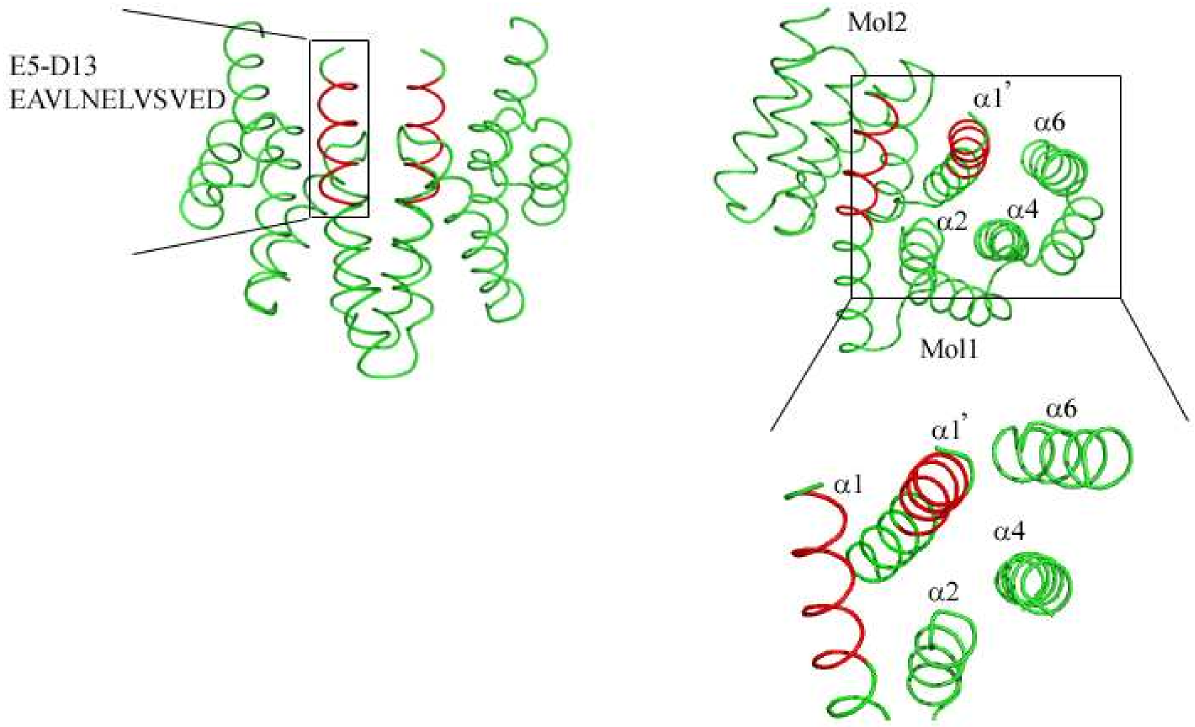

B. Interleukin 8: 3IL8/1IKM

region of variation 19:28 (sequence- PKFIKELRVI)

66:72 (sequence- LKRAENS)

Interleukin 8 (IL-8) adopts a dimeric arrangement in the crystal, in which residues 23-29 and 55-62 play an important role in packing monomeric units. The solution structure of IL-8 is a monomer. Residues 23-28 make tight hydrogen bonding interactions to form an extended β-sheet comprising of six strands and two helices (residues 55-72) which are unstructured in

solution but pack at the dimer interface and are structured in the crystal (Baldwin et al, PNAS, 1991; Rajarathnam et al, Biochemistry, 1995).

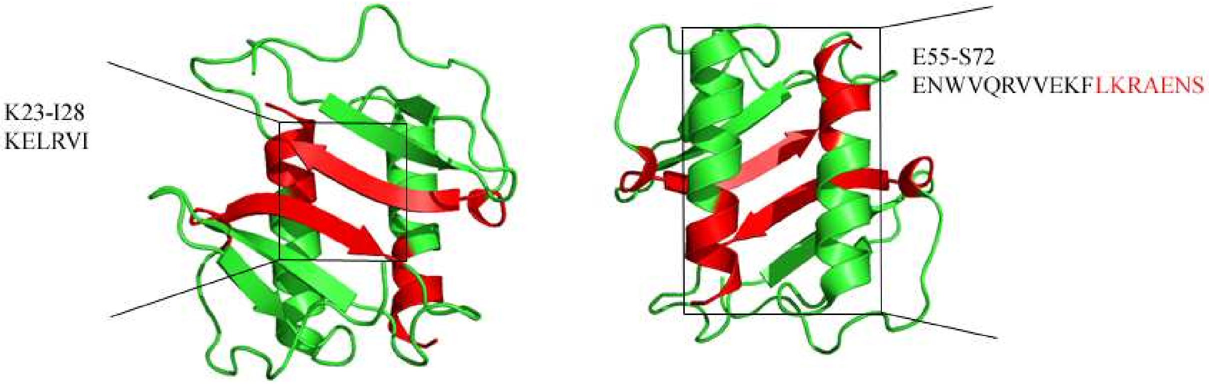

C. Pancreatic spasmolytic peptide: 1PSP/1PCP

In the case of the pancreatic spasmolytic peptide, a member of mammalian trefoil family of peptides, conformational variations are located away from the interface.

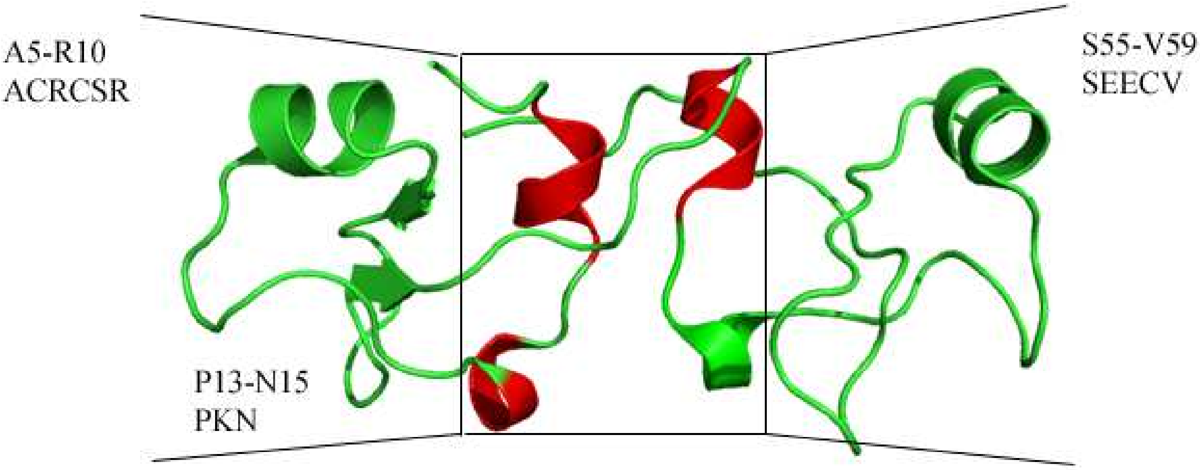

D. Sterol carrier protein-2: 1C44/1QND

In the sterol carrier protein-2, the conformationally variable region (red) interacts with a symmetry related molecule.

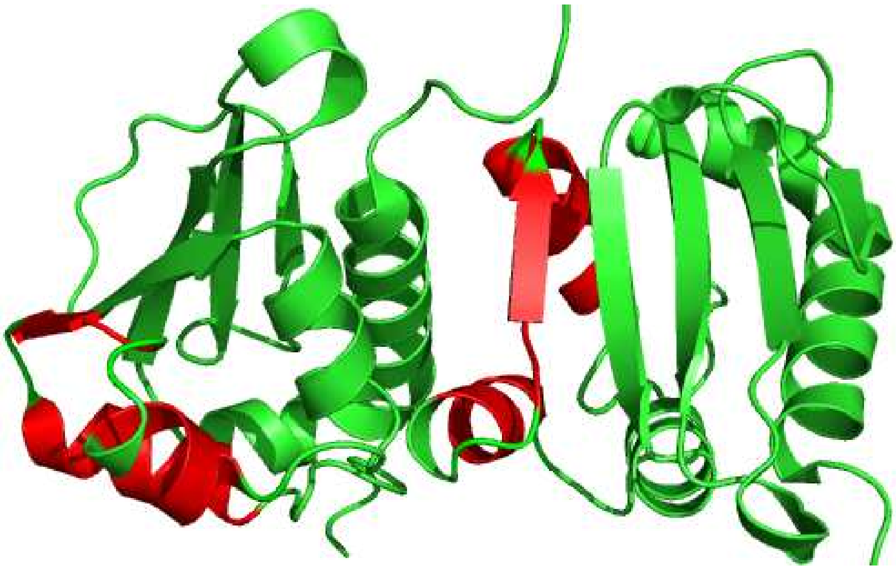

E. The allergen PHL P2: 1WHO/1BMW

In the allergen PHL P2, crystal packing effects can be seen to influence conformational stability.

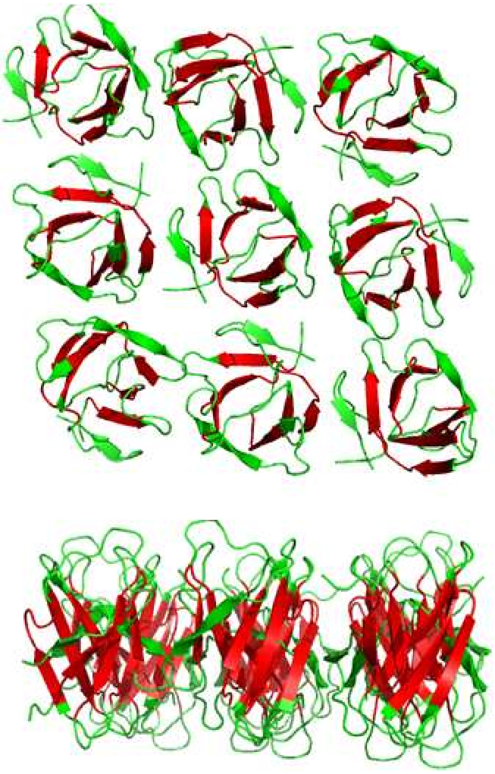

Supplement: Figure S1 — Representative examples of structural variations in protein models that can be rationalized by oligomerization or crystal packing (A) Human mitochondrial Fis1∶1NZN/1PC2 (B) Interleukin 8∶3IL8/1IKM (C) Pancreatic spasmolytic peptide: 1PSP/1PCP (D) Sterol carrier protein-2∶1C44/1QND (E) The allergen PHL P2∶1WHO/1BMW. (PDF) [file pone.0039993.s001.pdf]
