# Supplementary material for: Analysis of Conformational Variation in Macromolecular Structural Models
Source: PLoS One. 2012 Jul 9;7(7):e39993. doi: 10.1371/journal.pone.0039993 (PMC3392262; doi:10.1371/journal.pone.0039993)
Supplement: Table S1 — Comparison between X-ray and NMR structures in different classes of proteins. (DOC) [file pone.0039993.s002.doc]

**Table S1: *Comparison between X-ray and NMR structures in different classes of proteins***

**α-class of proteins**

| ***X-ray PDB*** | ***NMR PDB*** | ***Region of Structural Variation*** | ***Link to TASSER results*** |
| --- | --- | --- | --- |
| **1K96** | 1A03 | 42: 51 - LTIGSKLQDA | <http://zhanglab.ccmb.med.umich.edu/I-TASSER/output/S85146/> |
| **1GU2** | 1E8E | 28: 36 - ITDGKIFFN  48: 54 - ACASCHT  61: 70 - GKNIVTGKEI | <http://zhanglab.ccmb.med.umich.edu/I-TASSER/output/S89012> |
| **1NZN** | 1PC2 | 5: 13 - EAVLNELVSVED | <http://zhanglab.ccmb.med.umich.edu/I-TASSER/output/S85147/> |
| **1OMR** | 1JSA | 97:109- TNQKLEWAFSLY | <http://zhanglab.ccmb.med.umich.edu/I-TASSER/output/S88727/> |
| **1I27** | 1NHA | 478: 483- QTKKTGL | <http://zhanglab.ccmb.med.umich.edu/I-TASSER/output/S89013> |
| **1HH5** | 1F22 | 17: 29 - HKAHAEKLGCDAC  61: 66 - KCGGCH | <http://zhanglab.ccmb.med.umich.edu/I-TASSER/output/S88803/> |
| **1H0A** | 1INZ | 3: 15 - TSSLRRQMKNIVH | <http://zhanglab.ccmb.med.umich.edu/I-TASSER/output/S89014/> |
| **5P2P** | 1SFV | 18: 22 - PLMDF  113: 115 - KEH  120: 123 - TKKY | <http://zhanglab.ccmb.med.umich.edu/I-TASSER/output/S85966/> |

**>1K96**

MACPLDQAIGLLVAIFHKYSGREGDKHTLSKKELKELIQKELTIGSKLQDAEIARLMEDLDRNKDQEVNFQEYVTFLGALALIYNEALKG

**Similar protein structures from the Protein Data Bank:** [**94**](http://www.sbkb.org/kb/report.jsp?sid=s1323076118-53&pid=structures)

**Proteins with highly similar structures in PDB**

(<http://zhanglab.ccmb.med.umich.edu/I-TASSER/output/S85146/>)

**id = 1odb_F I = 42% E = 3.5E-09 TM_Score : 0.862 RMSD 1.66**
1K96 : 2 ACPLDQAIGLLVAIFHKYSGREGDKHTLSKKELKELIQKEL--TIGSKLQDAEIARLMED
 + L++ + +V IFH+YS R+G TLSK ELK+L+ KEL TI + A I + +
1ODB : 4 STKLEEHLEGIVNIFHQYSVRKGHFDTLSKGELKQLLTKELANTIKNIKDKAVIDEIFQG

1K96 : LDRNKDQEVNFQEYVTFLGALAL 82
 LD N+D++V+FQE+++ + A+AL
1ODB : LDANQDEQVDFQEFISLV-AIAL 85

**id = 1xk4_L I = 32% E = 1.4E-07 TM_Score : 0.857 RMSD 1.48**
1K96 : 5 LDQAIGLLVAIFHKYSGREGDKHTLSKKELKELIQKELTIGSKLQDAE---IARLMEDLD
 L++ I ++ FH+YS + G TL++ E KEL++K+L K ++ I +MEDLD
1XK4 : 7 LERNIETIINTFHQYSVKLGHPDTLNQGEFKELVRKDLQNFLKKENKNEKVIEHIMEDLD

1K96 : RNKDQEVNFQEYVTFLGALALIYNEAL 88
 N D++++F+E++ + L +E +
1XK4 : TNADKQLSFEEFIMLMARLTWASHEKM 93

**id = 1qls_A I = 30% E = 5.5E-07 TM_Score : 0.834 RMSD 1.6**
1K96 : 6 DQAIGLLVAIFHKYSGREGDKHTLSKKELKELIQKELTIGSKLQ--DAEIARLMEDLDRN
 ++ I L+AIF K++GR+G+ +SK E + EL ++ Q + R+M+ LD +
1QLS : 9 ERCIESLIAIFQKHAGRDGNNTKISKTEFLIFMNTELAAFTQNQKDPGVLDRMMKKLDLD

1K96 : KDQEVNFQEYVTFLGALALIYNEAL 88
 D +++FQE++ +G LA+ +++
1QLS : SDGQLDFQEFLNLIGGLAIACHDSF 93

**id = 2y5i_F I = 45% E = 3.5E-15 TM_Score : 0.824 RMSD 1.87**
1K96 : 1 MACPLDQAIGLLVAIFHKYSGREGDKHTLSKKELKELIQKELT--IGSKLQDAEIARLME
 M L+ A+ L+ +FH YSG EGDK+ LSK ELKEL+ ELT + S+ + ++M
2Y5I : 1 MPSKLEGAMDALITVFHNYSGSEGDKYKLSKGELKELLNAELTDFLMSQKDPMLVEKIMN

1K96 : DLDRNKDQEVNFQEYVTFLGALALIYNE 86
 DLD NKD EV+F E+V + AL + N+
2Y5I : DLDSNKDNEVDFNEFVVLVAALTVACND 88

**id = 1a4p_B I = 29% E = 3.0E-05 TM_Score : 0.810 RMSD 1.70**
1K96 : 5 LDQAIGLLVAIFHKYSGREGDKHTLSKKELKELIQKELTIGSKLQDAEIA--RLMEDLDR
 ++ A+ ++ FHK++G DK L+K++L+ L++KE + Q +A ++M+DLD+
1A4P : 4 MEHAMETMMFTFHKFAG---DKGYLTKEDLRVLMEKEFPGFLENQKDPLAVDKIMKDLDQ

1K96 : NKDQEVNFQEYVTFLGALALIYNE 86
 +D +V FQ + + + L + N+
1A4P : CRDGKVGFQSFFSLIAGLTIACND 84

**id = 3n22_B I = 46% E = 1.4E-18 TM_Score : 0.800 RMSD 1.86**
1K96 : 1 MACPLDQAIGLLVAIFHKYSGREGDKHTLSKKELKELIQKELT--IGSKLQDAEIARLME
 M+ L+QA+ +LV FHKYS +EGDK LSK E+KEL+ KEL +G K+ + + +LM
3N22 : 2 MSSSLEQALAVLVTTFHKYSSQEGDKFKLSKGEMKELLHKELPSFVGEKVDEEGLKKLMG

1K96 : DLDRNKDQEVNFQEYVTFLGALALIYNEALKG 90
 LD N DQ+V+FQEY FL + ++ N+ +G
3N22 : SLDENSDQQVDFQEYAVFLALITVMSNDFFQG 93

**id = 1a03_B I = 90% E = 5.4E-43**
1K96 : 1 MACPLDQAIGLLVAIFHKYSGREGDKHTLSKKELKELIQKELTIGSKLQDAEIARLMEDL
 MA PLDQAIGLL+ IFHKYSG+EGDKHTLSKKELKELIQKELTIGSKLQDAEI +LM+DL
1A03 : 1 MASPLDQAIGLLIGIFHKYSGKEGDKHTLSKKELKELIQKELTIGSKLQDAEIVKLMDDL

1K96 : DRNKDQEVNFQEYVTFLGALALIYNEALKG 90
 DRNKDQEVNFQEY+TFLGALA+IYNEALKG
1A03 : DRNKDQEVNFQEYITFLGALAMIYNEALKG 90

**id = 3ko0_T I = 50% E = 1.2E-22**
1K96 : 1 MACPLDQAIGLLVAIFHKYSGREGDKHTLSKKELKELIQKELT--IGSKLQDAEIARLME
 MACPL++A+ ++V+ FHKYSG+EGDK L+K ELKEL+ +EL +G + +A +LM
3KO0 : 1 MACPLEKALDVMVSTFHKYSGKEGDKFKLNKSELKELLTRELPSFLGKRTDEAAFQKLMS

1K96 : DLDRNKDQEVNFQEYVTFLGALALIYNEALKG 90
 +LD N+D EV+FQEY FL +A++ NE +G
3KO0: NLDSNRDNEVDFQEYCVFLSCIAMMCNEFFEG 92

**id = 2rgi_B I = 46% E = 1.4E-18**
1K96 : 1 MACPLDQAIGLLVAIFHKYSGREGDKHTLSKKELKELIQKELT--IGSKLQDAEIARLME
 M+ L+QA+ +LV FHKYS +EGDK LSK E+KEL+ KEL +G K+ + + +LM
2RGI : 2 MSSSLEQALAVLVTTFHKYSSQEGDKFKLSKGEMKELLHKELPSFVGEKVDEEGLKKLMG

1K96 : DLDRNKDQEVNFQEYVTFLGALALIYNEALKG 90
 LD N DQ+V+FQEY FL + ++ N+ +G
2RGI : SLDENSDQQVDFQEYAVFLALITVMSNDFFQG 93

**id = 1qls_A I = 30% E = 5.5E-07**
1K96 : 6 DQAIGLLVAIFHKYSGREGDKHTLSKKELKELIQKELTIGSKLQ--DAEIARLMEDLDRN
 ++ I L+AIF K++GR+G+ +SK E + EL ++ Q + R+M+ LD +
1QLS : 9 ERCIESLIAIFQKHAGRDGNNTKISKTEFLIFMNTELAAFTQNQKDPGVLDRMMKKLDLD

1K96 : KDQEVNFQEYVTFLGALALIYNEAL 88
 D +++FQE++ +G LA+ +++
1QLS : SDGQLDFQEFLNLIGGLAIACHDSF 93

**>1GU2**

DVTNAEKLVYKYTNIAHSANPMYEAPSITDGKIFFNRKFKTPSGKEAACASCHTNNPANVGKNIVTGKEIPPLAPRVNTKRFTDIDKVEDEFTKHCNDILGADCSPSEKANFIAYLLTETKPTK

**Similar protein structures from the Protein Data Bank:** [**7**](http://www.sbkb.org/kb/report.jsp?sid=s1323076788-83&pid=structures)

**Proteins with highly similar structures in PDB**

(<http://zhanglab.ccmb.med.umich.edu/I-TASSER/output/S89012/>)

**id = 1dw3_C I = 41% E = 1.0E-12 TM_Score : 0.761 RMSD 1.96**
1GU2 : 48 ACASCHTNNPANVGKNIVTGKEIPPLAPRVNTKRFTDIDKVEDEFTKHCNDILGADCSPS
 +C +CH + G+ TGKEI PLAP RFTD +VE ++CN ++G DC+P
1DW3 : 42 SCTTCHGADVTRAGQT-RTGKEIAPLAPSATPDRFTDSARVEKWLGRNCNSVIGRDCTPG

1GU2 : EKANFIAYLLTE 119
 EKA+ +A+L +
1DW3 : EKADLLAWLAAQ 112

>**1NZN**

MEAVLNELVSVEDLLKFEKKFQSEKAAGSVSKSTQFEYAWCLVRTRYNDDIRKGIVLLEELLPKGSKEEQRDYVFYLAVGNYRLKEYEKALKYVRGLLQTEPQNNQAKELERLIDKAMKKDGLVGMAIVGGMALGVAGLAGLIGLAVSKSKS

**Similar protein structures from the Protein Data Bank:** [**7**](http://www.sbkb.org/kb/report.jsp?sid=s1323077845-87&pid=structures)

**Proteins with highly similar structures in PDB**

(<http://zhanglab.ccmb.med.umich.edu/I-TASSER/output/S85147/>)

5: 13 - EAVLNELVSVED

**id = 1iyg_A I = 91% E = 5.1E-62 TM_Score : 0.743 RMSD 1.43**
1NZN : 2 SHMEAVLNELVSVEDLLKFEKKFQSEKAAGSVSKSTQFEYAWCLVRTRYNDDIRKGIVLL
 S MEAVLNELVSVEDL FE+KFQSE+AAGSVSKSTQFEYAWCLVR++YN+DIR+GIVLL
1IYG : 6 SGMEAVLNELVSVEDLKNFERKFQSEQAAGSVSKSTQFEYAWCLVRSKYNEDIRRGIVLL

1NZN : EELLPKGSKEEQRDYVFYLAVGNYRLKEYEKALKYVRGLLQTEPQNNQAKELERLIDKAM
 EELLPKGSKEEQRDYVFYLAVGNYRLKEYEKALKYVRGLLQTEPQNNQAKELERLIDKAM
1IYG : EELLPKGSKEEQRDYVFYLAVGNYRLKEYEKALKYVRGLLQTEPQNNQAKELERLIDKAM

1NZN : KKDG 125
 KK G
1IYG : KKSG 129

**id = 3o48_A I = 28% E = 5.4E-08 TM_Score : 0.726 RMSD 2.08**
1NZN : 29 AAGSVSKSTQFEYAWCLVRTRYNDDIRKGIVLLEELLPKGSKEEQRDYVFYLAVGNYRLK
 + + ++F YAW L+++ +D R G+ +L ++ K ++ +R+ ++YL +G Y+L
3O48 : 34 GGPTATIQSRFNYAWGLIKSTDVNDERLGVKILTDIY-KEAESRRRECLYYLTIGCYKLG

1NZN : EYEKALKYVRGLLQTEPQNNQAKELERLIDKAMKKD 124
 EY A +YV L + E N Q L+ +++ ++K+
3O48 : EYSMAKRYVDTLFEHERNNKQVGALKSMVEDKIQKE 128

**id = 1y8m_A I = 28% E = 2.3E-08 TM_Score : 0.711 RMSD 2.12**
1NZN : 29 AAGSVSKSTQFEYAWCLVRTRYNDDIRKGIVLLEELLPKGSKEEQRDYVFYLAVGNYRLK
 + + ++F YAW L+++ +D R G+ +L ++ K ++ +R+ ++YL +G Y+L
1Y8M : 33 GGPTATIQSRFNYAWGLIKSTDVNDERLGVKILTDIY-KEAESRRRECLYYLTIGCYKLG

1NZN : EYEKALKYVRGLLQTEPQNNQAKELERLIDKAMKKDGL 126
 EY A +YV L + E N Q L+ +++ ++K+ L
1Y8M : EYSMAKRYVDTLFEHERNNKQVGALKSMVEDKIQKETL 129

**>1OMR** GNSKSGALSKEILEELQLNTKFTEEELSSWYQSFLKECPSGRITRQEFQTIYSKFFPEADPKAYAQHVFRSFDANSDGTLDFKEYVIALHMTSAGKTNQKLEWAFSLYDVDGNGTISKNEVLEIVTAIFKMISPEDTKHLPEDENTPEKRAEKIWGFFGKKDDDKLTEKEFIEGTLANKEILRLIQFEPQKVKEKLKEKKL

**Similar protein structures from the Protein Data Bank:** [**175**](http://www.sbkb.org/kb/report.jsp?sid=s1323077470-98&pid=structures)

**Proteins with highly similar structures in PDB**

(<http://zhanglab.ccmb.med.umich.edu/I-TASSER/output/S88727/>)

**id = 2d8n_A I = 88% E = 1.5E-98 TM_Score : 0.862 RMSD 1.95**
1OMR : 7 ALSKEILEELQLNTKFTEEELSSWYQSFLKECPSGRITRQEFQTIYSKFFPEADPKAYAQ
 ALSKEILEELQLNTKF+EEEL SWYQSFLK+CP+GRIT+Q+FQ+IY+KFFP+ DPKAYAQ
2D8N : 15 ALSKEILEELQLNTKFSEEELCSWYQSFLKDCPTGRITQQQFQSIYAKFFPDTDPKAYAQ

1OMR : HVFRSFDANSDGTLDFKEYVIALHMTSAGKTNQKLEWAFSLYDVDGNGTISKNEVLEIVT
 HVFRSFD+N DGTLDFKEYVIALHMT+AGKTNQKLEWAFSLYDVDGNGTISKNEVLEIV
2D8N : HVFRSFDSNLDGTLDFKEYVIALHMTTAGKTNQKLEWAFSLYDVDGNGTISKNEVLEIVM

1OMR : AIFKMISPEDTKHLPEDENTPEKRAEKIWGFFGKKDDDKLTEKEFIEGTLANKEILRLIQ
 AIFKMI+PED K LP+DENTPEKRAEKIW +FGK DDDKLTEKEFIEGTLANKEILRLIQ
2D8N : AIFKMITPEDVKLLPDDENTPEKRAEKIWKYFGKNDDDKLTEKEFIEGTLANKEILRLIQ

1OMR : FEPQKVKEKLK 197
 FEPQKVKEK+K
2D8N : FEPQKVKEKMK 205

**id = 1bjf_B I = 52% E = 5.0E-54 TM_Score : 0.780 RMSD 2.19**
1OMR : 2 NSKSGALSKEILEELQLNTKFTEEELSSWYQSFLKECPSGRITRQEFQTIYSKFFPEADP
 NSK L E++++L +T FTE E+ WY+ FL++CPSG ++ +EF+ IY FFP D
1BJF : 5 NSK---LRPEVMQDLLESTDFTEHEIQEWYKGFLRDCPSGHLSMEEFKKIYGNFFPYGDA

1OMR : KAYAQHVFRSFDANSDGTLDFKEYVIALHMTSAGKTNQKLEWAFSLYDVDGNGTISKNEV
 +A+HVFR+FDAN DGT+DF+E++IAL +TS GK QKL+WAFS+YD+DGNG ISK E+
1BJF : SKFAEHVFRTFDANGDGTIDFREFIIALSVTSRGKLEQKLKWAFSMYDLDGNGYISKAEM

1OMR : LEIVTAIFKMISPEDTKHLPEDENTPEKRAEKIWGFFGKKDDDKLTEKEFIEGTLANKEI
 LEIV AI+KM+S +PEDE+TPEKR EKI+ D KL+ +EFI G ++ I
1BJF : LEIVQAIYKMVS--SVMKMPEDESTPEKRTEKIFRQMDTNRDGKLSLEEFIRGAKSDPSI

1OMR : LRLIQFEP 189
 +RL+Q +P
1BJF : VRLLQCDP 187

**id = 2l2e_A I = 48% E = 3.2E-46**
1OMR : 1 GNSKSGALSKEILEELQLNTKFTEEELSSWYQSFLKECPSGRITRQEFQTIYSKFFPEAD
 G S+S LS++ L++L +T+F ++EL WY+ F K+CPSG + + EFQ IY +FFP D
2L2E : 2 GKSQS-KLSQDQLQDLVRSTRFDKKELQQWYKGFFKDCPSGHLNKSEFQKIYKQFFPFGD

1OMR : PKAYAQHVFRSFDANSDGTLDFKEYVIALHMTSAGKTNQKLEWAFSLYDVDGNGTISKNE
 P A+A++VF FDA+ +G +DFKE++ AL +TS G+ N KL WAF LYD+D NG IS +E
2L2E : PSAFAEYVFNVFDADKNGYIDFKEFICALSVTSRGELNDKLIWAFQLYDLDNNGLISYDE

1OMR : VLEIVTAIFKMISPEDTKHLPEDENTPEKRAEKIWGFFGKKDDDKLTEKEFIEGTLANKE
 +L IV AI+KM+ LPEDE+TPEKR KI+ K D +LT +EF EG+ +
2L2E : MLRIVDAIYKMVGS--MVKLPEDEDTPEKRVNKIFNMMDKNKDGQLTLEEFCEGSKRDPT

1OMR : IL 182
 I+
2L2E : IV 180

**id = 1g8i_B I = 47% E = 4.5E-45 TM_Score : 0.758 RMSD 2.68**
1OMR : 1 GNSKSGALSKEILEELQLNTKFTEEELSSWYQSFLKECPSGRITRQEFQTIYSKFFPEAD
 G S S L E++EEL T FTE+E+ WY+ F+K+CPSG++ FQ IY +FFP D
1G8I : 2 GKSNS-KLKPEVVEELTRKTYFTEKEVQQWYKGFIKDCPSGQLDAAGFQKIYKQFFPFGD

1OMR : PKAYAQHVFRSFDANSDGTLDFKEYVIALHMTSAGKTNQKLEWAFSLYDVDGNGTISKNE
 P +A VF FD N DG ++F E++ AL +TS G ++KL WAF LYD+D +G I++NE
1G8I : PTKFATFVFNVFDENKDGRIEFSEFIQALSVTSRGTLDEKLRWAFKLYDLDNDGYITRNE

1OMR : VLEIVTAIFKMISPEDTKHLPEDENTPEKRAEKIWGFFGKKDDDKLTEKEFIEGTLANKE
 +L+IV AI++M+ +T LPE+ENTPEKR ++I+ K D KLT +EF EG+ A+
1G8I : MLDIVDAIYQMVG--NTVELPEEENTPEKRVDRIFAMMDKNADGKLTLQEFQEGSKADPS

1OMR : ILRLI 185
 I++ +
1G8I : IVQAL 183

**id = 1s1e_A I = 36% E = 2.0E-30 TM_Score : 0.757 RMSD 2.53**
1OMR : 11 EILEELQLNTKFTEEELSSWYQSFLKECPSGRITRQEFQTIYSKFFPEADPKAYAQHVFR
 E LE+L+ T FT+ EL Y+ F ECPSG + + F+ IY++FFP D YA ++F
1SLE : 37 EGLEQLEAQTNFTKRELQVLYRGFKNECPSGVVNEETFKQIYAQFFPHGDASTYAHYLFN

1OMR : SFDANSDGTLDFKEYVIALHMTSAGKTNQKLEWAFSLYDVDGNGTISKNEVLEIVTAIFK
 +FD G++ F+++V AL + G ++KL W F+LYD++ +G I+K E+++IV AI+
1SLE : AFDTTQTGSVKFEDFVTALSILLRGTVHEKLRWTFNLYDINKDGYINKEEMMDIVKAIYD

1OMR : MISPEDTKHLPEDENTPEKRAEKIWGFFGKKDDDKLTEKEFIEGTLANKEILRLIQF 187
 M+ L ED TP + + + K D +T EF+E + I+R +Q
1SLE : MMGKYTYPVLKED--TPRQHVDVFFQKMDKNKDGIVTLDEFLESCQEDDNIMRSLQL 211

**id = 1fpw_A I = 39% E = 3.0E-35 TM_Score : 0.755 RMSD 2.75**
1OMR : 2 NSKSGALSKEILEELQLNTKFTEEELSSWYQSFLKECPSGRITRQEFQTIYSKFFPEADP
 +K+ LSK+ L L+ +T F E+ W++ FL++CPSG++ R++F IY +FFP P
1FPW : 2 GAKTSKLSKDDLTCLKQSTYFDRREIQQWHKGFLRDCPSGQLAREDFVKIYKQFFPFGSP

1OMR : KAYAQHVFRSFDANSDGTLDFKEYVIALHMTSAGKTNQKLEWAFSLYDVDGNGTISKNEV
 + +A H+F FD +++G + F+E++ L TS G +KL WAF LYD++ +G I+ +E+
1FPW : EDFANHLFTVFDKDNNGFIHFEEFITVLSTTSRGTLEEKLSWAFELYDLNHDGYITFDEM

1OMR : LEIVTAIFKMISPEDTKHLPEDENTPEKRAEKIWGFFGKKDDDKLTEKEFIEGTLANKEI
 L IV +++KM+ T L EDE TPE R +KI+ K +D +T EF EG+ + I
1FPW : LTIVASVYKMMGSMVT--LNEDEATPEMRVKKIFKLMDKNEDGYITLDEFREGSKVDPSI

1OMR : L 182
 +
1FPW : I 180

**id = 2jul_A I = 36% E = 3.0E-31 TM_Score : 0.704 RMSD 3.22**
1OMR : 11 EILEELQLNTKFTEEELSSWYQSFLKECPSGRITRQEFQTIYSKFFPEADPKAYAQHVFR
 E L++LQ TKFT++EL S Y+ F ECP+G + F+ IYS+FFP+ D YA +F
2JUL : 77 EGLDQLQAQTKFTKKELQSLYRGFKNECPTGLVDEDTFKLIYSQFFPQGDATTYAHFLFN

1OMR : SFDANSDGTLDFKEYVIALHMTSAGKTNQKLEWAFSLYDVDGNGTISKNEVLEIVTAIFK
 +FDA+ +G + F+++V+ L + G ++KL+WAF+LYD++ +G I+K E+L I+ +I+
2JUL : AFDADGNGAIHFEDFVVGLSILLRGTVHEKLKWAFNLYDINKDGCITKEEMLAIMKSIYD

1OMR : MISPEDTKHLPEDENTPEKRAEKIWGFFGKKDDDKLTEKEFIEGTLANKEILRLIQF 187
 M+ L ED P + E+ + + D +T EF+E ++ I+ +Q
2JUL : MMGRHTYPILRED--APLEHVERFFQKMDRNQDGVVTIDEFLETCQKDENIMNSMQL 251

**id = 1jba_A I = 36% E = 2.0E-26 TM_Score : 0.702 RMSD 3.41**
1OMR : 26 ELSSWYQSFLKECPSGRITRQEFQTIYSKFFPEADPKAYAQHV---FRSFDANSDGTLDF
 +L WY+ FL+ECPSG + EF+ +FF D + Q+V FR+FD N D T+DF
1JBA : 23 QLQEWYKKFLEECPSGTLFMHEFK----RFFKVPDNEEATQYVEAMFRAFDTNGDNTIDF

1OMR : KEYVIALHMTSAGKTNQKLEWAFSLYDVDGNGTISKNEVLEIVTAIFKMISPEDTKHLPE
 EYV AL++ G KL+W F +YD D NG I + E+L+IV +I+K+ + E
1JBA : LEYVAALNLVLRGTLEHKLKWTFKIYDKDRNGCIDRQELLDIVESIYKLKKACSVEVEAE

1OMR : DEN---TPEKRAEKIWGFFGKKDDDKLTEKEFIEGTLANKEILRLIQFE 188
 + TPE+ ++I+ + D +L+ EF+EG +K +++++Q +
1JBA : QQGKLLTPEEVVDRIFLLVDENGDGQLSLNEFVEGARRDKWVMKMLQMD 187

**id = 1v1g_A I = 31% E = 6.9E-08 TM_Score : 0.672 RMSD 3.48**
1OMR : 20 TKFTEEELSSWYQSFLKECPS----GRITRQEFQTIYSKFFPEADPKAYAQHVFRSFDAN
 T FT EE+ + Y+ F K S G I ++EFQ + F +A +F FD
1V1G : 29 TPFTVEEVEALYELFKKLSSSIIDDGLIHKEEFQ--LALFRNRNRRNLFADRIFDVFDVK

1OMR : SDGTLDFKEYVIALHM-TSAGKTNQKLEWAFSLYDVDGNGTISKNEVLEIVTAIF 129
 +G ++F E+V +L + + ++K+++AF LYD+ G I + E+ E+V A+
1V1G : RNGVIEFGEFVRSLGVFHPSAPVHEKVKFAFKLYDLRQTGFIEREELKEMVVALL 141

**id = 1uhn_A I = 30% E = 3.8E-06 TM_Score : 0.634 RMSD 3.42**
1OMR : 14 EELQLNTKFTEEELSSWYQSFLKECPS----GRITRQEFQTIYSKFFPEADPKAYAQHVF
 E L +T F+ E+ + Y+ F K + G I ++EFQ + F +A VF
1UHN : 3 ELLARDTVFSVSEIEALYELFKKISSAVIDDGLINKEEFQ--LALFKTNKKESLFADRVF

1OMR : RSFDANSDGTLDFKEYVIALHMTSA-GKTNQKLEWAFSLYDVDGNGTISKNEVLEIVTA 127
 FD +G L F+E+ AL + + K+ ++F LYD+ G I + EV ++V A
1UHN : DLFDTKHNGILGFEEFARALSVFHPNAPIDDKIHFSFQLYDLKQQGFIERQEVKQMVVA 119

**>1I27**

GPLGSGDVQVTEDAVRRYLTRKPMTTKDLLKKFQTKKTGLSSEQTVNVLAQILKRLNPERKMINDKMHFSLKE

**Similar protein structures from the Protein Data Bank:** [**5**](http://www.sbkb.org/kb/report.jsp?sid=s1323078024-2&pid=structures)

**Proteins with highly similar structures in PDB**

(<http://zhanglab.ccmb.med.umich.edu/I-TASSER/output/S89013>)

TASEER has found similar domain in the larger structures.

2ZJ8, 2P6R, 2P6U, 2VA8, 2AS5, 3G73, 2DQL, 2ZFW

**>1HH5**

ADVVTYENKKGNVTFDHKAHAEKLGCDACHEGTPAKIAIDKKSAHKDACKTCHKSNNGPTKCGGCHIK

**Similar protein structures from the Protein Data Bank:** [13](http://www.sbkb.org/kb/report.jsp?sid=s1323078082-8&pid=structures)

**Proteins with highly similar structures in PDB**

(<http://zhanglab.ccmb.med.umich.edu/I-TASSER/output/S88803/>)

**id = 3h33_A I = 45% E = 1.5E-08 TM_Score : 0.838 RMSD 1.54**
1HH5 : 2 DVVTYENKKGNVTFDHKAHAEKLG-CDACHEGTPAKI-AIDKKSAHKDACKTCHKSNN-G
 D +TY + G V F HK H + LG C CHE P +I DK AH CK CH+ G
3H33 : 2 DKITYPTRIGAVVFPHKKHQDALGECRGCHEKGPGRIDGFDKVMAHGKGCKGCHEEMKIG

1HH5 : PTKCGGCH 66
 P +CG CH
3H33 : PVRCGDCH 69

**id = 3bxu_B I = 50% E = 2.3E-09 TM_Score : 0.827 RMSD 1.75**
1HH5 : 1 ADVVTYENKKGNVTFDHKAHAEKL-GCDACHEGTPAKI-AIDKKSAHKDACKTCHKS-NN
 AD +T+ K GNVTFDHK H + C CH TP KI K+ AH +CK CH+
3BXU : 1 ADTMTFTAKNGNVTFDHKKHQTIVPDCAVCHGKTPGKIEGFGKEMAHGKSCKGCHEEMKK

1HH5 : GPTKCGGCHIK 68
 GPTKCG CH K
3BXU : GPTKCGECHKK 71

**id = 3h34_A I = 50% E = 3.6E-08 TM_Score : 0.790 RMSD 1.66**
1HH5 : 1 ADVVTYENKKGNVTFDHKAHAEKLG-CDACHEGTPAKIA-IDKKSAHKDACKTCHKSNN-
 ADV+ + +K G VTF HK H+E + C +CHE TP KI K AHK CK CH+
3H34 : 1 ADVILFPSKNGAVTFTHKRHSEFVRECRSCHEKTPGKIRNFGKDYAHK-TCKGCHEVRGA

1HH5 : GPTKCGGCH 66
 GPTKC CH
3H34 : GPTKCKLCH 68

**id = 2ldo_A I = 46% E = 2.5E-07 TM_Score : 0.779 RMSD 1.82**
1HH5 : 1 ADVVTYENKKGNVTFDHKAHAEKL-GCDACHEGTPAKI-AIDKKSAHKDACKTCHKS-NN
 AD + + K G+V F HKAH + + C CHE P KI K+ AH CK CH+
2LDO : 1 ADDIVLKAKNGDVKFPHKAHQKAVPDCKKCHEKGPGKIEGFGKEMAHGKGCKGCHEEMKK

1HH5 : GPTKCGGCHIK 68
 GPTKCG CH K
2LDO : GPTKCGECHKK 71

**id = 1os6_A I = 46% E = 2.5E-07**
1HH5 : 1 ADVVTYENKKGNVTFDHKAHAEKL-GCDACHEGTPAKI-AIDKKSAHKDACKTCHKS-NN
 AD + + K G+V F HKAH + + C CHE P KI K+ AH CK CH+
1OS6 : 1 ADDIVLKAKNGDVKFPHKAHQKAVPDCKKCHEKGPGKIEGFGKEMAHGKGCKGCHEEMKK

1HH5 : GPTKCGGCHIK 68
 GPTKCG CH K
1OS6 : GPTKCGECHKK 71

**id = 3h4n_B I = 57% E = 2.8E-07 TM_Score : 0.720 RMSD 1.79**
1HH5 : 3 VVTYENKKGNVTFDHKAHAEKLG-CDACHEGTP-AKIA-IDKKSAHKDACKTCHKS-NNG
 VV E K GNVTFDHK HA G C ACHE KIA + K AHK C CHK G
3H4N : 4 VVVLEAKNGNVTFDHKKHAGVKGECKACHETEAGGKIAGMGKDWAHK-TCTGCHKEMGKG

1HH5 : PTKCGGCHIK 68
 PTKCG CH K
3H4N : PTKCGECHKK 72

**>1H0A**

MSTSSLRRQMKNIVHNYSEAEIKVREATSNDPWGPSSSLMSEIADLTYNVVAFSEIMSMIWKRLNDHGKNWRHVYKAMTLMEYLIKTGSERVSQQCKENMYAVQTLKDFQYVDRDGKDQGVNVREKAKQLVALLRDEDRLREERAHALKTKEKLAQTA

**Similar protein structures from the Protein Data Bank:** [**9**](http://www.sbkb.org/kb/report.jsp?sid=s1323078163-96&pid=structures)

**Proteins with highly similar structures in PDB**

(<http://zhanglab.ccmb.med.umich.edu/I-TASSER/output/S89014/>)

**id = 3onl_B I = 46% E = 2.0E-33 TM_Score : 0.833 RMSD 1.02**
1H0A : 16 NYSEAEIKVREATSNDPWGPSSSLMSEIADLTYNVVAFSEIMSMIWKRLNDH-GKNWRHV
 NY+E E KVREAT+N+PWG SS+LM +I+ TYN EI+SMI++R + G WR +
3ONL : 8 NYTEMEGKVREATNNEPWGASSTLMDQISQGTYNFREREEILSMIFRRFTEKAGSEWRQI

1H0A : YKAMTLMEYLIKTGSERVSQQCKENMYAVQTLKDFQYVDRDGKDQGVNVREKAKQLVALL
 YKA+ L++YLIK GSER + ++ ++ L+ F Y+D G+DQG+NVR + K L+ LL
3ONL : YKALQLLDYLIKHGSERFIDDTRNSINLIRILETFHYIDSQGRDQGINVRTRVKALIELL

1H0A : RDEDRLREERAHALKTKEK 153
 D++++R ER A +T +K
3ONL : SDDNKIRAERKKARETAKK 146

**id = 2qy7_C I = 54% E = 1.2E-38 TM_Score : 0.821 RMSD 1.02**
1H0A : 16 NYSEAEIKVREATSNDPWGPSSSLMSEIADLTYNVVAFSEIMSMIWKR-LNDHGKNWRHV
 NYSE E KVREAT++DPWGPS LM EIA T+ F E+M+M+W R L D+ KNWR V
2QY7 : 1 NYSEIESKVREATNDDPWGPSGQLMGEIAKATFMYEQFPELMNMLWSRMLKDNKKNWRRV

1H0A : YKAMTLMEYLIKTGSERVSQQCKENMYAVQTLKDFQYVDRDGKDQGVNVREKAKQLVALL
 YK++ L+ YLI+ GSERV +E++Y +++L+++ +VD GKDQG+N+R+K K+LV
2QY7 : YKSLLLLAYLIRNGSERVVTSAREHIYDLRSLENYHFVDEHGKDQGINIRQKVKELVEFA

1H0A : RDEDRLREER 144
 +D+DRLREER
2QY7 : QDDDRLREER 130

**id = 1xgw_A I = 54% E = 6.1E-40**
1H0A : 12 NIVHNYSEAEIKVREATSNDPWGPSSSLMSEIADLTYNVVAFSEIMSMIWKR-LNDHGKN
 N+V NYSE E KVREAT++DPWGPS LM EIA T+ F E+M+M+W R L D+ KN
1XGW : 27 NVVMNYSEIESKVREATNDDPWGPSGQLMGEIAKATFMYEQFPELMNMLWSRMLKDNKKN

1H0A : WRHVYKAMTLMEYLIKTGSERVSQQCKENMYAVQTLKDFQYVDRDGKDQGVNVREKAKQL
 WR VYK++ L+ YLI+ GSERV +E++Y +++L+++ +VD GKDQG+N+R+K K+L
1XGW : WRRVYKSLLLLAYLIRNGSERVVTSAREHIYDLRSLENYHFVDEHGKDQGINIRQKVKEL

1H0A : VALLRDEDRLREER 144
 V +D+DRLREER
1XGW : VEFAQDDDRLREER 160

**>5P2P**

ALFQFRSMIKCAIPGSHPLMDFNNYGCYCGWGGSGTPVDELDRCCETHDNCYRDAKNLSGCYPYTESYSYSCSNTEITCNSKNNACEAFICNCDRNAAICFSKAPYNKEHKNLDTKKYC

**Similar protein structures from the Protein Data Bank:** [**257**](http://www.sbkb.org/kb/report.jsp?sid=s1323078405-93&pid=structures)

**Proteins with highly similar structures in PDB**

(<http://zhanglab.ccmb.med.umich.edu/I-TASSER/output/S85966/>)

**id = 1m8t_F I = 61% E = 9.7E-32 TM_Score : 0.982 RMSD 0.55**
5P2P : 2 LFQFRSMIKCAIPGSHPLMDFNNYGCYCGWGGSGTPVDELDRCCETHDNCYRDAKNLSGC
 L QF MI+C IPGS P D+++YGCYCG GGSGTPVDELDRCC+ HDNCY A+ L+ C
1M8T : 2 LVQFNGMIRCTIPGSIPWWDYSDYGCYCGSGGSGTPVDELDRCCQVHDNCYTQAQQLTEC

5P2P : YPYTESYSYSCSNTEITCNSKNNACEAFICNCDRNAAICFSKAPYNKEHKNLDTKKYC 119
 PY++ YSY CS +TC + N+ C AF+C+CDR AAICF+ APYNKE+ N+DT C
1M8T : SPYSKRYSYDCSEGTLTCKADNDECAAFVCDCDRVAAICFAGAPYNKENINIDTTTRC 119

**id = 1y6o_B I = 89% E = 1.5E-58 TM_Score : 0.975 RMSD 0. 71**
5P2P : 1 ALFQFRSMIKCAIPGSHPLMDFNNYGCYCGWGGSGTPVDELDRCCETHDNCYRDAKNLSG
 AL+QFRS+IKCAIPGSHPL+DFNNYGCYCG GGSGTPVDELDRCCETHD CYRDAKNL
1Y6O : 1 ALWQFRSLIKCAIPGSHPLLDFNNYGCYCGLGGSGTPVDELDRCCETHDCCYRDAKNLDS

5P2P : CY-----PYTESYSYSCSNTEITCNSKNNACEAFICNCDRNAAICFSKAPYNKEHKNLDT
 C PYTESYSYSCSNTEITCNSKNNACEAFICNCDRNAAICFSKAPYNKEHKNLDT
1Y6O : CKFLVDNPYTESYSYSCSNTEITCNSKNNACEAFICNCDRNAAICFSKAPYNKEHKNLDT

5P2P : KKYC 119
 KKYC
1Y6O : KKYC 124

**id = 1s6b_B I = 55% E = 4.5E-34 TM_Score : 0.968 RMSD 0.65**
5P2P : 3 FQFRSMIKCAIPGSHPLMDFNNYGCYCGWGGSGTPVDELDRCCETHDNCYRDAKNLSGCY
 +QF++MI C +P S DF +YGCYCG GGSGTPVD+LDRCC+ HDNCY +A+ +SGC
1S6B : 3 WQFKNMISCTVP-SRSWWDFADYGCYCGRGGSGTPVDDLDRCCQVHDNCYNEAEKISGCN

5P2P : PYTESYSYSCSNTEITCNSKNNACEAFICNCDRNAAICFSKAPYNKEHKNLDTKKYC 119
 P +YSY C+ +TC +NNAC A +C+CDR AAICF+ APYN + N+D + C
1S6B : PRFRTYSYECTAGTLTCTGRNNACAASVCDCDRLAAICFAGAPYNDNNYNIDLQARC 118

**id = 1bpq_A I = 76% E = 1.3E-48 TM_Score : 0.967 RMSD 0.74**
5P2P : 1 ALFQFRSMIKCAIPGSHPLMDFNNYGCYCGWGGSGTPVDELDRCCETHDNCYRDAKNLSG
 AL+QF MIKC IP S PL+DFNNYGCYCG GGSGTPVD+LDRCC+THDNCY+ A L
1BPQ : 1 ALWQFNGMIKCKIPSSEPLLDFNNYGCYCGLGGSGTPVDDLDRCCQTHDNCYKQAMKLDS

5P2P : CY-----PYTESYSYSCSNTEITCNSKNNACEAFICNCDRNAAICFSKAPYNKEHKNLDT
 C PYT +YSYSCSN EITC+S+NNACEAFICNCDRNAAICFSK PYNKEHKNLD
1BPQ : CKVLVDNPYTNNYSYSCSNNEITCSSENNACEAFICNCDRNAAICFSKVPYNKEHKNLDK

5P2P : K 116
 K
1BPQ : K 121

**id = 1mh2_B I = 56% E = 5.3E-35 TM_Score : 0.966 RMSD 0.68**
5P2P : 3 FQFRSMIKCAIPGSHPLMDFNNYGCYCGWGGSGTPVDELDRCCETHDNCYRDAKNLSGCY
 +QF++MI C +P S DF +YGCYCG GGSGTP D+LDRCC+THDNCY +A+ +SGC
1MH2 : 3 WQFKNMISCTVP-SRSWWDFADYGCYCGRGGSGTPSDDLDRCCQTHDNCYNEAEKISGCN

5P2P : PYTESYSYSCSNTEITCNSKNNACEAFICNCDRNAAICFSKAPYNKEHKNLDTKKYC 119
 P +YSY+C+ +TC +NNAC A +C+CDRNAAICF+ APYN + N+D + C
1MH2 : PRFRTYSYACTAGTLTCTGRNNACAASVCDCDRNAAICFAGAPYNDSNYNIDLQARC 118

**id = 1pob_B I = 57% E = 4.2E-36 TM_Score : 0.963 RMSD 0.73**
5P2P : 2 LFQFRSMIKCAIPGSHPLMDFNNYGCYCGWGGSGTPVDELDRCCETHDNCYRDAKNLSGC
 L+QF++MI+C +P S DF +YGCYCG GGSGTPVD+LDRCC+ HDNCY +A+ +SGC
1POB : 2 LYQFKNMIQCTVP-SRSWWDFADYGCYCGRGGSGTPVDDLDRCCQVHDNCYNEAEKISGC

5P2P : YPYTESYSYSCSNTEITCNSKNNACEAFICNCDRNAAICFSKAPYNKEHKNLDTKKYC 119
 +PY ++YSY CS +TC NNAC A +C+CDR AAICF+ APYN N++ K C
1POB : WPYFKTYSYECSQGTLTCKGGNNACAAAVCDCDRLAAICFAGAPYNDNDYNINLKARC 118

**id = 1mh7_A I = 52% E = 1.4E-25 TM_Score : 0.962 RMSD 0.73**

5P2P : 2 LFQFRSMIKCAIPGSHPLMDFNNYGCYCGWGGSGTPVDELDRCCETHDNCYRDAKNLSGC
 L+QF++MI+C +P + DF +YGCYCG GGSGTP D+LDRCC+ HDNCY A+ ++GC
1MH7 : 2 LYQFKNMIECTVP-ARSWWDFADYGCYCGGGGSGTPTDDLDRCCQVHDNCYNQAQEITGC

5P2P : YPYTESYSYSCSNTEITCNSKNNACEAFICNCDRNAAICFSKAPYNKEHKNLDTKKYC 119
 P ++Y+Y C+ +TC +NN+C A C+CDR AAICF+ APYN + N+D K C
1MH7 : RPKWKTYTYQCTQGTLTCKGRNNSCAATTCDCDRLAAICFAGAPYNDTNYNIDLKARC 118

**id = 3jq5_A I = 59% E = 3.7E-37 TM_Score : 0.961 RMSD 0.76**
5P2P : 2 LFQFRSMIKCAIPGSHPLMDFNNYGCYCGWGGSGTPVDELDRCCETHDNCYRDAKNLSGC
 L+QF++MI+C +P S DF +YGCYCG GGSGTPVD+LDRCC+THDNCY +A+N+SGC
3JQ5 : 2 LYQFKNMIQCTVP-SRSWADFADYGCYCGKGGSGTPVDDLDRCCQTHDNCYNEAENISGC

5P2P : YPYTESYSYSCSNTEITCNSKNNACEAFICNCDRNAAICFSKAPYNKEHKNLDTKKYC 119
 PY ++YSY C+ +TC NNAC A +C+CDR AAICF+ APYN + N+D K C
3JQ5 : RPYFKTYSYECTQGTLTCKGDNNACAASVCDCDRLAAICFAGAPYNDANYNIDLKARC 118

**id = 1gp7_C I = 52% E = 2.8E-33 TM_Score : 0.958 RMSD 0.86**
5P2P : 2 LFQFRSMIKCAIPGSHPLMDFNNYGCYCGWGGSGTPVDELDRCCETHDNCYRDAKNLSGC
 L QF +MI+C +PG + + +YGCYCG GGSGTPVD+LDRCC+ HDNCY A+ L C
1GP7 : 29 LIQFGNMIQCTVPGFLSWIKYADYGCYCGAGGSGTPVDKLDRCCQVHDNCYTQAQKLPAC

5P2P : -----YPYTESYSYSCSNTEITCNSKNNACEAFICNCDRNAAICFSKAPYNKEHKNLDTK
 PY + YSY CS +TC + N+ C AFICNCDR AA CF+ +PYN + N+DT
1GP7 : SSIMDSPYVKIYSYDCSERTVTCKADNDECAAFICNCDRVAAHCFAASPYNNNNYNIDTT

5P2P : KYC 119
 C
1GP7 : TRC 151

**id = 1g4i_A I = 76% E = 3.3E-49**
5P2P : 1 ALFQFRSMIKCAIPGSHPLMDFNNYGCYCGWGGSGTPVDELDRCCETHDNCYRDAKNLSG
 AL+QF MIKC IP S PL+DFNNYGCYCG GGSGTPVD+LDRCC+THDNCY+ AK L
1G4I : 1 ALWQFNGMIKCKIPSSEPLLDFNNYGCYCGLGGSGTPVDDLDRCCQTHDNCYKQAKKLDS

5P2P : CY-----PYTESYSYSCSNTEITCNSKNNACEAFICNCDRNAAICFSKAPYNKEHKNLDT
 C PYT +YSYSCSN EITC+S+NNACEAFICNCDRNAAICFSK PYNKEHKNLD
1G4I : CKVLVDNPYTNNYSYSCSNNEITCSSENNACEAFICNCDRNAAICFSKVPYNKEHKNLDK

5P2P : K 116
 K
1G4I : K 121

**id = 1bun_A I = 46% E = 1.4E-27**
5P2P : 2 LFQFRSMIKCAIPGSHPLMDFNNYGCYCGWGGSGTPVDELDRCCETHDNCYRDAKNLSGC
 L F MI+ IP ++ +YGCYCG GGSG P+D LDRCC HDNCY DA+ C
1BUN : 2 LINFMEMIRYTIPCEKTWGEYADYGCYCGAGGSGRPIDALDRCCYVHDNCYGDAEKKHKC

5P2P : YPYTESYSYSCSNTEITCNSKNNACEAFICNCDRNAAICFSKAPYNKEHKNLDTKKYC 119
 P T+SYSY + I C C +C+CDR AA+CF + Y + HKN+DT ++C
1BUN : NPKTQSYSYKLTKRTIICYGAAGTCARIVCDCDRTAALCFGNSEYIEGHKNIDTARFC 119

**β-class of proteins**

| ***X-ray PDB*** | ***NMR PDB*** | ***Region of Structural Variation*** | *Link to TASSER results* |
| --- | --- | --- | --- |
| **1OPA** | 1B4M | 27: 35 - FATRKIAVR  5: 14 - NGTWEMESNE  72: 75 - EHTK | <http://zhanglab.ccmb.med.umich.edu/I-TASSER/output/S88306/> |
| **1XCA** | 1BLR | 28: 38 - LRKIAVAAASK  127: 137- DVVCTRVYVRE  60: 66 - TTEINFK | <http://zhanglab.ccmb.med.umich.edu/I-TASSER/output/S88410/> |
| **2GIM** | 1FA4 | 2: 7 - ETYTVKL  52: 60 - SADLAKSLS  83: 89 - GEYTFYC  90: 96 - EPHRGAG | <http://zhanglab.ccmb.med.umich.edu/I-TASSER/output/S88446/> |
| **1SPD** | 1RK7 | 41: 48 - GLHGFHVH  85: 89 - NVIA  97: 99 - VSI  116: 120- TLVVH  54: 60 - TAGCTSA  132: 137- EESTKT | <http://zhanglab.ccmb.med.umich.edu/I-TASSER/output/S88448/> |
| **1J2A** | 1CLH | 38: 45 – SGFYNNTT  48: 57 - RVIPGFMIQG  77: 80 - ADNG | <http://zhanglab.ccmb.med.umich.edu/I-TASSER/output/S88509/> |
| **1IAZ** | 1KD6 | 8: 15 - VIDGSALS  129: 138- DQRMYEELYY | <http://zhanglab.ccmb.med.umich.edu/I-TASSER/output/S88527/> |
| **1WHO** | 1BMW | 4: 8 - VTFTV  16: 23 - HLAVLVKY  28: 34 - MAEVELR  51: 55 - VWTFD  64: 70 - FNFRFLT  75: 82 - KNVFDDVV | <http://zhanglab.ccmb.med.umich.edu/I-TASSER/output/S88528/> |

**>1OPA**

MTKDQNGTWEMESNENFEGYMKALDIDFATRKIAVRLTQTKIIVQDGDNFKTKTNSTFRNYDLDFTVGVEFDEHTKGLDGRNVKTLVTWEGNTLVCVQKGEKENRGWKQWVEGDKLYLELTCGDQVCRQVFKKK

**Similar protein structures from the Protein Data Bank:** [**144**](http://www.sbkb.org/kb/report.jsp?sid=s1322748117-26&pid=structures)

**Proteins with highly similar structures in PDB**

(<http://zhanglab.ccmb.med.umich.edu/I-TASSER/output/S88306/>)

**ID = 1CRB I = 57% E = 1.6E-37 TM_Score : 0.976 RMSD 0.57**1OPA : 4 DQNGTWEMESNENFEGYMKALDIDFATRKIAVRLTQTKIIVQDGDNFKTKTNSTFRNYDL
 D NG W+M SNENFE Y++ALD++ A RKIA L K IVQDGD+ +T STFRNY +
1CRB : 3 DFNGYWKMLSNENFEEYLRALDVNVALRKIANLLKPDKEIVQDGDHMIIRTLSTFRNYIM

1OPA : DFTVGVEFDEHTKGLDGRNVKTLVTWEGNTLVCVQKGEKENRGWKQWVEGDKLYLELTCG
 DF VG EF+E G+D R T V+W+G+ L CVQKGEKE RGW QW+EGD+L+LE+
1CRB : DFQVGKEFEEDLTGIDDRKCMTTVSWDGDKLQCVQKGEKEGRGWTQWIEGDELHLEMRAE

1OPA : DQVCRQVFKK 133

C+QVFKK

1CRB : GVTCKQVFKK 132

**id = 1LPJ I = 57% E = 2.1E-40 TM_Score : 0.973 RMSD 0.62**
1OPA : 4 DQNGTWEMESNENFEGYMKALDIDFATRKIAVRLTQTKIIVQDGDNFKTKTNSTFRNYDL
 D +GTW + S++NFEGYM AL IDFATRKIA L K+I Q+GD+F TNS+ RNY +
1LPJ : 3 DLSGTWTLLSSDNFEGYMLALGIDFATRKIAKLLKPQKVIEQNGDSFTIHTNSSLRNYFV

1OPA : DFTVGVEFDEHTKGLDGRNVKTLVTWEGNTLVCVQKGEKENRGWKQWVEGDKLYLELTCG
 F VG EFDE +GLD R K+LV W+ + L C+QKGEK+NRGW W+EGDKL+LE+ C
1LPJ : KFKVGEEFDEDNRGLDNRKCKSLVIWDNDRLTCIQKGEKKNRGWTHWIEGDKLHLEMFCE

1OPA : DQVCRQVFKK 133
 QVC+Q F++
1LPJ : GQVCKQTFQR 132

**id = 1kqw_A I = 74% E = 7.5E-53 TM_Score : 0.973 RMSD 0.64**
1OPA : 4 DQNGTWEMESNENFEGYMKALDIDFATRKIAVRLTQTKIIVQDGDNFKTKTNSTFRNYDL
 D NGTWEM SN+NFE MKALDIDFATRKIAV L QTK+IVQ+GD F+TKT STFRNY++
1KQW : 3 DFNGTWEMLSNDNFEDVMKALDIDFATRKIAVHLKQTKVIVQNGDKFETKTLSTFRNYEV

1OPA : DFTVGVEFDEHTKGLDGRNVKTLVTWEGNTLVCVQKGEKENRGWKQWVEGDKLYLELTCG
 +F +G EFDE TKGLD R VKTLV W+G+ LVCVQKGEKENRGWKQW+EGD L+LE+ C
1KQW : NFVIGEEFDEQTKGLDNRTVKTLVKWDGDKLVCVQKGEKENRGWKQWIEGDLLHLEIHCQ

1OPA : DQVCRQVFKKK 134
 D+VC QVFKKK
1KQW : DKVCHQVFKKK 133

**id = 2rct_A I = 90% E = 6.1E-67 TM_Score : 0.972 RMSD 1.00**
1OPA : 2 TKDQNGTWEMESNENFEGYMKALDIDFATRKIAVRLTQTKIIVQDGDNFKTKTNSTFRNY
 T+DQNGTWEMESNENFEGYMKALDIDFATRKIAVRLTQTK+I QDGDNFKTKT STFRNY
2RCT : 5 TRDQNGTWEMESNENFEGYMKALDIDFATRKIAVRLTQTKVIDQDGDNFKTKTTSTFRNY

1OPA : DLDFTVGVEFDEHTKGLDGRNVKTLVTWEGNTLVCVQKGEKENRGWKQWVEGDKLYLELT
 D+DFTVGVEFDE+TK LD R+VK LVTWEG+ LVCVQKGEKENRGWKQW+EGDKLYLELT
2RCT : DVDFTVGVEFDEYTKSLDNRHVKALVTWEGDVLVCVQKGEKENRGWKQWIEGDKLYLELT

1OPA : CGDQVCRQVFKKK 134
 CGDQVCRQVFKKK
2RCT : CGDQVCRQVFKKK 137

**id = 1ggl_B I = 52% E = 1.5E-34 TM_Score : 0.956 RMSD 0.94**
1OPA : 7 GTWEMESNENFEGYMKALDIDFATRKIAVRLTQTKIIVQDGDNFKTKTNSTFRNYDLDFT
 G + S +N E Y++AL+I A RKIA+ L K I G++ +T STFRNY + F
1GGL : 6 GYYRFVSQKNMEDYLQALNISLAVRKIALLLKPDKEIEHQGNHMTVRTLSTFRNYTVQFD

1OPA : VGVEFDEHTKGLDGRNVKTLVTWEGNTLVCVQKGEKENRGWKQWVEGDKLYLELTCGDQV
 VGVEF+E + +DGR +T+VTWE LVCVQKGE NRGW+ W+EG+ LYLELT D V
1GGL : VGVEFEEDLRSVDGRKCQTIVTWEEEHLVCVQKGEVPNRGWRHWLEGEMLYLELTARDAV

1OPA : CRQVFKK 133
 C QVF+K
1GGL : CEQVFRK 132

**id = 1hmt_A I = 42% E = 8.5E-21 TM_Score : 0.915 RMSD 1.3**
1OPA : 7 GTWEMESNENFEGYMKALDIDFATRKIAVRLTQTKIIVQDGDNFKTKTNSTFRNYDLDFT
 GTW++ ++NF+ YMK+L + FATR++A T II ++GD KT+STF+N ++ F
1HMT : 6 GTWKLVDSKNFDDYMKSLGVGFATRQVASMTKPTTIIEKNGDILTLKTHSTFKNTEISFK

1OPA : VGVEFDEHTKGLDGRNVKTLVTWEGNTLVCVQKGE-KENRGWKQWVEGDKLYLELTCGDQ
 +GVEFDE T D R VK++VT +G LV +QK + +E ++ ++G KL L LT G
1HMT : LGVEFDETTA--DDRKVKSIVTLDGGKLVHLQKWDGQETTLVRELIDG-KLILTLTHGTA

1OPA : VCRQVFKKK 134
 VC + ++K+
1HMT : VCTRTYEKE 131

**id = 2wut_A I = 36% E = 1.7E-15 TM_Score : 0.913 RMSD 1.39**
1OPA : 7 GTWEMESNENFEGYMKALDIDFATRKIAVRLTQTKIIVQDGDNFKTKTNSTFRNYDLDFT
 GTW++ S+ENF+ YMKAL + ATRK+ T II + GD +T STF+N ++ F
2WUT : 8 GTWKLVSSENFDDYMKALGVGLATRKLGNLAKPTVIISKKGDIITIRTESTFKNTEISFK

1OPA : VGVEFDEHTKGLDGRNVKTLVTWEGNTLVCVQKGEKENRGWKQWVEGDKLYLELTCGDQV
 +G EF+E T D R K++VT + +L VQ+ + + K+ + K+ E V
2WUT : LGQEFEETTA--DNRKTKSIVTLQRGSLNQVQRWDGKETTIKRKLVNGKMVAECKMKGVV

1OPA : CRQVFKK 133
 C ++++K
2WUT : CTRIYEK 132

**id = 1b56_A I = 28% E = 8.9E-09 TM_Score : 0.911 RMSD 1.28**
1OPA : 7 GTWEMESNENFEGYMKALDIDFATRKIAVRLTQTKIIVQDGDNFKTKTNSTFRNYDLDFT
 G W + ++ F+ YMK L + A RK+ II DG N KT ST + T
1B56 : 9 GRWRLVDSKGFDEYMKELGVGIALRKMGAMAKPDCIITCDGKNLTIKTESTLKTTQFSCT

1OPA : VGVEFDEHTKGLDGRNVKTLVTWEGNTLVCVQKGEKENRGWKQWVEGDKLYLELTCGDQV
 +G +F+E T DGR +T+ + LV Q+ + + + ++ KL +E +
1B56 : LGEKFEETTA--DGRKTQTVCNFTDGALVQHQEWDGKESTITRKLKDGKLVVECVMNNVT

1OPA : CRQVFKK 133
 C ++++K
1B56 : CTRIYEK 133

**id = 4a60_A I = 34% E = 5.3E-15 TM_Score : 0.908 RMSD 1.37**
1OPA : 7 GTWEMESNENFEGYMKALDIDFATRKIAVRLTQTKIIVQDGDNFKTKTNSTFRNYDLDFT
 GTW++ S+ENFE YMK L ++FA R +A + T I DG +T S+F++ + F
4A60 : 29 GTWKLVSSENFEDYMKELGVNFAARNMAGLVKPTVTISVDGKMMTIRTESSFQDTKISFK

1OPA : VGVEFDEHTKGLDGRNVKTLVTWEGNTLVCVQKGEKENRGWKQWVEGDKLYLELTCGDQV
 +G EFDE T D R VK+ +T E +++ VQK + K+ + +K+ +E + V
4A60 : LGEEFDETTA--DNRKVKSTITLENGSMIHVQKWLGKETTIKRKIVDEKMVVECKMNNIV

1OPA : CRQVFKK 133
 ++++K
4A60 : STRIYEK 153

**>1XCA**

PNFSGNWKIIRSENFEELLKVLGVNVMLRKIAVAAASKPAVEIKQEGDTFYIKTSTTVRTTEINFKVGEEFEEQTVDGRPCKSLVKWESENKMVCEQKLLKGEGPKTSWTMELTNDGELILTMTADDVVCTRVYVRE

**Similar protein structures from the Protein Data Bank:** [**148**](http://www.sbkb.org/kb/report.jsp?sid=s1322748059-80&pid=structures)

**Proteins with highly similar structures in PDB**

(<http://zhanglab.ccmb.med.umich.edu/I-TASSER/output/S88410/>)

**id = 2cbs_A I = 99% E = 4.7E-75 TM_Score : 0.992 RMSD 0.4**
1XCA : 1 PNFSGNWKIIRSENFEELLKVLGVNVMLRKIAVAAASKPAVEIKQEGDTFYIKTSTTVRT
 PNFSGNWKIIRSENFEELLKVLGVNVMLRKIAVAAASKPAVEIKQEGDTFYIKTSTTVRT
2CBS : 1 PNFSGNWKIIRSENFEELLKVLGVNVMLRKIAVAAASKPAVEIKQEGDTFYIKTSTTVRT

1XCA : TEINFKVGEEFEEQTVDGRPCKSLVKWESENKMVCEQKLLKGEGPKTSWTMELTNDGELI
 TEINFKVGEEFEEQTVDGRPCKSLVKWESENKMVCEQKLLKGEGPKTSWT ELTNDGELI
2CBS : TEINFKVGEEFEEQTVDGRPCKSLVKWESENKMVCEQKLLKGEGPKTSWTRELTNDGELI

1XCA : LTMTADDVVCTRVYVRE 137
 LTMTADDVVCTRVYVRE
2CBS : LTMTADDVVCTRVYVRE 137

**id = 2cbr_A I = 75% E = 1.6E-56 TM_Score : 0.982 RMSD 0.47**

1XCA : 1 PNFSGNWKIIRSENFEELLKVLGVNVMLRKIAVAAASKPAVEIKQEGDTFYIKTSTTVRT
 PNF+G WK+ SENF+ELLK LGVN MLRK+AVAAASKP VEI+Q+GD FYIKTSTTVRT
2CBR : 1 PNFAGTWKMRSSENFDELLKALGVNAMLRKVAVAAASKPHVEIRQDGDQFYIKTSTTVRT

1XCA : TEINFKVGEEFEEQTVDGRPCKSLVKWESENKMVCEQKLLKGEGPKTSWTMELTNDGELI
 TEINFKVGE FEE+TVDGR C+SL WE+ENK+ C Q LL+G+GPKT WT EL ND ELI
2CBR : TEINFKVGEGFEEETVDGRKCRSLPTWENENKIHCTQTLLEGDGPKTYWTRELAND-ELI

1XCA : LTMTADDVVCTRVYVRE 137
 LT ADDVVCTR+YVRE
2CBR : LTFGADDVVCTRIYVRE 136

**id = 1kqx_A I = 40% E = 2.5E-19 TM_Score : 0.883 RMSD 1.28**
1XCA : 1 PNFSGNWKIIRSENFEELLKVLGVNVMLRKIAVAAASKPAVEIKQEGDTFYIKTSTTVRT
 +F+G W+++ ++NFE+++K L ++ RKIAV K I Q GD F KT +T R
1KQX : 2 ADFNGTWEMLSNDNFEDVMKALDIDFATRKIAVHL--KQTKVIVQNGDKFETKTLSTFRN

1XCA : TEINFKVGEEFEEQT--VDGRPCKSLVKWESENKMVCEQKLLKGEGPKTSWTMELTNDGE
 E+NF +GEEF+EQT +D R K+LVKW+ + K+VC Q KGE W + D
1KQX : YEVNFVIGEEFDEQTKGLDNRTVKTLVKWDGD-KLVCVQ---KGEKENRGWKQWIEGD-L

1XCA : LILTMTADDVVCTRVY 134
 L L + D VC +V+
1KQX : LHLEIHCQDKVCHQVF 130

**id = 1lpj_A I = 38% E = 8.2E-19 TM_Score : 0.869 RMSD 1.48**
1XCA : 1 PNFSGNWKIIRSENFEELLKVLGVNVMLRKIAVAAASKPAVEIKQEGDTFYIKTSTTVRT
 + SG W ++ S+NFE + LG++ RKIA KP I+Q GD+F I T++++R
1LPJ : 2 ADLSGTWTLLSSDNFEGYMLALGIDFATRKIA--KLLKPQKVIEQNGDSFTIHTNSSLRN

1XCA : TEINFKVGEEFEEQT--VDGRPCKSLVKWESENKMVCEQKLLKGEGPKTSWTMELTNDGE
 + FKVGEEF+E +D R CKSLV W+++ ++ C Q KGE WT + D +
1LPJ : YFVKFKVGEEFDEDNRGLDNRKCKSLVIWDND-RLTCIQ---KGEKKNRGWTHWIEGD-K

1XCA : LILTMTADDVVCTRVYVR 136
 L L M + VC + + R
1LPJ : LHLEMFCEGQVCKQTFQR 132

**id = 1kgl_A I = 43% E = 4.4E-21 TM_Score : 0.866 RMSD 1.44**
1XCA : 2 NFSGNWKIIRSENFEELLKVLGVNVMLRKIAVAAASKPAVEIKQEGDTFYIKTSTTVRTT
 +F+G WK++ +ENFEE L+ L VNV LRKI A KP EI Q+GD I+T +T R
1KGL : 4 DFNGYWKMLSNENFEEYLRALDVNVALRKI--ANLLKPDKEIVQDGDHMIIRTLSTFRNY

1XCA : EINFKVGEEFEEQT--VDGRPCKSLVKWESENKMVCEQKLLKGEGPKTSWTMELTNDGEL
 ++F+VG+EFEE +D R C + V W+ +K+ C Q KGE WT + D EL
1KGL : IMDFQVGKEFEEDLTGIDDRKCMTTVSWDG-DKLQCVQ---KGEKEGRGWTQWIEGD-EL

1XCA : ILTMTADDVVCTRVY 134
 L M A+ V C +V+
1KGL : HLEMRAEGVTCKQVF 131

**id = 1opa_B I = 39% E = 3.0E-17 TM_Score : 0.862 RMSD 1.61**
1XCA : 4 SGNWKIIRSENFEELLKVLGVNVMLRKIAVAAASKPAVEIKQEGDTFYIKTSTTVRTTEI
 +G W++ +ENFE +K L ++ RKIAV I Q+GD F KT++T R ++
1OPA : 6 NGTWEMESNENFEGYMKALDIDFATRKIAVRLTQTKI--IVQDGDNFKTKTNSTFRNYDL

1XCA : NFKVGEEFEEQT--VDGRPCKSLVKWESENKMVCEQKLLKGEGPKTSWTMELTNDGELIL
 +F VG EF+E T +DGR K+LV WE N +VC Q KGE W + D +L L
1OPA : DFTVGVEFDEHTKGLDGRNVKTLVTWEG-NTLVCVQ---KGEKENRGWKQWVEGD-KLYL

1XCA : TMTADDVVCTRVY 134
 +T D VC +V+
1OPA : ELTCGDQVCRQVF 131

**id = 2wut_A I = 38% E = 4.9E-19 TM_Score : 0.858 RMSD 1.62**
1XCA : 3 FSGNWKIIRSENFEELLKVLGVNVMLRKIAVAAASKPAVEIKQEGDTFYIKTSTTVRTTE
 F G WK++ SENF++ +K LGV + RK+ A KP V I ++GD I+T +T + TE
2WUT : 6 FLGTWKLVSSENFDDYMKALGVGLATRKLGNLA--KPTVIISKKGDIITIRTESTFKNTE

1XCA : INFKVGEEFEEQTVDGRPCKSLVKWESENKMVCEQKLLKGEGPKTSWTMELTNDGELILT
 I+FK+G+EFEE T D R KS+V + + ++ + +G +T+ +L N G+++
2WUT : ISFKLGQEFEETTADNRKTKSIVTLQRGS----LNQVQRWDGKETTIKRKLVN-GKMVAE

1XCA : MTADDVVCTRVY 134
 VVCTR+Y
2WUT : CKMKGVVCTRIY 130

**id = 2rct_A I = 37% E = 2.5E-16 TM_Score : 0.856 RMSD 1.65**
1XCA : 4 SGNWKIIRSENFEELLKVLGVNVMLRKIAVAAASKPAVEIKQEGDTFYIKTSTTVRTTEI
 +G W++ +ENFE +K L ++ RKIAV ++ Q+GD F KT++T R ++
2RCT : 9 NGTWEMESNENFEGYMKALDIDFATRKIAVRLTQTKVID--QDGDNFKTKTTSTFRNYDV

1XCA : NFKVGEEFEEQT--VDGRPCKSLVKWESENKMVCEQKLLKGEGPKTSWTMELTNDGELIL
 +F VG EF+E T +D R K+LV WE + +VC Q KGE W + D +L L
2RCT : DFTVGVEFDEYTKSLDNRHVKALVTWEGD-VLVCVQ---KGEKENRGWKQWIEGD-KLYL

1XCA : TMTADDVVCTRVY 134
 +T D VC +V+
2RCT : ELTCGDQVCRQVF 134

**id = 1ggl_B I = 38% E = 3.6E-20 TM_Score : 0.850 RMSD 1.68**
1XCA : 1 PNFSGNWKIIRSENFEELLKVLGVNVMLRKIAVAAASKPAVEIKQEGDTFYIKTSTTVRT
 PN +G ++ + +N E+ L+ L +++ +RKIA+ KP EI+ +G+ ++T +T R
1GGL : 2 PNLTGYYRFVSQKNMEDYLQALNISLAVRKIALLL--KPDKEIEHQGNHMTVRTLSTFRN

1XCA : TEINFKVGEEFEE--QTVDGRPCKSLVKWESENKMVCEQKLLKGEGPKTSWTMELTNDGE
 + F VG EFEE ++VDGR C+++V WE E+ +VC Q KGE P W L +GE
1GGL : YTVQFDVGVEFEEDLRSVDGRKCQTIVTWEEEH-LVCVQ---KGEVPNRGWRHWL--EGE

1XCA : LI-LTMTADDVVCTRVY 134
 ++ L +TA D VC +V+
1GGL : MLYLELTARDAVCEQVF 130

**id = 2ans_B I = 37% E = 1.1E-14 TM_Score : 0.849 RMSD 1.72**
1XCA : 3 FSGNWKIIRSENFEELLKVLGVNVMLRKIAVAAASKPAVEIKQEGDTFYIKTSTTVRTTE
 F G WK++ SENF++ +K +GV RK VA +KP + I GD I++ +T + TE
2ANS : 4 FVGTWKLVSSENFDDYMKEVGVGFATRK--VAGMAKPNMIISVNGDLVTIRSESTFKNTE

1XCA : INFKVGEEFEEQTVDGRPCKSLVKWESENKMVCEQKLLKGEGPKTSWTMELTNDGE-LIL
 I+FK+G EF+E T D R KS++ + +V QK +G T T++ DG+ L++
2ANS : ISFKLGVEFDEITADDRKVKSIITLDG-GALVQVQKW---DGKST--TIKRKRDGDKLVV

1XCA : TMTADDVVCTRVYVR 136
 V TRVY R
2ANS : ECVMKGVTSTRVYER 130

**>2GIM**

METYTVKLGSDKGLLVFEPAKLTIKPGDTVEFLNNKVPPHNVVFDAALNPAKSADLAKSLSHKQLLMSPGQSTSTTFPADAPAGEYTFYCEPHRGAGMVGKITVAG

**Similar protein structures from the Protein Data Bank:** [**89**](http://www.sbkb.org/kb/report.jsp?sid=s1322747973-44&pid=structures)

**Proteins with highly similar structures in PDB**

**(**<http://zhanglab.ccmb.med.umich.edu/I-TASSER/output/S88446/>**)**

**id = 2w88_C I = 63% E = 8.0E-37 TM_Score : 0.981 RMSD 0.54**
2GIM : 1 METYTVKLGSDKGLLVFEPAKLTIKPGDTVEFLNNKVPPHNVVFDAALNPAKSADLAKSL
 MET+TVK+G+D GLL FEPA +T+ PGDTV+++NNK+PPHN++FD P S +LA L
2W88 : 1 METFTVKMGADSGLLQFEPANVTVHPGDTVKWVNNKLPPHNILFDDKQVPGASKELADKL

2GIM : SHKQLLMSPGQSTSTTFPADAPAGEYTFYCEPHRGAGMVGKITVAG 106
 SH QL+ SPG+S TF +D PA GYT+YC PHRGAGMVGKITV G
2W88 : SHSQLMFSPGESYEITFSSDFPAGTYTYYCAPHRGAGMVGKITVEG 106

**id = 1iuz_A I = 42% E = 1.2E-14 TM_Score : 0.884 RMSD 1.02**
2GIM : 6 VKLGSDKGLLVFEPAKLTIKPGDTVEFLNNKVPPHNVVFDAALNPAKSADLAKSLSHKQL
 VKLG D G L F P+K+++ G+ +EF+NN PHN+VFD PA A ++S+
1IUZ : 4 VKLGGDDGSLAFVPSKISVAAGEAIEFVNNAGFPHNIVFDEDAVPAGVD--ADAISYDDY

2GIM : LMSPGQSTSTTFPADAPAGEYTFYCEPHRGAGMVGKITV 104
 L S G++ + G Y YCEPH GAGM ITV
1IUZ : LNSKGETVVRKL---STPGVYGVYCEPHAGAGMKMTITV 97

**id = 1pcs_A I = 58% E = 8.4E-27 TM_Score : 0.879 RMSD 0.96**
2GIM : 5 TVKLGSDKGLLVFEPAKLTIKPGDTVEFLNNKVPPHNVVFDAALNPAKSADLAKSLSHKQ
 TVK+GSD G LVFEP+ +TIK G+ V+++NNK+ PHN+VFDA P AD A LSHK
1PCS : 4 TVKMGSDSGALVFEPSTVTIKAGEEVKWVNNKLSPHNIVFDADGVP---ADTAAKLSHKG

2GIM : LLMSPGQSTSTTFPADAPAGEYTFYCEPHRGAGMVGKITV 104
 LL + G+S ++TF G YT+YCEPHRGAGMVGK+ V
1PCS : LLFAAGESFTSTF---TEPGTYTYYCEPHRGAGMVGKVVV 97

**id = 7pcy_A I = 42% E = 3.2E-14 TM_Score : 0.878 RMSD 1.04**
2GIM : 6 VKLGSDKGLLVFEPAKLTIKPGDTVEFLNNKVPPHNVVFDAALNPAKSADLAKSLSHKQL
 VKLG D G L F P +T+ G+++EF+NN PHN+VFD PA A ++S +
7PCY : 4 VKLGGDDGSLAFVPNNITVGAGESIEFINNAGFPHNIVFDEDAVPAGVD--ADAISAEDY

2GIM : LMSPGQSTSTTFPADAPAGEYTFYCEPHRGAGMVGKITV 104
 L S GQ+ G Y YC+PH GAGM ITV
7PCY : LNSKGQTVVRKL---TTPGTYGVYCDPHSGAGMKMTITV 97

**id = 2plt_A I = 53% E = 3.5E-21 TM_Score : 0.878 RMSD 1.07**
2GIM : 5 TVKLGSDKGLLVFEPAKLTIKPGDTVEFLNNKVPPHNVVFDAALNPAKSADLAKSLSHKQ
 TVKLG+D G L F P LTIK G+TV F+NN PHN+VFD P S A ++S
2P1T : 3 TVKLGADSGALEFVPKTLTIKSGETVNFVNNAGFPHNIVFDEDAIP--SGVNADAISRDD

2GIM : LLMSPGQSTSTTFPADAPAGEYTFYCEPHRGAGMVGKITV 104
 L +PG++ S A AGEY +YCEPH+GAGMVGKI V
2P1T : YLNAPGETYSVKLTA---AGEYGYYCEPHQGAGMVGKIIV 97

**id = 6pcy_A I = 43% E = 2.2E-17 TM_Score : 0.872 RMSD 1.02**
2GIM : 6 VKLGSDKGLLVFEPAKLTIKPGDTVEFLNNKVPPHNVVFDAALNPAKSADLAKSLSHKQL
 V LG+D G L F P++ +I PG+ + F NN PHN+VFD P+ S+S + L
6PCY : 3 VLLGADDGSLAFVPSEFSISPGEKIVFKNNAGFPHNIVFDEDSIPSGVDASKISMSEEDL

2GIM : LMSPGQSTSTTFPADAPAGEYTFYCEPHRGAGMVGKITV 104
 L + G++ + GEY+FYC PH+GAGMVGK+TV
6PCY : LNAKGETFEVAL---SNKGEYSFYCSPHQGAGMVGKVTV 98

**id = 1byo_B I = 46% E = 4.9E-15 TM_Score : 0.862 RMSD 1.14**
2GIM : 6 VKLGSDKGLLVFEPAKLTIKPGDTVEFLNNKVPPHNVVFDAALNPAKSADLAK-SLSHKQ
 V LGS G L F P+ L+I G+ + F NN PHN +FD PA D+ K S+ +
1BYO : 3 VLLGSSDGGLAFVPSDLSIASGEKITFKNNAGFPHNDLFDEDEVPA-GVDVTKISMPEED

2GIM : LLMSPGQSTSTTFPADAPAGEYTFYCEPHRGAGMVGKITV 104
 LL +PG+ S T G Y FYC PH GAGMVGK+TV
1BYO : LLNAPGEEYSVTL---TEKGTYKFYCAPHAGAGMVGKVTV 98

**id = 1oow_A I = 45% E = 1.0E-15 TM_Score : 0.858 RMSD 1.18**
2GIM : 6 VKLGSDKGLLVFEPAKLTIKPGDTVEFLNNKVPPHNVVFDAALNPAKSADLAK-SLSHKQ
 V LG D G F P ++ G+ + F NN PHNVVFD P+ D AK S+S +
1OOW : 3 VLLGGDDGSEAFLPGDFSVASGEEIVFKNNAGFPHNVVFDEDEIPS-GVDAAKISMSEED

2GIM : LLMSPGQSTSTTFPADAPAGEYTFYCEPHRGAGMVGKITV 104
 LL +PG++ T G Y FYC PH+GAGMVGK+TV
1OOW : LLNAPGETYKVTL---TEKGTYKFYCSPHQGAGMVGKVTV 98

**id = 1bxu_A I = 48% E = 4.3E-23 TM_Score : 0.826 RMSD 0.78**
2GIM : 2 ETYTVKLGSDKGLLVFEPAKLTIKPGDTVEFLNNKVPPHNVVFDAALNPAKSADLAKSLS
 +T +K+G+D G+L FEP+ + I+ GDTV+++NNK+ PHNVV + LS
1BXU : 1 QTVAIKMGADNGMLAFEPSTIEIQAGDTVQWVNNKLAPHNVVVEG----------QPELS

2GIM : HKQLLMSPGQSTSTTFPADAPAGEYTFYCEPHRGAGMVGKITV 104
 HK L SPG++ TF + G YT+YCEPHRGAGMVGKI V
1BXU : HKDLAFSPGETFEATF---SEPGTYTYYCEPHRGAGMVGKIVV 90

**>1SPD**

CATKAVCVLKGDGPVQGIINFEQKESNGPVKVWGSIKGLTEGLHGFHVHEFGDNTAGCTSAGPHFNPLSRKHGGPKDEERHVGDLGNVTADKDGVADVSIEDSVISLSGDHCIIGRTLVVHEKADDLGKGGNEESTKTGNAGSRLACGVIGIAQ

**Similar protein structures from the Protein Data Bank:** [**152**](http://www.sbkb.org/kb/report.jsp?sid=s1322748353-96&pid=structures)

**Proteins with highly similar structures in PDB**

(<http://zhanglab.ccmb.med.umich.edu/I-TASSER/output/S88448/>)

**id = 1azv_B I = 99% E = 3.7E-83 TM_Score : 0.985 RMSD 0.44**
1SPD : 2 ATKAVCVLKGDGPVQGIINFEQKESNGPVKVWGSIKGLTEGLHGFHVHEFGDNTAGCTSA
 ATKAVCVLKGDGPVQGIINFEQKESNGPVKVWGSIK LTEGLHGFHVHEFGDNTAGCTSA
1AZV : 1 ATKAVCVLKGDGPVQGIINFEQKESNGPVKVWGSIKRLTEGLHGFHVHEFGDNTAGCTSA

1SPD : GPHFNPLSRKHGGPKDEERHVGDLGNVTADKDGVADVSIEDSVISLSGDHCIIGRTLVVH
 GPHFNPLSRKHGGPKDEERHVGDLGNVTADKDGVADVSIEDSVISLSGDHCIIGRTLVVH
1AZV : GPHFNPLSRKHGGPKDEERHVGDLGNVTADKDGVADVSIEDSVISLSGDHCIIGRTLVVH

1SPD : EKADDLGKGGNEESTKTGNAGSRLACGVIGIAQ 154
 EKADDLGKGGNEESTKTGNAGSRLACGVIGIAQ
1AZV : EKADDLGKGGNEESTKTGNAGSRLACGVIGIAQ 153

**id = 1to4_D I = 60% E = 2.5E-47 TM_Score : 0.975 RMSD 0.87**
1SPD : 4 KAVCVLKGDGPVQGIINFEQKESNGPVKVWGSIKGLTEGLHGFHVHEFGDNTAGCTSAGP
 KAVCV+ G V+G++ F Q+ NGPV V GL G HGFHVHEFGD T GCTSAG
1TO4 : 5 KAVCVMTGTAGVKGVVKFTQETDNGPVHVHAEFSGLKAGKHGFHVHEFGDTTNGCTSAGA

1SPD : HFNPLSRKHGGPKDEERHVGDLGNVTADKDGVADVSIEDSVISLSGDHCIIGRTLVVHEK
 HFNP ++HG P+D RHVGDLGNV A DG A + D +ISL+G H IIGR++V+HE
1TO4 : HFNPTKQEHGAPEDSIRHVGDLGNVVAGADGNAVYNATDKLISLNGSHSIIGRSMVIHEN

1SPD : ADDLGKGGNEESTKTGNAGSRLACGVIGIA 153
 DDLG+GG+E S TGNAG RLACGV+G+A
1TO4 : EDDLGRGGHELSKVTGNAGGRLACGVVGLA 154

**id = 3km2_X I = 57% E = 4.5E-43 TM_Score : 0.972 RMSD 0.72**
1SPD : 4 KAVCVLKGDGPVQGIINFEQKESNGPVKVWGSIKGLTEGLHGFHVHEFGDNTAGCTSAGP
 KAV VLKG+ V+G++ Q + +GP V I GL GLHGFH+HE+GD T GC S G
3KM2 : 4 KAVAVLKGNSNVEGVVTLSQ-DDDGPTTVNVRITGLAPGLHGFHLHEYGDTTNGCMSTGA

1SPD : HFNPLSRKHGGPKDEERHVGDLGNVTADKDGVADVSIEDSVISLSGDHCIIGRTLVVHEK
 HFNP HG P DE RH GDLGN+ A+ DGVA+V++ D+ I L+G + ++GR LVVHE
3KM2 : HFNPNKLTHGAPGDEIRHAGDLGNIVANADGVAEVTLVDNQIPLTGPNSVVGRALVVHEL

1SPD : ADDLGKGGNEESTKTGNAGSRLACGVIGI 152
 DDLGKGG+E S TGNAG RLACGV+G+
3KM2 : EDDLGKGGHELSLTTGNAGGRLACGVVGL 151

**id = 2jcw_A I = 56% E = 4.7E-42 TM_Score : 0.967 RMSD 0.69**
1SPD : 4 KAVCVLKGDGPVQGIINFEQKESNGPVKVWGSIKGLTEGL-HGFHVHEFGDNTAGCTSAG
 +AV VLKGD V G++ FEQ + P V I G + GFH+HEFGD T GC SAG
2JCW : 2 QAVAVLKGDAGVSGVVKFEQASESEPTTVSYEIAGNSPNAERGFHIHEFGDATNGCVSAG

1SPD : PHFNPLSRKHGGPKDEERHVGDLGNVTADKDGVADVSIEDSVISLSGDHCIIGRTLVVHE
 PHFNP + HG P DE RHVGD+GNV D++GVA S +DS+I L G ++GR++V+H
2JCW : PHFNPFKKTHGAPTDEVRHVGDMGNVKTDENGVAKGSFKDSLIKLIGPTSVVGRSVVIHA

1SPD : KADDLGKGGNEESTKTGNAGSRLACGVIGI 152
 DDLGKG EES KTGNAG R ACGVIG+
2JCW : GQDDLGKGDTEESLKTGNAGPRPACGVIGL 151

**id = 1srd_D I = 58% E = 3.2E-44 TM_Score : 0.967 RMSD 0.80**
1SPD : 4 KAVCVLKGDGPVQGIINFEQKESNGPVKVWGSIKGLTEGLHGFHVHEFGDNTAGCTSAGP
 KAV VLKG V+G++ Q E +GP V I GL G HGFH+HEFGD T GC S GP
1SRD : 4 KAVAVLKGTSNVEGVVTLTQ-EDDGPTTVNVRISGLAPGKHGFHLHEFGDTTNGCMSTGP

1SPD : HFNPLSRKHGGPKDEERHVGDLGNVTADKDGVADVSIEDSVISLSGDHCIIGRTLVVHEK
 HFNP + HG P+DE RH GDLGN+ A+ DGVA+ +I D+ I L+G + ++GR LVVHE
1SRD : HFNPDKKTHGAPEDEVRHAGDLGNIVANTDGVAEATIVDNQIPLTGPNSVVGRALVVHEL

1SPD : ADDLGKGGNEESTKTGNAGSRLACGVIGI 152
 DDLGKGG+E S TGNAG RLACGV+G+
1SRD : EDDLGKGGHELSPTTGNAGGRLACGVVGL 151

**id = 1cb4_B I = 81% E = 2.4E-64 TM_Score : 0.965 RMSD 0.74**
1SPD : 2 ATKAVCVLKGDGPVQGIINFEQKESNGPVKVWGSIKGLTEGLHGFHVHEFGDNTAGCTSA
 ATKAVCVLKGDGPVQG I+FE K V V GSI GLTEG HGFHVH+FGDNT GCTSA
1SRD : 1 ATKAVCVLKGDGPVQGTIHFEAKGDT--VVVTGSITGLTEGDHGFHVHQFGDNTQGCTSA

1SPD : GPHFNPLSRKHGGPKDEERHVGDLGNVTADKDGVADVSIEDSVISLSGDHCIIGRTLVVH
 GPHFNPLS+KHGGPKD+ERHVGDLGNVTADK+GVA V I D +ISLSG++ IIGRT+VVH
1SRD : GPHFNPLSKKHGGPKDDERHVGDLGNVTADKNGVAIVDIVDPLISLSGEYSIIGRTMVVH

1SPD : EKADDLGKGGNEESTKTGNAGSRLACGVIGIAQ 154
 EK DDLG+GGNEESTKTGNAGSRLACGVIGIA+
1SRD : EKPDDLGRGGNEESTKTGNAGSRLACGVIGIAK 151

**id = 1xso_B I = 67% E = 1.1E-50 TM_Score : 0.965 RMSD 0.47**
1SPD : 3 TKAVCVLKGDGPVQGIINFEQKESNGPVKVWGSIKGLTEGLHGFHVHEFGDNTAGCTSAG
 KAVCVL G G V+G+++FEQ++ G V V G I+GLT+GLHGFH+H FGDNT GC SAG
1SRD : 1 VKAVCVLAGSGDVKGVVHFEQQDE-GAVSVEGKIEGLTDGLHGFHIHVFGDNTNGCMSAG

1SPD : PHFNPLSRKHGGPKDEERHVGDLGNVTADKDGVADVSIEDSVISLSGDHCIIGRTLVVHE
 HFNP ++ HG P D +RHVGDLGNVTA+ GVA I DS+ISL G + IIGRT VVHE
1SRD : SHFNPENKNHGAPGDTDRHVGDLGNVTAEG-GVAQFKITDSLISLKGPNSIIGRTAVVHE

1SPD : KADDLGKGGNEESTKTGNAGSRLACGVIGIA 153
 KADDLGKGGN+ES KTGNAG RLACGVIG +
1SRD : KADDLGKGGNDESLKTGNAGGRLACGVIGYS 149

**id = 2q2l_B I = 57% E = 1.0E-41 TM_Score : 0.961 RMSD 0.79**
1SPD : 4 KAVCVLKGDGPVQGIINFEQKESNGPVKVWGSIKGLTEGLHGFHVHEFGDNTAGCTSAGP
 K V VL V G I F Q E +GP V G+I GL GLHGFHVH GD T GC S GP
1SRD : 3 KGVAVLSSSEGVAGTILFTQ-EGDGPTTVTGNISGLKPGLHGFHVHALGDTTNGCMSTGP

1SPD : HFNPLSRKHGGPKDEERHVGDLGNVTADKDGVADVSIEDSVISLSGDHCIIGRTLVVHEK
 HFNP ++HG P+DE RH GDLGN+T DG A +I D I L+G H IIGR +VVH
1SRD : HFNPAGKEHGSPEDETRHAGDLGNITVGDDGTACFTIVDKQIPLTGPHSIIGRAVVVHAD

1SPD : ADDLGKGGNEESTKTGNAGSRLACGVIGI 152
 DDLGKGG+E S TGNAG R+ACG+IG+
1SRD : PDDLGKGGHELSKSTGNAGGRIACGIIGL 150

**id = 3l9e_D I = 63% E = 7.6E-47 TM_Score : 0.961 RMSD 0.84**
1SPD : 4 KAVCVLKGDGPVQGIINFEQKESNGPVKVWGSIKGLTEGLHGFHVHEFGDNTAGCTSAGP
 KAVCVL+GD V G + F+Q++ PV V G ++GLT+G HGFHVHEFGDNT GCTSAG
1SRD : 4 KAVCVLRGD--VSGTVFFDQQDEKSPVVVSGEVQGLTKGKHGFHVHEFGDNTNGCTSAGA

1SPD : HFNPLSRKHGGPKDEERHVGDLGNVTADKD-GVADVSIEDSVISLSGDHCIIGRTLVVHE
 HFNP + HGGP RHVGDLGN+ A +D GV VSI+DS ISL G + IIGRTLVVH
1SRD : HFNPEKQDHGGPSSAVRHVGDLGNIEAIEDSGVTKVSIQDSQISLHGPNSIIGRTLVVHA

1SPD : KADDLGKGGNEESTKTGNAGSRLACGVIGIAQ 154
 DDLG GG+E S TGNAG R+ACGVIG+A+
1SRD : DPDDLGLGGHELSKTTGNAGGRIACGVIGLAK 153

**id = 3f7k_A I = 62% E = 2.3E-46 TM_Score : 0.961 RMSD 0.76**
1SPD : 2 ATKAVCVLKGDGPVQGIINFEQKESNGPVKVWGSIKGLTEGLHGFHVHEFGDNTAGCTSA
 A AVCVLKGD PV G I+ KE V V G I GLT G HGFHVHEFGDNT GCTSA
1SRD : 1 AIHAVCVLKGDSPVTGTIHL--KEEGDMVTVTGEITGLTPGKHGFHVHEFGDNTNGCTSA

1SPD : GPHFNPLSRKHGGPKDEERHVGDLGNVTADKDGVADVSIEDSVISLSGDHCIIGRTLVVH
 G HFNP ++HG P+DE RH GDLGNV A +DG A ++++D ++ L+G +IGRTLVVH
1SRD : GGHFNPHGKEHGAPEDENRHAGDLGNVVAGEDGKAVINMKDKLVKLTGPDSVIGRTLVVH

1SPD : EKADDLGKGGNEESTKTGNAGSRLACGVIGIAQ 154
 DDLG+GG+E+S TGNAG RLACGVIGI +
1SRD : VDEDDLGRGGHEQSKITGNAGGRLACGVIGITK 151

**>1J2A**

AKGDPHVLLTTSAGNIELELDKQKAPVSVQNFVDYVNSGFYNNTTFHRVIPGFMIQGGGFTEQMQQKKPNPPIKNEADNGLRNTRGTIAMARTADKDSATSQFFINVADNAFLDHGQRDFGYAVFGKVVKGMDVADKISQVPTHDVGPYQNVPSKPVVILSATVLP

**Similar protein structures from the Protein Data Bank:** [**136**](http://www.sbkb.org/kb/report.jsp?sid=s1322808419-43&pid=structures)

**Proteins with highly similar structures in PDB**

(<http://zhanglab.ccmb.med.umich.edu/I-TASSER/output/S88509/>)

**id = 1lop_A I = 55% E = 1.8E-43 TM_Score : 0.957 RMSD 0.62**
1J2A : 7 VLLTTSAGNIELELDKQKAPVSVQNFVDYVNSGFYNNTTFHRVIPGFMIQGGGFTEQMQQ
 V T+ G+I ++ KAP +V+NF+DY GFYNNT FHRVI GFMIQGGGF M+Q
1LOP : 2 VTFHTNHGDIVIKTFDDKAPETVKNFLDYCREGFYNNTIFHRVINGFMIQGGGFEPGMKQ

1J2A : KKPNPPIKNEADNGLRNTRGTIAMARTADKDSATSQFFINVADNAFLD---HGQRDFGYA
 K PIKNEA+NGL+NTRGT+AMART SAT+QFFINV DN FL+ + +GY
1LOP : KATKEPIKNEANNGLKNTRGTLAMARTQAPHSATAQFFINVVDNDFLNFSGESLQGWGYC

1J2A : VFGKVVKGMDVADKISQVPTHDVGPYQNVPSKPVVILSATV 164
 VF +VV GMD DKI V T G +Q+VP + V+I S TV
1LOP : VFAEVVDGMDEVDKIKGVATGRSGMHQDVPKEDVIIESVTV 162

**id = 3s6m_A I = 56% E = 1.4E-43 TM_Score : 0.943 RMSD 0.82**
1J2A : 7 VLLTTSAGNIELELDKQKAPVSVQNFVDYVNSGFYNNTTFHRVIPGFMIQGGGFTEQMQQ
 V L T+ G I+LELD+ KAP +V+NF++YV G Y+ T FHRVI GFMIQGGGF ++Q
3S6M : 6 VELHTNHGVIKLELDEAKAPKTVENFLNYVKKGHYDGTIFHRVINGFMIQGGGFEPGLKQ

1J2A : KKPNPPIKNEADNGLRNTRGTIAMARTADKDSATSQFFINVADNAFLDHGQ---RDFGYA
 K + PI NEA+NGL+N TIAMART D SAT+QFFINV DN FL+H + +GYA
3S6M : KPTDAPIANEANNGLKNDTYTIAMARTNDPHSATAQFFINVNDNEFLNHSSPTPQGWGYA

1J2A : VFGKVVKGMDVADKISQVPTHDVGPYQNVPSKPVVILSATVL 165
 VFGKVV+G D+ DKI V T G +Q+VP+ VVI A V+
3S6M : VFGKVVEGQDIVDKIKAVKTGSKGFHQDVPNDDVVIEKAVVV 167

**id = 2oju_B I = 31% E = 8.8E-14 TM_Score : 0.867 RMSD 1.87**
1J2A : 3 GDPHVLLTTSAGNIELELDKQKAPVSVQNFVDYVNSGFYNNTTFHRVIPGFMIQGGGFT-
 G V L T G+I++E+ ++ P + +NF+ S +YN FHR I GFM+Q G T
2OJU : 5 GGMSVTLHTDVGDIKIEVFCERTPKTCENFLALCASNYYNGCIFHRNIKGFMVQTGDPTG

1J2A : -----EQMQQKKPNPPIKNEADNGLR-NTRGTIAMARTADKDSATSQFFINVADNAFLDH
 + KK ++E L+ N RG ++MA ++ SQFFI L
2OJU : TGRGGNSIWGKK----FEDEYSEYLKHNVRGVVSMANNG-PNTNGSQFFITYGKQPHL--

1J2A : GQRDFGYAVFGKVVKGMDVADKISQVPTHD--VGPYQNVPSKPVVI 159
 D Y VFGKV+ G++ D++ ++P ++ P +V K + I
2OJU : ---DMKYTVFGKVIDGLETLDELEKLPVNEKTYRPLNDVHIKDITI 160

**id = 1zkc_B I = 32% E = 3.4E-13 TM_Score : 0.862 RMSD 1.85**
1J2A : 2 KGDPHVLLTTSAGNIELELDKQKAPVSVQNFVDYVNSGFYNNTTFHRVIPGFMIQGGGFT
 +G +V L T+ G++ LEL P + +NF+ +Y+ T FHR I F+IQGG T
1ZKC : 17 RGSGYVRLHTNKGDLNLELHCDLTPKTCENFIRLCKKHYYDGTIFHRSIRNFVIQGGDPT

1J2A : EQMQQKKP--NPPIKNEADNGLRNT-RGTIAMARTADKDSATSQFFINVADNAFLDHGQR
 + P K+E L +T RG ++MA + +S SQFFI A+LD
1ZKC : GTGTGGESYWGKPFKDEFRPNLSHTGRGILSMANSG-PNSNRSQFFITFRSCAYLDK---

1J2A : DFGYAVFGKVVKGMDVADKISQVPTHDVGPYQNVPSKPVVILSATVL 165
 + +FG+VV G DV + V + P + P + + I + TV
1ZKC : --KHTIFGRVVGGFDVLTAMENVES---DPKTDRPKEEIRIDATTVF 174

**id = 3bo7_D I = 28% E = 1.8E-10 TM_Score : 0.862 RMSD 1.46**
1J2A : 2 KGDPHVLLTTSAGNIELELDKQKAPVSVQNFVDYVNSGFYNNTTFHRVIPGFMIQGGGFT
 K ++ + T+ G++ +EL AP + +F+ ++++T FHR I FMIQGG
3BO7 : 4 KKKGYLRIVTTQGSLNIELHADMAPRACDSFLRLCAVKYFDDTIFHRCIRNFMIQGGRAE

1J2A : EQMQQKKPN--------------PPIKNEADNGL-RNTRGTIAMARTADKDSATSQFFIN
 + KK P ++E DN L G ++MA K S S+FFI
3BO7 : LRQPSKKKEVQQSPRSISGFPGGAPFEDEFDNRLVHQGIGVLSMANDG-KHSNLSEFFIT

1J2A : VADNAFLDHGQRDFGYAVFGKVVKGMDVADKISQVPT 143
 L++ + +FG+VV G+DV + ++ T
3BO7 : FKSCEHLNN-----KHTIFGRVVGGLDVLRQWEKLET 154

**id = 1w74_B I = 31% E = 1.4E-06 TM_Score : 0.857 RMSD 1.73**
1J2A : 9 LTTSAGNIELELDKQKAPVSVQNFV-------DYVN-------SG-FYNNTTFHRVIPGF
 L T+ G+I++ L AP +V NFV DY SG FY+ FHRVI GF
1W74 : 28 LHTNRGDIKIALFGNHAPKTVANFVGLAQGTKDYSTQNASGGPSGPFYDGAVFHRVIQGF

1J2A : MIQGGGFTEQMQQKKPNPPIKNEADNGLRNTRGTIAMARTADKDSATSQFFINVADNAFL
 MIQGG T + +E L+ + + A + SQFFI V L
1W74 : MIQGGDPTGTGRGGPGY-KFADEFHPELQFDKPYLLAMANAGPGTNGSQFFITVGKTPHL

1J2A : DHGQRDFGYAVFGKVV--KGMDVADKISQVPTHDVGPYQNVPSKPVVILSATV 164
 + + +FG+V+ + V + IS+ T + P+ PVVI S T+
1W74 : NR-----RHTIFGEVIDAESQRVVEAISKTATDG----NDRPTDPVVIESITI 190

**id = 1c5f_O I = 32% E = 7.0E-08 TM_Score : 0.856 RMSD 2.02**
1J2A : 13 AGNIELELDKQKAPVSVQNFVDYVN--------SG---FYNNTTFHRVIPGFMIQGGGFT
 AG I +EL AP + NF+ SG Y +TFHRVI FMIQGG FT
1C5F : 20 AGRIVMELYNDIAPRTCNNFLMLCTGMAGTGKISGKPLHYKGSTFHRVIKNFMIQGGDFT

1J2A : -----------------EQMQQKKPNPPIKNEADNGLRNTRGTIAMARTADKDSATSQFF
 E+ K P + + A+ G NT G SQFF
1C5F : KGDGTGGESIYGGMFDDEEFVMKHDEPFVVSMANKG-PNTNG--------------SQFF

1J2A : INVADNAFLDHGQRDFGYAVFGKVVKGMDVADKISQVPTHDVGPYQNVPSKPVVILSATV
 I L++ + VFGKVV G +V KI + T+ +N P VVIL+
1C5F : ITTTPAPHLNN-----IHVVFGKVVSGQEVVTKIEYLKTNS----KNRPLADVVILNCGE

1J2A : L 165
 L
1C5F : L 176

**id = 2poe_A I = 30% E = 2.6E-10 TM_Score : 0.856 RMSD 1.71**
1J2A : 7 VLLTTSAGNIELELDKQKAPVSVQNFVDYVNSGFYNNTTFHRVIPGFMIQGGGFT-----
 V + T+ G+++ EL + P + +NF+ SG+Y NT FH+ I GF+IQGG T
2POE : 19 VRIITNYGDLKFELFCSQCPKACKNFLALSASGYYKNTIFHKNIKGFIIQGGDPTGTGKG

1J2A : -EQMQQKKPNPPIKNEADNGLRNTRGTIAMA-RTADKDSAT--SQFFINVADNAFLDHGQ
 E + + + I E + RG ++MA + A K T SQFFI + Q
2POE : GESIYGRYFDDEIYPELK---YDRRGILSMASKGASKKPNTNGSQFFITYSSLP-----Q

1J2A : RDFGYAVFGKVVKGMDVADKISQVPTHDV-GPYQNVPSKPVVILS 161
 + Y +FGK++ G + + + P+ P + K +VI S
2POE : LNGEYVIFGKLIDGFETLNTLENCPSDKSHKPIDEIIIKDIVIHS 175

**id = 1z81_A I = 24% E = 8.3E-06 TM_Score : 0.855 RMSD 1.97**
1J2A : 4 DPHVLLTTSAGN-----IELELDKQKAPVSVQNFVDYVNSGF--------YNNTTFHRVI
 +P V + + GN + EL + P + +NF + + Y NT FHRVI
1Z81 : 59 NPVVFMDINLGNNFLGKFKFELFQNIVPKTSENFRQFCTGEYKVNNLPVGYKNTIFHRVI

1J2A : PGFMIQGGGFTEQMQQKKPN---PPIKNEADNGLRNTRGTIAMARTADKDSATSQFFINV
 FMIQGG F + +E + + G ++MA + ++ QFFI
1Z81 : KEFMIQGGDFINHNGSGSLSIYGEKFDDENFDIKHDKEGLLSMANSG-PNTNGCQFFITT

1J2A : ADNAFLDHGQRDFGYAVFGKVVKGMDVADKISQVPTHDVGPYQNVPSKPVVILSATVL 165
 +LD VFG+++ D + ++ V PY P P+ ++ L
1Z81 : KKCEWLDGKN-----VVFGRIIDN-DSLLLLKKIENVSVTPYIYKPKIPINVVECGEL 229

**>1IAZ** SADVAGAVIDGASLSFDILKTVLEALGNVKRKIAVGVDNESGKTWTALNTYFRSGTSDIVLPHKVPHGKALLYNGQKDRGPVATGAVGVLAYLMSDGNTLAVLFSVPYDYNWYSNWWNVRIYKGKRRADQRMYEELYYNLSPFRGDNGWHTRNLGYGLKSRGFMNSSGHAILEIHVSKA

**Similar protein structures from the Protein Data Bank:** [**10**](http://sbkb.org/kb/report.jsp?sid=s1322809287-17&pid=structures)

**Proteins with highly similar structures in PDB**

(<http://zhanglab.ccmb.med.umich.edu/I-TASSER/output/S88527/>)

**id = 3lim_F I = 89% E = 5.2E-93 TM_Score : 0.978 RMSD 0.92**
1IAZ : 2 ADVAGAVIDGASLSFDILKTVLEALGNVKRKIAVGVDNESGKTWTALNTYFRSGTSDIVL
 ADVAGAVIDGA L FD+LKTVLEALGNVKRKIAVG+DNESGKTWTA+NTYFRSGTSDIVL
3LIM : 1 ADVAGAVIDGAGLGFDVLKTVLEALGNVKRKIAVGIDNESGKTWTAMNTYFRSGTSDIVL

1IAZ : PHKVPHGKALLYNGQKDRGPVATGAVGVLAYLMSDGNTLAVLFSVPYDYNWYSNWWNVRI
 PHKV HGKALLYNGQK+RGPVATG VGV+AY MSDGNTLAVLFSVPYDYNWYSNWWNVR+
3LIM : PHKVAHGKALLYNGQKNRGPVATGVVGVIAYSMSDGNTLAVLFSVPYDYNWYSNWWNVRV

1IAZ : YKGKRRADQRMYEELYYNLSPFRGDNGWHTRNLGYGLKSRGFMNSSGHAILEIHVSKA 179
 YKG++RADQRMYEELYY+ SPFRGDNGWH+R LGYGLKSRGFMNSSGHAILEIHV+KA
3LIM : YKGQKRADQRMYEELYYHRSPFRGDNGWHSRGLGYGLKSRGFMNSSGHAILEIHVTKA 178

**id = 1gwy_B I = 67% E = 7.9E-69 TM_Score : 0.967 RMSD 0.53**
1IAZ : 4 VAGAVIDGASLSFDILKTVLEALGNVKRKIAVGVDNESGKTWTALNTYFRSGTSDIVLPH
 +AG +I GASL+F +L VLE LG V RKIAVG+DNESG TWTALN YFRSGT+D++LP
1GWY : 2 LAGTIIAGASLTFQVLDKVLEELGKVSRKIAVGIDNESGGTWTALNAYFRSGTTDVILPE

1IAZ : KVPHGKALLYNGQKDRGPVATGAVGVLAYLMSDGNTLAVLFSVPYDYNWYSNWWNVRIYK
 VP+ KALLY+G+KD GPVATGAV AY MS GNTL V+FSVP+DYNWYSNWW+V+IY
1GWY : FVPNTKALLYSGRKDTGPVATGAVAAFAYYMSSGNTLGVMFSVPFDYNWYSNWWDVKIYS

1IAZ : GKRRADQRMYEELYYNLSPFRGDNGWHTRNLGYGLKSRGFMNSSGHAILEIHVSK 178
 GKRRADQ MYE+LYY +P+RGDNGWH +NLGYGL+ +G M S+G A ++I +S+
1GWY : GKRRADQGMYEDLYYG-NPYRGDNGWHEKNLGYGLRMKGIMTSAGEAKMQIKISR 175

>**1WHO**

VPKVTFTVEKGSNEKHLAVLVKYEGDTMAEVELREHGSDEWVAMTKGEGGVWTFDSEEPLQGPFNFRFLTEKGM

KNVFDDVVPEKYTIGATYAPEE

**Similar protein structures from the Protein Data Bank:** [**9**](http://sbkb.org/kb/report.jsp?sid=s1322809429-62&pid=structures)

**Proteins with highly similar structures in PDB**

**(**<http://zhanglab.ccmb.med.umich.edu/I-TASSER/output/S88528/>**)**

**id = 3ft1_D I = 57% E = 1.2E-26 TM_Score : 0.914 RMSD 1.14**
1WHO : 3 KVTFTVEKGSNEKHLAVLVKYE--GDTMAEVELREHGSDEWVAMTKGEGGVWTFDSEEPL
 +VTFTV+KGS+ K L + +KY GD++AEVELR+HGS+EW +TK +G VW S +PL
3FT1 : 3 QVTFTVQKGSDPKKLVLDIKYTRPGDSLAEVELRQHGSEEWEPLTK-KGNVWEVKSSKPL

1WHO : QGPFNFRFLTEKGMKNVFDDVVPEKYTIGATYAPEE 96
 GPFNFRF+++ GM+NVFD+V+P ++IG TY PEE
3FT1 : VGPFNFRFMSKGGMRNVFDEVIPTAFSIGKTYKPEE 97

**id = 2jnz_A I = 57% E = 1.0E-26 TM_Score : 0.905 RMSD 1.22**
1WHO : 3 KVTFTVEKGSNEKHLAVLVKYE--GDTMAEVELREHGSDEWVAMTKGEGGVWTFDSEEPL
 +VTFTV+KGS+ K L + +KY GD++AEVELR+HGS+EW +TK +G VW S +PL
2JNZ : 14 QVTFTVQKGSDPKKLVLDIKYTRPGDSLAEVELRQHGSEEWEPLTK-KGNVWEVKSSKPL

1WHO : QGPFNFRFLTEKGMKNVFDDVVPEKYTIGATYAPEE 96
 GPFNFRF+++ GM+NVFD+V+P ++IG TY PEE
2JNZ : VGPFNFRFMSKGGMRNVFDEVIPTAFSIGKTYKPEE 108

**id = 1n10_A I = 44% E = 2.7E-16 TM_Score : 0.856 RMSD 1.78**1WHO : 3 KVTFTVEKGSNEKHLAVLVKY---EGDTMAEVELREHGSDEWVAMTKGEGGVWTFDSEEP
 KVTF VEKGSN +LA+LVKY +GD +A V+++E G D+W+ + + G +W D+ +
1N10 : 147 KVTFHVEKGSNPNYLALLVKYVNGDGDVVA-VDIKEKGKDKWIELKESWGAIWRIDTPDK

1WHO : LQGPFNFRFLTEKGMKNVFDDVVPEKYTIGATY 92
 L GPF R+ TE G K +DV+PE + +Y
1N10 : LTGPFTVRYTTEGGTKTEAEDVIPEGWKADTSY 238

**id = 2hcz_X I = 38% E = 2.0E-13 TM_Score : 0.848 RMSD 2.03**
1WHO : 3 KVTFTVEKGSNEKHLAVLVKYEGDT--MAEVELREHGSDEWVAMTKGEGGVWTFDSEEPL
 K+ F +EKG N +LAVLVKY D + +E+++ S EW M G +W D+ + L
2HCZ : 147 KIVFHIEKGCNPNYLAVLVKYVADDGDIVLMEIQDKLSAEWKPMKLSWGAIWRMDTAKAL

1WHO : QGPFNFRFLTEKGMKNVFDDVVPEKYTIGATY 92
 +GPF+ R +E G K + DV+P + A Y
2HCZ : KGPFSIRLTSESGKKVIAKDVIPANWRPDAVY 238

**α/β class of proteins**

| ***X-ray PDB*** | ***NMR PDB*** | ***Region of Structural Variation*** | ***Link to TASSER results*** |
| --- | --- | --- | --- |
| **1RRF** | 1U81 | 18: 24 - MRILMVG  43: 48 - VTTIPT  76: 92 - PLWRFQNTQGLIFVV  99: 113- RVNEAREELMRMLAE | <http://zhanglab.ccmb.med.umich.edu/I-TASSER/output/S89131> |
| **1EZ9** | 1EZO | 106: 112- YPIAVEA  115: 117- LIY  145: 147- SAL  222: 227- TAMTIN  258: 266- FVGVLSAGI  305: 311- KSYEEEL | <http://zhanglab.ccmb.med.umich.edu/I-TASSER/output/S89132/> |
| **5P21** | 1CRP | 37: 46 - EDSYRKQVVI  49: 58 - ETCLLDILDT  141: 144- YIET | <http://zhanglab.ccmb.med.umich.edu/I-TASSER/output/S89133/> |

>**1RRF**

MGNIFANLFKGLFGKKEMRILMVGLDAAGKTTILYKLKLGEIVTTIPTIGFNVETVEYKNISFTVWDVGGQDKIRPLWRHYFQNTQGLIFVVDSNDRERVNEAREELMRMLAEDELRDAVLLVFANKQDLPNAMNAAEITDKLGLHSLRHRNWYIQATCATSGDGLYEGLDWLSNQLRNQK

**Similar protein structures from the Protein Data Bank:** [**182**](http://www.sbkb.org/kb/report.jsp?sid=s1323380100-84&pid=structures)

**Proteins with highly similar structures in PDB**

**(**<http://zhanglab.ccmb.med.umich.edu/I-TASSER/output/S89131/>**)**

**id = 1moz_B I = 56% E = 4.4E-58 TM_Score : 0.956 RMSD 0.74**
1RRF : 1 MGNIFANLFKGLFGK-KEMRILMVGLDAAGKTTILYKLKLGEIVTTIPTIGFNVETVEYK
 MGNIF+++F L+G KE+RIL++GLD AGKTTILY+L++GE+VTT PTIGFNVET+ YK
1MOZ : 1 MGNIFSSMFDKLWGSNKELRILILGLDGAGKTTILYRLQIGEVVTTKPTIGFNVETLSYK

1RRF : NISFTVWDVGGQDKIRPLWRHYFQNTQGLIFVVDSNDRERVNEAREELMRMLAEDELRDA
 N+ VWD+GGQ IRP WR Y+ +T +IFVVDS D++R++ A +EL ML E+EL+DA
1MOZ : NLKLNVWDLGGQTSIRPYWRCYYADTAAVIFVVDSTDKDRMSTASKELHLMLQEEELQDA

1RRF : VLLVFANKQDLPNAMNAAEITDKLGLHSLRHRNWYIQATCATSGDGLYEGLDWLSNQLRN
 LLVFANKQD P A++A+E++ +L L L+ R+W I A+ A G+G+ EGLDWL + ++
1MOZ : ALLVFANKQDQPGALSASEVSKELNLVELKDRSWSIVASSAIKGEGITEGLDWLIDVIKE

1RRF : QK 181
 ++
1MOZ : EQ 182

**id = 2x77_B I = 54% E = 8.8E-49 TM_Score : 0.920 RMSD 1.70**
1RRF : 1 MGNIFANLFK--GLF-GKKEMRILMVGLDAAGKTTILYKLKLGEIVTTIPTIGFNVETVE
 MG A+L + GL +++R+LM+GLD AGKT+ILY+L LG++VTT+PT+G N+ET++
2X77 : 3 MGAWLASLKQTLGLLPADRKIRVLMLGLDNAGKTSILYRLHLGDVVTTVPTVGVNLETLQ

1RRF : YKNISFTVWDVGGQDKIRPLWRHYFQNTQGLIFVVDSNDRERVNEAREELMRMLAEDELR
 YKNISF VWD+GGQ +RP WR YF +T +I+VVDS DR+R+ A+ EL +L EDELR
2X77 : YKNISFEVWDLGGQTGVRPYWRCYFSDTDAVIYVVDSTDRDRMGVAKHELYALLDEDELR

1RRF : DAVLLVFANKQDLPNAMNAAEITDKLGLHSLRHRNWYIQATCATSGDGLYEGLDWLSNQL
 ++LL+FANKQDLP+A + AEI ++LG+ S+ +R W I + + +GDGL EG+DWL +L
2X77 : KSLLLIFANKQDLPDAASEAEIAEQLGVSSIMNRTWTIVKSSSKTGDGLVEGMDWLVERL

1RRF : RNQ 180
 R Q
2X77 : REQ 185

**id = 1mr3_F I = 76% E = 5.0E-82 TM_Score : 0.910 RMSD 1.38**
1RRF : 1 MGNIFANLFKGLFGKKEMRILMVGLDAAGKTTILYKLKLGEIVTTIPTIGFNVETVEYKN
 MG + LF LFG KEMRILMVGLD AGKTT+LYKLKLGE++TTIPTIGFNVETV+YKN
1MR3 : 1 MGLYASKLFSNLFGNKEMRILMVGLDGAGKTTVLYKLKLGEVITTIPTIGFNVETVQYKN

1RRF : ISFTVWDVGGQDKIRPLWRHYFQNTQGLIFVVDSNDRERVNEAREELMRMLAEDELRDAV
 ISFTVWDVGGQD+IR LWRHY++NT+G+IFV+DSNDR R+ EARE + RML EDELR+AV
1MR3 : ISFTVWDVGGQDRIRSLWRHYYRNTEGVIFVIDSNDRSRIGEAREVMQRMLNEDELRNAV

1RRF : LLVFANKQDLPNAMNAAEITDKLGLHSLRHRNWYIQATCATSGDGLYEGLDWLSNQLRNQ
 LVFANKQDLP AM+AAEIT+KLGLHS+R+R W+IQ+TCATSG+GLYEGL+WLSN L+NQ
1MR3 : WLVFANKQDLPEAMSAAEITEKLGLHSIRNRPWFIQSTCATSGEGLYEGLEWLSNNLKNQ

1RRF : K 181

1MR3 : S 181

**id = 1e0s_A I = 68% E = 2.6E-71 TM_Score : 0.905 RMSD 1.35**
1RRF : 6 ANLFKGLFGKKEMRILMVGLDAAGKTTILYKLKLGEIVTTIPTIGFNVETVEYKNISFTV
 + +FG KEMRILM+GLDAAGKTTILYKLKLG+ VTTIPT+GFNVETV YKN+ F V
1E0S : 1 GKVLSKIFGNKEMRILMLGLDAAGKTTILYKLKLGQSVTTIPTVGFNVETVTYKNVKFNV

1RRF : WDVGGQDKIRPLWRHYFQNTQGLIFVVDSNDRERVNEAREELMRMLAEDELRDAVLLVFA
 WDVGGQDKIRPLWRHY+ TQGLIFVVD DR+R++EAR+EL R++ + E+RDA++L+FA
1E0S : WDVGGQDKIRPLWRHYYTGTQGLIFVVDCADRDRIDEARQELHRIINDREMRDAIILIFA

1RRF : NKQDLPNAMNAAEITDKLGLHSLRHRNWYIQATCATSGDGLYEGLDWLSNQLRN 179
 NKQDLP+AM EI +KLGL +R RNWY+Q +CATSGDGLYEGL WL++ ++
1E0S : NKQDLPDAMKPHEIQEKLGLTRIRDRNWYVQPSCATSGDGLYEGLTWLTSNYKS 174

**id = 1fzq_A I = 46% E = 1.9E-37 TM_Score : 0.901 RMSD 1.66**
1RRF : 16 KEMRILMVGLDAAGKTTILYKLKLGEIVTTIPTIGFNVETVEYKNISFTVWDVGGQDKIR
 +E+RIL++GLD AGKTT+L +L +I PT GFN+++V+ + VWD+GGQ KIR
1FZQ : 15 QEVRILLLGLDNAGKTTLLKQLASEDISHITPTQGFNIKSVQSQGFKLNVWDIGGQRKIR

1RRF : PLWRHYFQNTQGLIFVVDSNDRERVNEAREELMRMLAEDELRDAVLLVFANKQDLPNAMN
 P WR YF+NT LI+V+DS DR+R E +EL +L E++L +L+FANKQDL A
1FZQ : PYWRSYFENTDILIYVIDSADRKRFEETGQELTELLEEEKLSCVPVLIFANKQDLLTAAP

1RRF : AAEITDKLGLHSLRHRNWYIQATCATSGDGLYEGLDWLSNQLRNQK 181
 A+EI + L LH++R R W IQ+ A +G+G+ +G++W+ + +K
1FZQ : ASEIAEGLNLHTIRDRVWQIQSCSALTGEGVQDGMNWVCKNVNAKK 180

**id = 2b6h_A I = 83% E = 4.5E-83 TM_Score : 0.900 RMSD 1.17**
1RRF : 6 ANLFKGLFGKKEMRILMVGLDAAGKTTILYKLKLGEIVTTIPTIGFNVETVEYKNISFTV
 +LF +FGKK+MRILMVGLDAAGKTTILYKLKLGEIVTTIPTIGFNVETVEYKNI FTV
2B6H : 18 GSLFSRIFGKKQMRILMVGLDAAGKTTILYKLKLGEIVTTIPTIGFNVETVEYKNICFTV

1RRF : WDVGGQDKIRPLWRHYFQNTQGLIFVVDSNDRERVNEAREELMRMLAEDELRDAVLLVFA
 WDVGGQDKIRPLWRHYFQNTQGLIFVVDSNDRERV E+ +EL +ML EDELRDAVLLVFA
2B6H : WDVGGQDKIRPLWRHYFQNTQGLIFVVDSNDRERVQESADELQKMLQEDELRDAVLLVFA

1RRF : NKQDLPNAMNAAEITDKLGLHSLRHRNWYIQATCATSGDGLYEGLDWLSNQL 177
 NKQD+PNAM +E+TDKLGL LR R WY+QATCAT G GLY+GLDWLS++L
2B6H : NKQDMPNAMPVSELTDKLGLQHLRSRTWYVQATCATQGTGLYDGLDWLSHEL 189

**id = 1zj6_A I = 48% E = 3.8E-48 TM_Score : 0.860 RMSD 1.79**
1RRF : 1 MGNIFANLFKGLFGKKEMRILMVGLDAAGKTTILYKLKLGEIVTTIPTIGFNVETVEYKN
 MG +F +++ LF +E ++++VGLD AGKTTILY+ + E+V T PTIG NVE + N
1ZJ6 : 1 MGILFTRIWR-LFNHQEHKVIIVGLDNAGKTTILYQFSMNEVVHTSPTIGSNVEEIVINN

1RRF : ISFTVWDVGGQDKIRPLWRHYFQNTQGLIFVVDSNDRERVNEAREELMRMLAEDELRDAV
 F +WD+GGQ+ +R W Y+ NT+ +I VVDS DRER++ REEL +MLA ++LR A
1ZJ6 : TRFLMWDIGGQESLRSSWNTYYTNTEFVIVVVDSTDRERISVTREELYKMLAHEDLRKAG

1RRF : LLVFANKQDLPNAMNAAEITDKLGLHSLRHRNWYIQATCATSGDGLYEGLDWLSNQLR 178
 LL+FANKQD+ M AEI+ L L S++ W+IQA CA +G+GL +GL+W+ ++L+
1ZJ6 : LLIFANKQDVKECMTVAEISQFLKLTSIKDHQWHIQACCALTGEGLCQGLEWMMSRLK 177

**id = 1yzg_A I = 50% E = 5.3E-49 TM_Score : 0.851 RMSD 1.67**
1RRF : 1 MGNIFANLFKGLFGKKEMRILMVGLDAAGKTTILYKLKLGEIVTTIPTIGFNVETVEYKN
 MG IFA L+ LF +E ++++VGLD AGKTTILY+ + E+V T PTIG NVE + KN
1YZG : 1 MGLIFAKLW-SLFCNQEHKVIIVGLDNAGKTTILYQFLMNEVVHTSPTIGSNVEEIVVKN

1RRF : ISFTVWDVGGQDKIRPLWRHYFQNTQGLIFVVDSNDRERVNEAREELMRMLAEDELRDAV
 F +WD+GGQ+ +R W Y+ NT+ +I VVDS DRER+ +EEL RMLA ++LR A
1YZG : THFLMWDIGGQESLRSSWNTYYSNTEFIILVVDSIDRERLAITKEELYRMLAHEDLRKAA

1RRF : LLVFANKQDLPNAMNAAEITDKLGLHSLRHRNWYIQATCATSGDGLYEGLDWLSNQL 177
 +L+FANKQD+ M AAEI+ L L S++ W+IQ+ CA +G+GL +GL+W+++++
1YZG : VLIFANKQDMKGCMTAAEISKYLTLSSIKDHPWHIQSCCALTGEGLCQGLEWMTSRI 176

**>1EZ9**

KIEEGKLVIWINGDKGYNGLAEVGKKFEKDTGIKVTVEHPDKLEEKFPQVAATGDGPDIIFWAHDRFGGYAQSGLLAEITPDKAFQDKLYPFTWDAVRYNGKLIAYPIAVEALSLIYNKDLLPNPPKTWEEIPALDKELKAKGKSALMFNLQEPYFTWPLIAADGGYAFKYENGKYDIKDVGVDNAGAKAGLTFLVDLIKNKHMNADTDYSIAEAAFNKGETAMTINGPWAWSNIDTSKVNYGVTVLPTFKGQPSKPFVGVLSAGINAASPNKELAKEFLENYLLTDEGLEAVNKDKPLGAVALKSYEEELAKDPRIAATMENAQKGEIMPNIPQMSAFWYAVRTAVINAASGRQTVDEALKDAQTRITK

**Similar protein structures from the Protein Data Bank:** [**141**](http://www.sbkb.org/kb/report.jsp?sid=s1323380251-49&pid=structures)

**Proteins with highly similar structures in PDB**

(<http://zhanglab.ccmb.med.umich.edu/I-TASSER/output/S89132/>)

**PDB Ids > 90% identity :**

**30B4; 1ANF; 1MH3; 3DM0; 3H4Z; 1HSJ; 1R6Z; 30SQ; 1Y4C; 3F5F**

**id = 2xd2_B I = 30% E = 5.1E-33**
1EZ9 : 6 KLVIWINGDKGYNG-LAEVGKKFEKDTGIKVTVEHPDKLE--EKFPQVAATGDGPDIIFW
 +L +++ D+GY + EV K +EK+ G+KVT++ D L +K G+ PD++
2XD2 : 36 ELTVYV--DEGYKSYIEEVAKAYEKEAGVKVTLKTGDALGGLDKLSLDNQNGNVPDVMMA

1EZ9 : AHDRFGGYAQSGLLAEIT-PDKAFQDKLYPFTWDAVRYNGKLIAYPIAVEALSLIYNKDL
 +DR G G L+E+ D A D A NGK+ P +E+L + YNKDL
2XD2 : PYDRVGSLGSDGQLSEVKLSDGAKTDDTTKSLVTAA--NGKVYGAPAVIESLVMYYNKDL

1EZ9 : LPNPPKTWEEIPALDKELK-----AKGKS-ALMFNLQEPYFTWPLIAADGGYAFKYENGK
 + + PKT+ ++ L K+ K GK+ A + + Y+T+ L+A +G Y F +NGK
2XD2 : VKDAPKTFADLENLAKDSKYAFAGEDGKTTAFLADWTNFYYTYGLLAGNGAYVFG-QNGK

1EZ9 : YDIKDVGVDNAGAKAGLTFLVDLIKN--KHMNADTDYS--IAEAAFNKGETAMTINGPWA
 D KD+G+ N G+ G+ + + K M DT+ + + + F +G+TA I+GPW
2XD2 : -DAKDIGLANDGSIVGINYAKSWYEKWPKGMQ-DTEGAGNLIQTQFQEGKTAAIIDGPWK

1EZ9 : WSNIDTSKVNYGVTVLPTF-KGQPSKPFVGVLSAGINAASPNKELAKEFLENYLLTDEGL
 +KVNYGV +PT G+ F G + I A N E +++F++ + T++
2XD2 : AQAFKDAKVNYGVATIPTLPNGKEYAAFGGGKAWVIPQAVKNLEASQKFVDFLVATEQQK

1EZ9 : EAVNKDKPLGAVALKSYEEELAKDPRIAATMENAQKGEIMPNIPQMSAFWYAVRTAVINA
 +K + A E D A ++ + + +PNI QMSA W + + +A
2XD2 : VLYDKTNEIPANTEARSYAEGKNDELTTAVIKQFKNTQPLPNISQMSAVWDPAKNMLFDA

1EZ9 : ASGRQTVDEALKDAQTRI 368
 SG++ A DA T I
2XD2 : VSGQKDAKTAANDAVTLI 406

**id = 2zyo_A I = 30% E = 7.5E-39**
1EZ9 : 6 KLVIWINGDKG--YNGLAEVGKKFEKDTGIKVTVEHPD--KLEEKFPQVAATGDGPDIIF
 KLV+W N D G N + +F K TGI+V V K +EK G G D++
2XYO : 20 KLVVWENADDGVQLNNTKKWAGEFTKKTGIQVEVVPVALLKQQEKLTLDGPAGKGADLVT

1EZ9 : WAHDRFGGYAQSGLLAEITPDKAFQDKLYPFTWDAVRYNGKLIAYPIAVEALSLIYNKDL
 W HDR G GLL I D + +++ A+ Y GKL P A+E+++LIYNK L
2XYO : WPHDRLGEAVTKGLLQPIQVDNSVKNQFDDVAMKALTYGGKLYGLPKAIESVALIYNKKL

1EZ9 : LPNPPKTWEEIPALDKELKA--KGKSALMFNLQEPYFTWPLIAADGGYAFKYENGKYDIK
 + P T++E+ K + K ++F Y+T+ L AA G FK ++G D
2XYO : MGQVPATYDELFQYAKANNKPDEQKYGVLFEANNFYYTYFLFAAKGAAVFKEQDGTLDPN

1EZ9 : DVGVDNAGAKAGLTFLVDLIKNKHMNADTDYSIAEAAFNKGETAMTINGPWAWSNIDTSK
 ++G+++ A G+ + + F G+ A INGPWA + +
2XYO : EIGLNSPEAVQGMNEVQKWFTEARLPQSLKADTVNGLFKSGKVAAVINGPWAIKDYQAAG

1EZ9 : VNYGVTVLPTFKGQPSKPFVGVLSAGINAASPNKELAKEFLENYLLTDEGLEAVNKDKPL
 +N GV LP G+ ++ F+GV ++A S + A E ++ +L + E L ++ K
2XYO : INVGVAPLPKIDGKDAQTFIGVKGWYLSAYSKYPKYATELMQ-FLTSKEAL--ASRFKET

1EZ9 : GAVALKSYEEELAKDPRIAAT------MENAQKGEIMPNIPQMSAFWYAVRTAVINAASG
 G + ++EL DP I + A KG MP+IP+M W + A A G
2XYO : GEI---PPQKELLNDPMIKNNPVVNGFAKQASKGVPMPSIPEMGVVWEPINNAHTFVAQG

1EZ9 : RQTVDEALKDA 364
 +QT ++AL DA
2XYO : KQTPEQALNDA 384

**>5P21**

MTEYKLVVVGAGGVGKSALTIQLIQNHFVDEYDPTIEDSYRKQVVIDGETCLLDILDTAGQEEYSAMRDQYMRTGEGFLCVFAINNTKSFEDIHQYREQIKRVKDSDDVPMVLVGNKCDLAARTVESRQAQDLARSYGIPYIETSAKTRQGVEDAFYTLVREIRQH

**Similar protein structures from the Protein Data Bank:** [**324**](http://www.sbkb.org/kb/report.jsp?sid=s1323380347-88&pid=structures)

**Proteins with highly similar structures in PDB**

(<http://zhanglab.ccmb.med.umich.edu/I-TASSER/output/S89133/>)

**id = 3kko_P I = 58% E = 9.6E-45 TM_Score : 0.962 RMSD 0.99**
5P21 : 1 MTEYKLVVVGAGGVGKSALTIQLIQNHFVDEYDPTIEDSYRKQVVIDGETCLLDILDTAG
 + YKLVVVG GGVGKSALTIQ Q FVDEYDPTIEDSYRK ID + +LD+LDTAG
3KKO : 16 LPTYKLVVVGDGGVGKSALTIQFFQKIFVDEYDPTIEDSYRKHTEIDNQWAILDVLDTAG

5P21 : QEEYSAMRDQYMRTGEGFLCVFAINNTKSFEDIHQYREQIKRVKDSDDVPMVLVGNKCDL
 QEE+SAMR+QYMRTG+GFL V+++ + SFE + ++ + I RVKD + PM+LV NK DL
3KKO : QEEFSAMREQYMRTGDGFLIVYSVTDKASFEHVDRFHQLILRVKDRESFPMILVANKVDL

5P21 : A-ARTVESRQAQDLARSYGIPYIETSAKTRQ-GVEDAFYTLVREIRQ 165
 R V Q +++A Y IPYIETSAK V+ F+ LVR IRQ
3KKO : MHLRKVTRDQGKEMATKYNIPYIETSAKDPPLNVDKTFHDLVRVIRQ 182

**id = 2rap_A I = 49% E = 1.1E-36 TM_Score : 0.962 RMSD 1.04**
5P21 : 1 MTEYKLVVVGAGGVGKSALTIQLIQNHFVDEYDPTIEDSYRKQVVIDGETCLLDILDTAG
 M EYK+VV+G+GGVGKSALT+Q + F+++YDPTIED YRK++ +D +L+ILDTAG
2RAP : 1 MREYKVVVLGSGGVGKSALTVQFVTGTFIEKYDPTIEDFYRKEIEVDSSPSVLEILDTAG

5P21 : QEEYSAMRDQYMRTGEGFLCVFAINNTKSFEDIHQYREQIKRVKDSDDVPMVLVGNKCDL
 E++++MRD Y++ G+GF+ V+++ N +SF+DI R+QI RVK + VP++LVGNK DL
2RAP : TEQFASMRDLYIKNGQGFILVYSLVNQQSFQDIKPMRDQIIRVKRYEKVPVILVGNKVDL

5P21 : -AARTVESRQAQDLARSYGIPYIETSAKTRQGVEDAFYTLVREI 163
 + R V S + + LA +G P++ETSAK++ V++ F +VR++
2RAP : ESEREVSSSEGRALAEEWGCPFMETSAKSKTMVDELFAEIVRQM 164

**id = 1gua_A I = 58% E = 1.7E-52 TM_Score : 0.960 RMSD 0.94**
5P21 : 1 MTEYKLVVVGAGGVGKSALTIQLIQNHFVDEYDPTIEDSYRKQVVIDGETCLLDILDTAG
 M EYKLVV+G+GGVGKSALT+Q +Q FVDEYDPTIEDSYRKQV +D + C+L+ILDTAG
1GUA : 1 MREYKLVVLGSGGVGKSALTVQFVQGIFVDEYDPTIEDSYRKQVEVDCQQCMLEILDTAG

5P21 : QEEYSAMRDQYMRTGEGFLCVFAINNTKSFEDIHQYREQIKRVKDSDDVPMVLVGNKCDL
 E+++AMRD YM+ G+GF V++I +F D+ REQI RVKD++DVPM+LVGNKCDL
1GUA : TEQFTAMRDLYMKNGQGFALVYSITAQSTFNDLQDLREQILRVKDTEDVPMILVGNKCDL

5P21 : A-ARTVESRQAQDLARSY-GIPYIETSAKTRQGVEDAFYTLVREIRQ 165
 R V Q Q+LAR + ++E+SAK++ V + FY LVR+I +
1GUA : EDERVVGKEQGQNLARQWCNCAFLESSAKSKINVNEIFYDLVRQINR 167

**id = 3bc1_E I = 30% E = 6.2E-18 TM_Score : 0.948 RMSD 1.21**
5P21 : 5 KLVVVGAGGVGKSALTIQLIQNHFVDEYDPTIEDSYRKQVVI-----------DGETCLL
 K + +G GVGK+++ Q F ++ T+ +R++ V+ G+ L
3BC1 : 13 KFLALGDSGVGKTSVLYQYTDGKFNSKFITTVGIDFREKRVVYRANGPDGAVGRGQRIHL

5P21 : DILDTAGQEEYSAMRDQYMRTGEGFLCVFAINNTKSFEDIHQYREQIKRVKDSDDVPMVL
 + DTAG E + ++ + R GFL +F + N +SF ++ + Q++ S++ +VL
3BC1 : QLWDTAGLERFRSLTTAFFRDAMGFLLLFDLTNEQSFLNVRNWISQLQMHAYSENPDIVL

5P21 : VGNKCDLA-ARTVESRQAQDLARSYGIPYIETSAKTRQGVEDAFYTLV 160
 GNK DL R V+ +A++LA YGIPY ETSA + A L+
3BC1 : CGNKSDLEDQRAVKEEEARELAEKYGIPYFETSAANGTNISHAIEMLL 180

**id = 2ocb_A I = 34% E = 3.2E-20 TM_Score : 0.943 RMSD 1.25**
5P21 : 5 KLVVVGAGGVGKSALTIQLIQNHFVDEYDPTIEDSY-RKQVVIDGETCLLDILDTAGQEE
 K++++G GGVGKS+L + + N F + TI + + + +DG L I DTAGQE
2OCB : 9 KVILLGDGGVGKSSLMNRYVTNKFDSQAFHTIGVEFLNRDLEVDGRFVTLQIWDTAGQER

5P21 : YSAMRDQYMRTGEGFLCVFAINNTKSFEDIHQYREQI---KRVKDSDDVPMVLVGNKCDL
 + ++R + R + L F++++ +SFE++ ++++ VKD + P V++GNK D
2OCB : FKSLRTPFYRGADCCLLTFSVDDRQSFENLGNWQKEFIYYADVKDPEHFPFVVLGNKVDK

5P21 : AARTVESRQAQDLARSYG-IPYIETSAKTRQGVEDAFYTLVREI 163
 R V + +AQ G PY+ETSAK V AF VR++
2OCB : EDRQVTTEEAQTWCMENGDYPYLETSAKDDTNVTVAFEEAVRQV 172

**id = 2fn4_A I = 58% E = 1.2E-42 TM_Score : 0.939 RMSD 1.46**
5P21 : 4 YKLVVVGAGGVGKSALTIQLIQNHFVDEYDPTIEDSYRKQVVIDGETCLLDILDTAGQEE
 +KLVVVG GGVGKSALTIQ IQ++FV +YDPTIEDSY K +DG LDILDTAGQEE
2FN4 : 10 HKLVVVGGGGVGKSALTIQFIQSYFVSDYDPTIEDSYTKICSVDGIPARLDILDTAGQEE

5P21 : YSAMRDQYMRTGEGFLCVFAINNTKSFEDIHQYREQIKRVKDSDDVPMVLVGNKCDL-AA
 + AMR+QYMR G GFL VFAIN+ +SF ++ + QI RVKD DD P+VLVGNK DL +
2FN4 : FGAMREQYMRAGHGFLLVFAINDRQSFNEVGKLFTQILRVKDRDDFPVVLVGNKADLESQ

5P21 : RTVESRQAQDLARSYGIPYIETSAKTRQGVEDAFYTLVREIRQH 166
 R V +A S+ + Y E SAK R V++AF LVR +R++
2FN4 : RQVPRSEASAFGASHHVAYFEASAKLRLNVDEAFEQLVRAVRKY 173

**id = 1yzl_A I = 33% E = 4.4E-22 TM_Score : 0.938 RMSD 1.41**
5P21 : 4 YKLVVVGAGGVGKSALTIQLIQNHFVDEYDPTIEDSY-RKQVVIDGETCLLDILDTAGQE
 +K++++G GGVGKS+L + + N F + TI + K + +DG + I DTAGQE
1YZL : 12 FKIILLGDGGVGKSSLMNRYVTNKFDSQLFHTIGVEFLNKDLEVDGHFVTMQIWDTAGQE

5P21 : EYSAMRDQYMRTGEGFLCVFAINNTKSFEDIHQYREQI---KRVKDSDDVPMVLVGNKCD
 + ++R + R + L F++++++SF+++ ++++ VK+ + P V++GNK D
1YZL : RFRSLRTPFYRGSDCCLLTFSVDDSQSFQNLSNWKKEFIYYADVKEPESFPFVILGNKTD

5P21 : LAARTVESRQAQDLARSYG-IPYIETSAKTRQGVEDAFYTLVREI 163
 + R V + +AQ + G PY ETSAK V AF VR I
1YZL : IKERQVSTEEAQAWCKDNGDYPYFETSAKDSTNVAAAFEEAVRRI 176

**id = 2bme_D I = 32% E = 4.2E-17 TM_Score : 0.937 RMSD 1.25**
5P21 : 4 YKLVVVGAGGVGKSALTIQLIQNHFVDEYDPTIEDSYRKQVV-IDGETCLLDILDTAGQE
 +K +V+G G GKS L Q I+ F D+ + TI + +++ + G+ L I DTAGQE
2BME : 11 FKFLVIGNAGTGKSCLLHQFIEKKFKDDSNHTIGVEFGSKIINVGGKYVKLQIWDTAGQE

5P21 : EYSAMRDQYMRTGEGFLCVFAINNTKSFEDIHQYREQIKRVKDSDDVPMVLVGNKCDL-A
 + ++ Y R G L V+ I + +++ + + R+ S ++ ++L GNK DL A
2BME : RFRSVTRSYYRGAAGALLVYDITSRETYNALTNWLTD-ARMLASQNIVIILCGNKKDLDA

5P21 : ARTVESRQAQDLARSYGIPYIETSAKTRQGVEDAFYTLVREI 163
 R V +A A+ + ++ETSA T + VE+AF R+I
2BME : DREVTFLEASRFAQENELMFLETSALTGENVEEAFVQCARKI 171

**id = 3rab_A I = 31% E = 2.9E-17 TM_Score : 0.935 RMSD 1.31**
5P21 : 4 YKLVVVGAGGVGKSALTIQLIQNHFVDEYDPTIEDSYR-KQVVIDGETCLLDILDTAGQE
 +K++++G VGK++ + + F + T+ ++ K + + + L I DTAGQE
3RAB : 6 FKILIIGNSSVGKTSFLFRYADDSFTPAFVSTVGIDFKVKTIYRNDKRIKLQIWDTAGQE

5P21 : EYSAMRDQYMRTGEGFLCVFAINNTKSFEDIHQYREQIKRVKDSDDVPMVLVGNKCDLA-
 Y + Y R GF+ ++ I N +SF + + QIK D+ ++LVGNKCD+
3RAB : RYRTITTAYYRGAMGFILMYDITNEESFNAVQDWSTQIK-TYSWDNAQVLLVGNKCDMED

5P21 : ARTVESRQAQDLARSYGIPYIETSAKTRQGVEDAFYTLV 160
 R V S + + LA G + E SAK V+ F LV
3RAB : ERVVSSERGRQLADHLGFEFFEASAKDNINVKQTFERLV 163

**α + β class of proteins**

| **X-ray PDB** | **NMR PDB** | ***Region of Structural Variation*** | ***Link to TASSER results*** |
| --- | --- | --- | --- |
| **2SAK** | 1SSN | 38: 48 - ELLSPHYVEFP  76: 81 - FRVVEL | <http://zhanglab.ccmb.med.umich.edu/I-TASSER/output/S89226/> |
| **1QVE** | 1HPW | 31: 55 - AQLSEAMTLASGLKTKVSDIFSQDG  78: 88 - VAKVTTGGTA | <http://zhanglab.ccmb.med.umich.edu/I-TASSER/output/S89227/> |
| **1C44** | 1QND | 90: 95 - PQSAFF  99: 102- LKIT  105: 112- MGLAMKLQ | <http://zhanglab.ccmb.med.umich.edu/I-TASSER/output/S89266/> |
| **3IL8** | 1IKM | 19: 28 - PKFIKELRVI  66: 72 - LKRAENS | <http://zhanglab.ccmb.med.umich.edu/I-TASSER/output/S89238/> |
| **1TN3** | 1RJH | 58: 68 - MKCFLAFTQTK  102: 104- SV  147: 149- KT | <http://zhanglab.ccmb.med.umich.edu/I-TASSER/output/S89237/> |

**>2SAK**

SYFEPTGPYLMVNVTGVDSKGNELLSPHYVEFPIKPGTTLTKEKIEYYVEWALDATAYKEFRVVELDPSAKIEVTYYDKNKKKEETKSFPITEKGFVVPDLSEHIKNPGFNLITKVVIEKK

**Similar protein structures from the Protein Data Bank:** [**7**](http://www.sbkb.org/kb/report.jsp?sid=s1323373410-87&pid=structures)

**Proteins with highly similar structures in PDB**

(<http://zhanglab.ccmb.med.umich.edu/I-TASSER/output/S89226/>)

WITH LESS SEQUENCE IDENTITY.

1L4Z; 1BML; 1QQR; 1JSW;

**>1QVE**

ISEFARAQLSEAMTLASGLKTKVSDIFSQDGSCPANTAATAGIEKDTDINGKYVAKVTTGGTAAASGGCTIVATMKASDVATPLRGKTLTLTLGNADKGSYTWACTSNADNKYLPKTCQTATTTTP

**Similar protein structures from the Protein Data Bank:** [**14**](http://www.sbkb.org/kb/report.jsp?sid=s1323373568-28&pid=structures)

**Proteins with highly similar structures in PDB**

(<http://zhanglab.ccmb.med.umich.edu/I-TASSER/output/S89227/>)

**id = 1ay2_A I = 31% E = 7.8E-10 TM_Score : 0.762 RMSD 2.10**
1QVE : 5 ARAQLSEAMTLASGLKTKVSDIFSQDGSCPANTAATAGIEKDTDINGKYVAKVTTGGTAA
 ARAQ+SEA+ LA G K+ V++ + G P N + +DI GKYV +V
1AY2 : 29 ARAQVSEAILLAEGQKSAVTEYYLNHGKWPENNTSAGVASPPSDIKGKYVKEVEVKNG--

1QVE : ASGGCTIVATMKASDVATPLRGKTLTLTLGNADKGSYTWACTS-----------------
 + ATM +S V ++GK L+L + GS W C
1AY2 : -----VVTATMLSSGVNNEIKGKKLSL-WARRENGSVKWFCGQPVTRTDDDTVADAKDGK

1QVE : NADNKYLPKTCQ 119
 D K+LP TC+
1AY2 : EIDTKHLPSTCR 152

**id = 2hi2_A I = 31% E = 3.3E-10**
1QVE : 5 ARAQLSEAMTLASGLKTKVSDIFSQDGSCPANTAATAGIEKDTDINGKYVAKVTTGGTAA
 ARAQ+SEA+ LA G K+ V++ + G P N + TDI GKYV +V
2HI2 : 29 ARAQVSEAILLAEGQKSAVTEYYLNHGKWPENNTSAGVASSPTDIKGKYVKEVEV-----

1QVE : ASGGCTIVATMKASDVATPLRGKTLTLTLGNADKGSYTWACTS-----------------
 + ATM +S V ++GK L+L + GS W C
2HI2 : --KNGVVTATMLSSGVNNEIKGKKLSL-WARRENGSVKWFCGQPVTRTDDDTVADAKDGK

1QVE : NADNKYLPKTCQ 119
 D K+LP TC+
2HI2 : EIDTKHLPSTCR 152

**id = 1x6z_A I = 29% E = 2.2E-4**
1QVE : 2 SEFARAQLSEAMTLASGLKTKVSDIFSQDGSCPANTAATAGIEKDTDINGKYVAKVTTGG
 +EFAR++ + A+ + LKT V + S+ S + T +K+ + A G
1X6Z : 5 TEFARSEGASALASVNPLKTTVEEALSRGWSVKSGTGTEDATKKEVPLG--VAADANKLG

1QVE : TAA-----ASGGCTIVATMKASDVATPLRGKTLTLTLGNADKGSYTWACTSNADNKYLPK
 T A A G I T +GK +TLT AD W CTS+ D +++PK
1X6Z : TIALKPDPADGTADITLTFTMGGAGPKNKGKIITLTRTAADG---LWKCTSDQDEQFIPK

1QVE : TC 118
 C
1X6Z : GC 121

**>1C44**

SSAGDGFKANLVFKEIEKKLEEEGEQFVKKIGGIFAFKVKDGPGGKEATWVVDVKNGKGSVLPNSDKKADCTITMADSDLLALMTGKMNPQSAFFQGKLKITGNMGLAMKLQNLQLQPGKAKL

**Similar protein structures from the Protein Data Bank:** [**4**](http://www.sbkb.org/kb/report.jsp?sid=s1323373712-26&pid=structures)

**Proteins with highly similar structures in PDB**

(<http://zhanglab.ccmb.med.umich.edu/I-TASSER/output/S89266/>)

**id = 1ikt_A I = 38% E = 9.9E-17**
1C44 : 4 GDGFKANLVFKEIEKKLEEEGEQFVKKIGGIFAFKVKDGPGGKEATWVVDVKNGKGSVLP
 G ++ VF+EI ++L++ G + VKK+ +F + + G G A W +D+K+G G V
1IKT : 3 GGKLQSTFVFEEIGRRLKDIGPEVVKKVNAVFEWHITKG-GNIGAKWTIDLKSGSGKVYQ

1C44 : NSDK-KADCTITMADSDLLALMTGKMNPQSAFFQGKLKITGNMGLAMKLQNL 114
 K AD TI ++D D + ++ GK++PQ AFF G+LK GN+ L+ KLQ +
1IKT : GPAKGAADTTIILSDEDFMEVVLGKLDPQKAFFSGRLKARGNIMLSQKLQMI 113

**>3IL8**

SAKELRCQCIKTYSKPFHPKFIKELRVIESGPHCANTEIIVKLSDGRELCLDPKENWVQRVVEKFLKRAENS

**Similar protein structures from the Protein Data Bank:** [26](http://www.sbkb.org/kb/report.jsp?sid=s1323373999-53&pid=structures)

**Proteins with highly similar structures in PDB**

(<http://zhanglab.ccmb.med.umich.edu/I-TASSER/output/S89238/>)

**id = 1mgs_B I = 47% E = 6.9E-13 TM_Score : 0.797 RMSD 1.71**
3IL8 : 2 AKELRCQCIKTYSKPFHPKFIKELRVIESGPHCANTEIIVKLSDGRELCLDPKENWVQRV
 A ELRCQC++T + HPK I+ + V GPHCA TE+I L +GR+ CL+P V+++
1MGS : 4 ATELRCQCLQTL-QGIHPKNIQSVNVKSPGPHCAQTEVIATLKNGRKACLNPASPIVKKI

3IL8 : VEKFL 66
 +EK L
1MGS : IEKML 67

**id = 1rod_B I = 86% E = 1.7E-28**
3IL8 : 1 SAKELRCQCIKTYSKPFHPKFIKELRVIESGPHCANTEIIVKLSDGRELCLDPKENWVQR
 SAKELRCQCIKTYSKPFHPKFIKELRVIESGPHCANTEIIVKLSDGRELCLDP V++
1ROD : 1 SAKELRCQCIKTYSKPFHPKFIKELRVIESGPHCANTEIIVKLSDGRELCLDPASPIVKK

3IL8 : VVEKFL 66
 ++EK L
1ROD : IIEKML 66

**id = 1f9p_A I = 47% E = 2.2E-12 TM_Score : 0.770 RMSD 1.95**
3IL8 : 4 ELRCQCIKTYSKPFHPKFIKELRVIESGPHCANTEIIVKLSDGRELCLDPKENWVQRVVE
 ELRC CIKT S HPK I+ L VI G HC E+I L DGR++CLDP ++++V+
1F9P : 17 ELRCMCIKTTS-GIHPKNIQSLEVIGKGTHCNQVEVIATLKDGRKICLDPDAPRIKKIVQ

3IL8 : KFLKRAENS 72
 K L E++
1F9P : KKLAGDESA 84

**id = 1nap_D I = 47% E = 4.6E-12 TM_Score : 0.761 RMSD 1.82**
3IL8 : 4 ELRCQCIKTYSKPFHPKFIKELRVIESGPHCANTEIIVKLSDGRELCLDPKENWVQRVVE
 ELRC CIKT S HPK I+ L VI G HC E+I L DGR++CLDP ++++V+
1NAP : 2 ELRCLCIKTTSG-IHPKNIQSLEVIGKGTHCNQVEVIATLKDGRKICLDPDAPRIKKIVQ

3IL8 : KFLKRAENS 72
 K L E++
1NAP : KKLAGDESA 69

**id = 1qnk_B I = 42% E = 9.8E-12 TM_Score : 0.750 RMSD 1.76**
3IL8 : 4 ELRCQCIKTYSKPFHPKFIKELRVIESGPHCANTEIIVKLSDGRELCLDPKENWVQRVVE
 ELRCQC++T + H K I+ ++V GPHCA TE+I L +G++ CL+P V++++E
1GNK : 2 ELRCQCLQTL-QGIHLKNIQSVKVKSPGPHCAQTEVIATLKNGQKACLNPASPMVKKIIE

3IL8 : KFLKRAENS 72
 K LK +++
1GNK : KMLKNGKSN 69

**id = 1tvx_D I = 47% E = 4.6E-12**
3IL8 : 4 ELRCQCIKTYSKPFHPKFIKELRVIESGPHCANTEIIVKLSDGRELCLDPKENWVQRVVE
 ELRC CIKT S HPK I+ L VI G HC E+I L DGR++CLDP ++++V+
1TVX : 7 ELRCLCIKTTSG-IHPKNIQSLEVIGKGTHCNQVEVIATLKDGRKICLDPDAPRIKKIVQ

3IL8 : KFLKRAENS 72
 K L E++
1TVX : KKLAGDESA 74

**>1TN3**

ALQTVCLKGTKVHMKCFLAFTQTKTFHEASEDCISRGGTLSTPQTGSENDALYEYLRQSVGNEAEIWLGLNDMAAEGTWVDMTGARIAYKNWETEITAQPDGGKTENCAVLSGAANGKWFDKRCRDQLPYICQFGIV

**Similar protein structures from the Protein Data Bank:** [127](http://www.sbkb.org/kb/report.jsp?sid=s1323374078-38&pid=structures)

**Proteins with highly similar structures in PDB**

(<http://zhanglab.ccmb.med.umich.edu/I-TASSER/output/S89237/>)

**id = 3l9j_C I = 86% E = 1.2E-63 TM_Score : 0.848 RMSD 1.52**
1TN3 : 2 LQTVCLKGTKVHMKCFLAFTQTKTFHEASEDCISRGGTLSTPQTGSENDALYEYLRQSVG
 LQTVCLKGTK HMKCFLAFTQTKTFHEASEDCISRGGTLSTPQTGSENDALYEYLRQSVG
319J : 1 LQTVCLKGTK-HMKCFLAFTQTKTFHEASEDCISRGGTLSTPQTGSENDALYEYLRQSVG

1TN3 : NEAEIWLGLNDMAAEGTWVDMTGARIAYKNWETEITAQPDGGKTENCAVLSGAANGKWFD
 NEAEIWLGLN + WVDMTG RIAYKNWE AQPD ENCAVLSGAANGKWF
319J : NEAEIWLGLNKRWSRYFWVDMTGTRIAYKNWEHSSDAQPDPSNWENCAVLSGAANGKWFG

1TN3 : KRCRDQLPYICQFGI 136
 KRCRDQLPYICQFGI
319J : KRCRDQLPYICQFGI 134

**id = 1wmy_B I = 22% E = 1.8E-05 TM_Score : 0.823 RMSD 1.94**
1TN3 : 15 KCFLAFTQTKTFHEASEDCISRG-------GTLSTPQTGSENDALYEYLRQSVGNEAEIW
 C+ F T+ A +C+S L + + +E ++ Y R ++W
1WMY : 13 HCYRFFNTLTTWENAHHECVSYSCSTLNVRSDLVSVHSAAEQAYVFNYWRGIDSQAGQLW

1TN3 : LGLNDMAAEGTWVDMTGARIAYKNWETEITAQPDG-GKTENCAVLSGAANGKWFDKRCRD
 +GL D EG ++ G+++ Y W QPD E+ G W D
1WMY : IGLYDKYNEGDFIWTDGSKVGYTKW---AGGQPDNWNNAEDYGQFRHTEGGAWNDNSAAA

1TN3 : QLPYICQF 134
 Q Y+C+
1WMY : QAKYMCKL 137

**id = 2ox9_D I = 29% E = 3.3E-06 TM_Score : 0.817 RMSD 1.70**
1TN3 : 15 KCFLAFTQTKTFHEASEDCISRGGTLSTPQTGSENDALYEYLRQSVGNEAEIWLGLNDMA
 KC+ + + F +A C + L + E + + +VG E+ W+GL D
2OX9 : 15 KCYYFSLEKEIFEDAKLFCEDKSSHLVFINSREEQQWI---KKHTVGRESH-WIGLTDSE

1TN3 : AEGTWVDMTGARIAYKNWETEITAQPDG-----GKTENCAVLSGAANGKWFDKRCRDQLP
 E W + G+ + YKNW+ QPD G E+CA L A G+W D +C +
2OX9 : QESEWKWLDGSPVDYKNWK---AGQPDNWGSGHGPGEDCAGLIYA--GQWNDFQCDEINN

1TN3 : YICQ 133
 +IC+
2OX9 : FICE 129

**id = 1lit_A I = 22% E = 1.9E-05 TM_Score : 0.817 RMSD 2.15**
1TN3 : 6 CLKGTKVHMK-CFLAFTQTKTFHEASEDCIS-RGGTLSTPQTGSENDALYEYLRQSVGNE
 C +GT + C+ +T+ +A C + G L + T +E + +++S ++
1LIT : 14 CPEGTNAYRSYCYYFNEDRETWVDADLYCQNMNSGNLVSVLTQAEGAFVASLIKESGTDD

1TN3 : AEIWLGLNDMAAEGTWVDMTGARIAYKNWETEITAQPDGGKTENCAVLSGAANGKWFDKR
 +W+GL+D W +G+ ++YK+W + + G ++ S KW D
1LIT : FNVWIGLHDPKKNRRWHWSSGSLVSYKSWGIGAPSSVNPGYC--VSLTSSTGFQKWKDVP

1TN3 : CRDQLPYICQF 134
 C D+ ++C+F
1LIT : CEDKFSFVCKF 142

**id = 2ox8_D I = 31% E = 6.8E-07 TM_Score : 0.803 RMSD 1.66**
1TN3 : 15 KCFLAFTQTKTFHEASEDCISRGGTLSTPQTGSENDALYEYLRQSVGNEAEIWLGLNDMA
 KC+ + + F +A C + L T E + +Q VG E+ W+GL D
2OX8 : 15 KCYYFSVEKEIFEDAKLFCEDKSSHLVFINTREEQQWI---KKQMVGRESH-WIGLTDSE

1TN3 : AEGTWVDMTGARIAYKNWETEITAQPDG-----GKTENCAVLSGAANGKWFDKRCRDQLP
 E W + G YKNW+ QPD G E+CA L A G+W D +C D
2OX8 : RENEWKWLDGTSPDYKNWK---AGQPDNWGHGHGPGEDCAGLIYA--GQWNDFQCEDVNN

1TN3 : YICQ 133
 +IC+
2OX8 : FICE 129

**id = 2vuv_A I = 26% E = 8.5E-4 TM_Score : 0.802 RMSD 2.00**
1TN3 : 16 CFLAFTQTKTFHEASEDCISRGGTLSTPQTGSENDALYEYLRQSVGNEAEI--WLGLNDM
 C++ + ++ A C + GG L+ P T EN+ L +++ G+ WLG +
2VUV : 13 CYIYQSAKASWASAQSSCQALGGILAEPDTACENEVLIHMCKEN-GDAGSFGPWLGGQKV

1TN3 : AAEGTWVDMTGARIAYKNWETEITAQP-DGGKTENCAVLSGAANGKWFDKRCRDQLPYIC
 W +GA Y W +P + G E+C + + W D RC Q Y+C
2VUV : GGAWQWSS-SGAAFDYLRWGPN---EPNNSGGNEDCLHYNWLS---WNDLRCHYQASYLC

1TN3 : Q 133
 Q
2VUV : Q 125

**Small proteins**

| ***X-ray PDB*** | ***NMR PDB*** | ***Region of Structural Variation*** | ***Link to TASSER results*** |
| --- | --- | --- | --- |
| **1PSP** | 1PCP | 5: 10 - ACRCSR  13: 15 - PKN  55: 59 - SEECV  61: 64 - QVSA | <http://zhanglab.ccmb.med.umich.edu/I-TASSER/output/S89302/> |
| **1NTN** | 1W6B | 50: 52 - ESY  62: 68 - NCNPHPK | <http://zhanglab.ccmb.med.umich.edu/I-TASSER/output/S89268/> |
| **1BRF** | 1RWD | 2: 7 - KWVCKI  11: 13 - IYD  45: 50 - KSEFEK | <http://zhanglab.ccmb.med.umich.edu/I-TASSER/output/S89269/> |
| **9PTI** | 1OA5 | 3: 6 - DFCL  48: 56 - AEDCMRTCG | <http://zhanglab.ccmb.med.umich.edu/I-TASSER/output/S89304/> |
| **1RDG** | 1E8J | 11: 13 - YEY  20: 22 - PDS  30: 33 - FEDL  46: 48 - KDA | <http://zhanglab.ccmb.med.umich.edu/I-TASSER/output/S89306/> |

**>1PSP**

EKPAACRCSRQDPKNRVNCGFPGITSDQCFTSGCCFDSQVPGVPWCFKPLPAQESEECVMQVSARKNCGYPGISPEDCAARNCCFSDTIPEVPWCFFPMSVEDCHY

**Similar protein structures from the Protein Data Bank:** [**8**](http://www.sbkb.org/kb/report.jsp?sid=s1323380653-14&pid=structures)

**Proteins with highly similar structures in PDB**

(<http://zhanglab.ccmb.med.umich.edu/I-TASSER/output/S89302/>)

**id = 1e9t_A I = 62% E = 5.3E-10**
1PSP : 14 KNRVNCGFPGITSDQCFTSGCCFDSQVPGVPWCFKPLPAQESE 56
 K+RV+CG+P +T +C GCCFDS++PGVPWCFKPL QE+E
1E9T : 16 KDRVDCGYPHVTPKECNNRGCCFDSRIPGVPWCFKPL--QEAE 56

id = 1e9t_A I = 40% E = 8.4E-08
1PSP : 55 SEECVMQVSARKNCGYPGISPEDCAARNCCFSDTIPEVPWCFFPMSVEDCHY 106
 + +C + R +CGYP ++P++C R CCF IP VPWCF P+ +C +
1E9T : 8 ANQCAVPAKDRVDCGYPHVTPKECNNRGCCFDSRIPGVPWCFKPLQEAECTF 59

**id = 1pe3_2 I = 62% E = 5.3E-10**
1PSP : 14 KNRVNCGFPGITSDQCFTSGCCFDSQVPGVPWCFKPLPAQESE 56
 K+RV+CG+P +T +C GCCFDS++PGVPWCFKPL QE+E
1PE3 : 16 KDRVDCGYPHVTPKECNNRGCCFDSRIPGVPWCFKPL--QEAE 56

id = 1pe3_2 I = 40% E = 8.4E-08
1PSP : 55 SEECVMQVSARKNCGYPGISPEDCAARNCCFSDTIPEVPWCFFPMSVEDCHY 106
 + +C + R +CGYP ++P++C R CCF IP VPWCF P+ +C +
1PE3 : 8 ANQCAVPAKDRVDCGYPHVTPKECNNRGCCFDSRIPGVPWCFKPLQEAECTF 59

**id = 1ps2_A I = 57% E = 9.5E-09**1PSP : 13 PKNRVNCGFPGITSDQCFTSGCCFDSQVPGVPWCFKPL-----PAQESE 56
 P+ R NCGFPG+T QC GCCFD V GVPWCF P P +ESE
1PS2 : 11 PRERQNCGFPGVTPSQCANKGCCFDDTVRGVPWCFYPNTIDVPPEEESE 59

**id = 1ps2_A I = 44% E = 1.4E-09**
1PSP : 54 ESEECVMQVSARKNCGYPGISPEDCAARNCCFSDTIPEVPWCFFPMSVE 102
 ++E C + R+NCG+PG++P CA + CCF DT+ VPWCF+P +++
1PS2 : 3 QTETCTVAPRERQNCGFPGVTPSQCANKGCCFDDTVRGVPWCFYPNTID 51

**id = 1hi7_B I = 64% E = 1.5E-08**
1PSP : 13 PKNRVNCGFPGITSDQCFTSGCCFDSQVPGVPWCFKP 49
 P+ R NCGFPG+T QC GCCFD V GVPWCF P
1HI7 : 11 PRERQNCGFPGVTPSQCANKGCCFDDTVRGVPWCFYP 47

**id = 1hi7_B I = 44% E = 1.7E-09**1PSP : 54 ESEECVMQVSARKNCGYPGISPEDCAARNCCFSDTIPEVPWCFFPMSVE 102
 ++E C + R+NCG+PG++P CA + CCF DT+ VPWCF+P +++
Sbjct: 3 QTETCTVAPRERQNCGFPGVTPSQCANKGCCFDDTVRGVPWCFYPNTID 51

**>1NTN**

ITCYKTPIITSETCAPGQNLCYTKTWCDAWCGSRGKVIELGCAATCPTVESYQDIKCCSTDNCNPHPKQKRP

**Similar protein structures from the Protein Data Bank:** [**55**](http://www.sbkb.org/kb/report.jsp?sid=s1323380686-49&pid=structures)

**Proteins with highly similar structures in PDB**

(<http://zhanglab.ccmb.med.umich.edu/I-TASSER/output/S89268/>)

**id = 1hc9_B I = 62% E = 3.3E-21 TM_Score : 0.875 RMSD 1.49**
1NTN : 1 ITCYKTPI--ITSETCAPGQNLCYTKTWCDAWCGSRGKVIELGCAATCPTVESYQDIKCC
 I C+ T I++ TC PG+NLCY K WCDA+C SRGKV+ELGCAATCP+ + Y+++ CC
1HC9 : 1 IVCHTTATSPISAVTCPPGENLCYRKMWCDAFCSSRGKVVELGCAATCPSKKPYEEVTCC

1NTN : STDNCNPHPKQK 70
 STD CNPHPKQ+
1HC9 : STDKCNPHPKQR 72

**id = 3neq_B I = 44% E = 2.1E-4 TM_Score : 0.776 RMSD 1.33**
1NTN : 1 ITCYKTPII---TSETCAPGQNLCYTKTWCDAWCGSRGKVIELGCAATCPTVESYQD-IK
 +TC K+ I TSE C GQNLC+ K W + R GCAATCP + ++ I+
3NEQ : 1 LTCVKSNSIWFPTSEDCPDGQNLCF-KRW--QYISPRMYDFTRGCAATCPKPTNVRETIR

1NTN : CCSTDNCN 64
 CC TD CN
3NEQ : CCGTDKCN 65

**id = 1txa_A I = 69% E = 4.1E-21**
1NTN : 3 CYKTPIITSETCAPGQNLCYTKTWCDAWCGSRGKVIELGCAATCPTVESYQDIKCCSTDN
 CY TP TS+TC GQ++CYTKTWCD +C SRGK I+LGCAATCP V+ DIKCCSTDN
1TXA : 3 CYVTPDATSQTCPDGQDICYTKTWCDGFCSSRGKRIDLGCAATCPKVKPGVDIKCCSTDN

1NTN : CNPHPKQKR 71
 CNP P KR
1TXA : CNPFPTWKR 71

**>1BRF**

AKWVCKICGYIYDEDAGDPDNGISPGTKFEELPDDWVCPICGAPKSEFEKLED

**Similar protein structures from the Protein Data Bank:** [**61**](http://www.sbkb.org/kb/report.jsp?sid=s1323380754-38&pid=structures)

**Proteins with highly similar structures in PDB**

(<http://zhanglab.ccmb.med.umich.edu/I-TASSER/output/S89269/>)

**id = 2kn9_A I = 54% E = 1.7E-09 TM_Score : 0.851 RMSD 0.98**
1BRF : 5 CKICGYIYDEDAGDPDNGISPGTKFEELPDDWVCPICGAPKSEFEKLE 52
 C CG+ YDE G P++GI+ GT+++++PDDW CP CGA KS+FE +E
2KN9 : 30 CIQCGFEYDEALGWPEDGIAAGTRWDDIPDDWSCPDCGAAKSDFEMVE 77

**id = 1b13_A I = 55% E = 7.8E-14 TM_Score : 0.845 RMSD 1.18**
1BRF : 2 KWVCKICGYIYDEDAGDPDNGISPGTKFEELPDDWVCPICGAPKSEFEKLED 53
 K+ C +C YIY+ + GDPDNG++PGT F+++PDDWVCP+CG K +FE++E+
1B13 : 3 KYTCTVCAYIYNPEDGDPDNGVNPGTDFKDIPDDWVCPLCGVGKDQFEEVEE 54

**id = 2pvx_H I = 86% E = 4.5E-21 TM_Score : 0.841 RMSD 1.19**
1BRF : 2 KWVCKICGYIYDEDAGDPDNGISPGTKFEELPDDWVCPICGAPKSEFEKLED 53
 KWVC +CGYIYDEDAGDPDNGISPGTKFEELPDDWVCP+CG K +FEKLED
2PVX : 3 KWVCTVCGYIYDEDAGDPDNGISPGTKFEELPDDWVCPLCGVGKDQFEKLED 54


**id = 2v3b_B I = 54% E = 5.7E-11 TM_Score : 0.829 RMSD 1.10**
1BRF : 2 KWVCKICGYIYDEDAGDPDNGISPGTKFEELPDDWVCPICGAPKSEFEKLE 52
 KW C +CG+IYDE G P+ GI GT++E++P DWVCP CG K +FE +E
2V3B : 3 KWQCVVCGFIYDEALGLPEEGIPAGTRWEDIPADWVCPDCGVGKIDFEMIE 53

**id = 1s24_A I = 53% E = 1.9E-11 TM_Score : 0.815 RMSD 1.34**
1BRF : 2 KWVCKICGYIYDEDAGDPDNGISPGTKFEELPDDWVCPICGAPKSEFEKLED 53
 KW+C CG+IYDE GD G +PGT+FE++PDDW CP CGA K ++ E+
1S24 : 35 KWICITCGHIYDEALGDEAEGFTPGTRFEDIPDDWCCPDCGATKEDYVLYEE 86

**id = 2pve_C I = 71% E = 2.3E-17 TM_Score : 0.811 RMSD 1.29**
1BRF : 2 KWVCKICGYIYDEDAGDPDNGISPGTKFEELPDDWVCPICGAPKSEFEKLED 53
 K+ CKICGYIY+ + GDPDNG++PGT F+++PDDWVCPICGAPKSEFE++E+
2PVE : 3 KYTCKICGYIYNPEDGDPDNGVNPGTDFKDIPDDWVCPICGAPKSEFEEVEE 54

**id = 2dsx_A I = 68% E = 4.0E-14 TM_Score : 0.809 RMSD 1.12**
1BRF : 3 WVCKICGYIYDEDAGDPDNGISPGTKFEELPDDWVCPICGAPKSEFEK 50
 +VC +CGY YD GDPD+GI PGTKFE+LPDDW CP+CGA K FEK
2DSX : 4 YVCTVCGYEYDPAKGDPDSGIKPGTKFEDLPDDWACPVCGASKDAFEK 51

**id = 1rdv_A I = 72% E = 2.3E-15 TM_Score : 0.809 RMSD 1.13**
1BRF : 2 KWVCKICGYIYDEDAGDPDNGISPGTKFEELPDDWVCPICGAPKSEFE 49
 K+VC +CGY YD GDPDNG+ PGT FE++P DWVCPICGAPKSEFE
1RDV : 3 KYVCTVCGYEYDPAEGDPDNGVKPGTAFEDVPADWVCPICGAPKSEFE 50

**>9PTI**

RPDFCLEPPYTGPCKARIIRYFYNAKAGLCQTFVYGGCRAKRNNFKSAEDCMRTCGGA

**Similar protein structures from the Protein Data Bank:** [**127**](http://www.sbkb.org/kb/report.jsp?sid=s1323380822-11&pid=structures)

**Proteins with highly similar structures in PDB**

(<http://zhanglab.ccmb.med.umich.edu/I-TASSER/output/S89304/>)

**id = 2ddj_A I = 41% E = 5.7E-06 TM_Score : 0.672 RMSD 0.75**
9PTI : 3 DFCLEPPYTGPCKARIIRYFYNAKAGLCQTFVYGGCRAKRNNFKSAEDCMRTC 55
 D C+ P GPC+ R+ Y+ C FVYGGC NNF S E C C
2DDJ : 8 DACVLPAVQGPCRGWEPRWAYSPLLQQCHPFVYGGCEGNGNNFHSRESCEDAC 60

**id = 1bik_A I = 41% E = 1.9E-07 TM_Score : 0.585 RMSD 2.81**
9PTI : 1 RPDFCLEPPYTGPCKARIIRYFYNAKAGLCQTFVYGGCRAKRNNFKSAEDCMRTC 55
 + D C GPC RYFYN + C+TF YGGC NNF + ++C++TC
1BIK : 22 KEDSCQLGYSAGPCMGMTSRYFYNGTSMACETFQYGGCMGNGNNFVTEKECLQTC 76

**id = 1zr0_D I = 41% E = 6.9E-07 TM_Score : 0.549 RMSD 1.96**
9PTI : 3 DFCLEPPYTGPCKARIIRYFYNAKAGLCQTFVYGGCRAKRNNFKSAEDCMRTC 55
 + CL P GPC+A ++RY+Y+ C+ F+YGGC NNF + E C C
1ZR 0: 7 EICLLPLDYGPCRALLLRYYYDRYTQSCRQFLYGGCEGNANNFYTWEACDDAC 59

**id = 1yc0_I I = 35% E = 8.2E-07 TM_Score : 0.548 RMSD 1.94**
9PTI : 3 DFCLEPPYTGPCKARIIRYFYNAKAGLCQTFVYGGCRAKRNNFKSAEDCMRTCGGA 58
 D+CL G C+ R++Y+ +C++FVYGGC +NN+ E+C+ C G
1YC0 : 20 DYCLASNKVGRCRGSFPRWYYDPTEQICKSFVYGGCLGNKNNYLREEECILACRGV 75

**id = 2ody_F I = 44% E = 1.2E-08 TM_Score : 0.525 RMSD 2.18**
9PTI : 1 RPDFCLEPPYTGPCKARIIRYFYNAKAGLCQTFVYGGCRAKRNNFKSAEDCMRTCG 56
 R FC P G CKA I R+++N + G C F YGGC NNF++ E+C + CG
2ODY : 2 RNGFCRLPADEGICKALIPRFYFNTETGKCTMFSYGGCGGNENNFETIEECQKACG 57

**id = 1tfx_D I = 43% E = 3.2E-08 TM_Score : 0.522 RMSD 1.85**
9PTI : 1 RPDFCLEPPYTGPCKARIIRYFYNAKAGLCQTFVYGGCRAKRNNFKSAEDCMRTC 55
 +PDFC G C+ I RYFYN + C+ F YGGC NNF++ E+C C
1TFX : 1 KPDFCFLEEDPGICRGYITRYFYNNQTKQCERFKYGGCLGNMNNFETLEECKNIC 55

**id = 3d65_I I = 45% E = 1.3E-09 TM_Score : 0.508 RMSD 1.50**
9PTI : 1 RPDFCLEPPYTGPCKARIIRYFYNAKAGLCQTFVYGGCRAKRNNFKSAEDCMRTCGG 57
 RPDFC P TGPC+ R ++YN C F+YGGC NNF + E+C TC
3D65 : 1 RPDFCELPADTGPCRVRFPSFYYNPDEKKCLEFIYGGCEGNANNFITKEECESTCAA 57

**>1RDG**

MDIYVCTVCGYEYDPAKGDPDSGIKPGTKFEDLPDDWACPVCGASKDAFEKQ

**Similar protein structures from the Protein Data Bank:** [**61**](http://www.sbkb.org/kb/report.jsp?sid=s1323380883-13&pid=structures)

**Proteins with highly similar structures in PDB**

(<http://zhanglab.ccmb.med.umich.edu/I-TASSER/output/S89306/>)

**id = 1bq8_A I = 66% E = 2.6E-14 TM_Score : 0.891 RMSD 0.88**
1RDG : 1 MDIYVCTVCGYEYDPAKGDPDSGIKPGTKFEDLPDDWACPVCGASKDAFEK 51
 M +VC +CGY YD GDPD+GI PGTKFE+LPDDW CP+CGA K FEK
1BQ8 : 1 MAKWVCKICGYIYDEDAGDPDNGISPGTKFEELPDDWVCPICGAPKSEFEK 51

**id = 2pvx_H I = 70% E = 2.1E-15 TM_Score : 0.883 RMSD 0.92**
1RDG : 1 MDIYVCTVCGYEYDPAKGDPDSGIKPGTKFEDLPDDWACPVCGASKDAFEK 51
 M +VCTVCGY YD GDPD+GI PGTKFE+LPDDW CP+CG KD FEK
2PVX : 1 MKKWVCTVCGYIYDEDAGDPDNGISPGTKFEELPDDWVCPLCGVGKDQFEK 51

**id = 1iro_A I = 62% E = 1.7E-14 TM_Score : 0.883 RMSD 0.93**
1RDG : 1 MDIYVCTVCGYEYDPAKGDPDSGIKPGTKFEDLPDDWACPVCGASKDAFEK 51
 M Y CTVCGY Y+P GDPD+G+ PGT F+D+PDDW CP+CG KD FE+
1IRO : 1 MKKYTCTVCGYIYNPEDGDPDNGVNPGTDFKDIPDDWVCPLCGVGKDQFEE 51

**id = 1iu5_A I = 66% E = 4.1E-14 TM_Score : 0.881 RMSD 0.84**
1RDG : 4 YVCTVCGYEYDPAKGDPDSGIKPGTKFEDLPDDWACPVCGASKDAFEK 51
 YVC +CGY YD GDPD+G+ PGTKFE++PDDW CP+CGA K FEK
1IU5 : 3 YVCKICGYIYDEDAGDPDNGVSPGTKFEEIPDDWVCPICGAPKSEFEK 50

**id = 2kn9_A I = 54% E = 2.0E-10 TM_Score : 0.876 RMSD 0.92**
1RDG : 3 IYVCTVCGYEYDPAKGDPDSGIKPGTKFEDLPDDWACPVCGASKDAFE 50
 ++ C CG+EYD A G P+ GI GT+++D+PDDW+CP CGA+K FE
2KN9 : 27 LFRCIQCGFEYDEALGWPEDGIAAGTRWDDIPDDWSCPDCGAAKSDFE 74

**id = 1rb9_A I = 76% E = 7.0E-17 TM_Score : 0.872 RMSD 0.96**
1RDG : 1 MDIYVCTVCGYEYDPAKGDPDSGIKPGTKFEDLPDDWACPVCGASKDAFE 50
 M YVCTVCGYEYDPA+GDPD+G+KPGT F+DLP DW CPVCGA K FE
1RB9 : 1 MKKYVCTVCGYEYDPAEGDPDNGVKPGTSFDDLPADWVCPVCGAPKSEFE 50

**id = 2pve_C I = 58% E = 1.3E-13 TM_Score : 0.867 RMSD 1.03**
1RDG : 1 MDIYVCTVCGYEYDPAKGDPDSGIKPGTKFEDLPDDWACPVCGASKDAFEK 51
 M Y C +CGY Y+P GDPD+G+ PGT F+D+PDDW CP+CGA K FE+
2PVE : 1 MKKYTCKICGYIYNPEDGDPDNGVNPGTDFKDIPDDWVCPICGAPKSEFEE 51

**id = 2v3b_B I = 52% E = 7.4E-09 TM_Score : 0.845 RMSD 1.08**
1RDG : 1 MDIYVCTVCGYEYDPAKGDPDSGIKPGTKFEDLPDDWACPVCGASKDAFE 50
 M + C VCG+ YD A G P+ GI GT++ED+P DW CP CG K FE
2V3B : 1 MRKWQCVVCGFIYDEALGLPEEGIPAGTRWEDIPADWVCPDCGVGKIDFE 50

**id = 1dx8_A I = 51% E = 1.3E-07 TM_Score : 0.818 RMSD 1.19**
1RDG : 4 YVCTVCGYEYDPAKGDPDSGIKPGTKFEDLPDDWACPVCGASKDAFE 50
 Y C CGY Y+P KGD +GI PGT F DL D + CP C + K+ F+
1DX8 : 8 YECEACGYIYEPEKGDKFAGIPPGTPFVDLSDSFMCPACRSPKNQFK 54
